# The transcriptional elongation factor CTR9 demarcates PRC2-mediated H3K27me3 domains by altering PRC2 subtype equilibrium

**DOI:** 10.1093/nar/gkac047

**Published:** 2022-02-07

**Authors:** Ngai Ting Chan, Junfeng Huang, Gui Ma, Hao Zeng, Kristine Donahue, Yidan Wang, Lingjun Li, Wei Xu

**Affiliations:** McArdle Laboratory for Cancer Research, University of Wisconsin-Madison, Madison, WI 53706, USA; School of Pharmacy, University of Wisconsin-Madison, Madison, WI 53705, USA; McArdle Laboratory for Cancer Research, University of Wisconsin-Madison, Madison, WI 53706, USA; McArdle Laboratory for Cancer Research, University of Wisconsin-Madison, Madison, WI 53706, USA; McArdle Laboratory for Cancer Research, University of Wisconsin-Madison, Madison, WI 53706, USA; McArdle Laboratory for Cancer Research, University of Wisconsin-Madison, Madison, WI 53706, USA; School of Pharmacy, University of Wisconsin-Madison, Madison, WI 53705, USA; Department of Chemistry, University of Wisconsin-Madison, Madison, WI 53706, USA; McArdle Laboratory for Cancer Research, University of Wisconsin-Madison, Madison, WI 53706, USA; UW Carbone Cancer Center, School of Medicine and Public Health, University of Wisconsin-Madison, Madison, WI 53705, USA

## Abstract

CTR9 is the scaffold subunit in polymerase-associated factor complex (PAFc), a multifunctional complex employed in multiple steps of RNA Polymerase II (RNAPII)-mediated transcription. CTR9/PAFc is well known as an evolutionarily conserved elongation factor that regulates gene activation via coupling with histone modifications enzymes. However, little is known about its function to restrain repressive histone markers. Using inducible and stable CTR9 knockdown breast cancer cell lines, we discovered that the H3K27me3 levels are strictly controlled by CTR9. Quantitative profiling of histone modifications revealed a striking increase of H3K27me3 levels upon loss of CTR9. Moreover, loss of CTR9 leads to genome-wide expansion of H3K27me3, as well as increased recruitment of PRC2 on chromatin, which can be reversed by CTR9 restoration. Further, CTR9 depletion triggers a PRC2 subtype switch from the less active PRC2.2, to the more active PRC2.1 with higher methyltransferase activity. As a consequence, CTR9 depletion generates vulnerability that renders breast cancer cells hypersensitive to PRC2 inhibitors. Our findings that CTR9 demarcates PRC2-mediated H3K27me3 levels and genomic distribution provide a unique mechanism that explains the transition from transcriptionally active chromatin states to repressive chromatin states and sheds light on the biological functions of CTR9 in development and cancer.

## INTRODUCTION

The polymerase-associated factor complex (PAFc) was originally identified as a RNA Polymerase II (RNAPII)-interacting complex in *Saccharomyces cerevisiae* over 20 years ago. PAFc has emerged as a highly conserved complex regulating multiple steps of RNA polymerase II (RNAPII)-mediated transcription (1). In budding yeast, PAFc consists of five subunits, including PAF1, CTR9, CDC73, LEO1 and RTF1. In higher eukaryotic organisms, RTF1 is loosely attached to PAFc, and SKI8/WDR61 is a new subunit of PAFc (2). PAFc regulates multiple phases of transcription, including transcription elongation, transcription termination and RNA 3′-end polyadenylation (1). Recent studies show that PAF1 and CTR9 are essential for PAFc integrity (3–5). PAFc promotes RNAPII pause release (5) and regulates gene expression by controlling multiple transcription coupled histone modifications, including H2BK123ub, H3K4me2/3 and H3K36me2/3, via interaction with different histone modification enzymes (6). The defined functions of PAFc in chromatin modification and transcription elongation control have marked PAFc as an essential regulator of RNAPII-mediated transcription. Inactivation or overexpression of different subunits in PAFc has been found in various cancer types, and the oncogenic or tumor suppressor function of individual subunits appears to be context-dependent (7). For example, germline mutations in *CTR9* were identified in Wilms tumor families, implicating *CTR9* as a Wilms tumor predisposition gene (8). We have found that CTR9 is enriched in estrogen receptor α (ERα) positive breast cancers, and high expression of CTR9 correlates with poor prognosis and tamoxifen resistance (9). Knocking down CTR9 in ERα+ breast cancer cells erased >90% of estrogen-regulated transcriptional response, demonstrating CTR9’s function in promoting breast cancer progression (10).

Polycomb repressive complex 2 (PRC2), the sole mammalian multi-subunit complex responsible for H3K27me3, is essential for maintaining cellular identity and development of multicellular organisms (11). PRC2 is comprised of the core subunits EZH1/2, EED, SUZ12 and RbAp46/48, as well as several sub-stoichiometric proteins (12,13). Though EZH2 is the catalytic subunit of PRC2, the physical interaction of EZH2 with EED and SUZ12 is necessary for full H3K27 methylation activity, as well as for modulating complex stability, and nucleosome binding ability. In the absence of EED or SUZ12, EZH2 is autoinhibited (14–16). Distinct stimulatory models of PRC2 including allosteric activation by its own catalysis (17) as well as auto-methylation of EZH2 at several lysine residues (18) have been reported. Emerging evidence supports the existence of two different PRC2 complex subtypes in vertebrate. The subtype is determined by the auxiliary proteins associated with the core PRC2 complex (11,12). EPOP or PALI and one of the Polycomb-like (PCL) proteins (either MTF2, PHF1 or PHF19) are found in PRC2.1, while AEBP2 and JARID2 are found in PRC2.2 (19). The auxiliary proteins are known to either promote PRC2 activity, facilitate its stability on chromatin or both. For example, JARID2, a PRC2.2-specific subunit, is methylated by EZH2, and methylated JARID2 mimics the methylated H3 tail to stimulate PRC2 activity (20). MTF2, a protein that forms the PRC2.1 sub-complex, is required for sufficient recruitment of PRC2 to CpG islands in mouse embryonic stem cells (mESCs) (21). Although PRC2.1 seems to have higher enzymatic capacity to catalyze H3K27me3 propagation accross the genome than PRC2.2 during the *de novo* establishment of H3K27me2/3 repressive domains (22), the mechanisms of PRC2 recruitment to CpG islands in humans, especially in the context of cancer, remain unclear (11). The overall H3K27me3 levels are thought to be deliberately balanced by PRC2.1 and PRC2.2, to achieve an optimized H3K27me3 concentration either at nucleation or proximal/distal spreading sites (23).

The mutations in PRC2 members are frequently found in human cancers, which are often accompanied by the alteration of global levels of H3K27me2/3 (24). Elevated EZH2 levels in breast cancer are associated with poor prognosis. Pharmacological inhibition of EZH2 is under intensive investigation for combating cancers with aberrant PRC2 activity (25). Tazemetostat, an EZH2 inhibitor, was recently approved by the FDA for the treatment of adult patients with relapsed follicular lymphoma (26). Single-cell analysis showed that loss of H3K27me3 was associated with treatment-resistant breast cancer (27), highlighting the need to further understand how chromatin states affect drug sensitivity.

This study uncovers that H3K27me3 levels are precisely controlled by CTR9 levels in breast cancer cells. Global quantitative analyses of histone modifications reveal a significant increase in genome-wide H3K27me3 levels upon depletion of CTR9. This is partially attributed to the moderate increase of PRC2 recruitment, but more significantly, to the switch from PRC2.2 to PRC2.1, of which PRC2.1 has stronger H3K27me3 methylation activity and propagation capability when transcription is ablated by CTR9 depletion. Genes harboring CTR9 binding sites are often found to gain H3K27me3 repressive signals upon loss of CTR9. Finally, CTR9-depleted cells become addicted to H3K27me3 and are hypersensitive to PRC2 inhibition. Collectively, our study uncovers a unique mechanism by which a transcriptional elongation factor demarcates the PRC2-mediated H3K27me3 domains in breast cancer cells and provides a molecular basis for therapeutically targeting CTR9-low breast tumors with EZH2 inhibitors.

## MATERIALS AND METHODS

### Cell lines and cell culture

HEK293T, MCF7 and T47D cell lines were obtained from the American Type Culture Collection (ATCC) and maintained in Dulbecco’s modified medium (DMEM) (Gibco) supplemented with 10% fetal bovine serum (FBS) (VWR) and 1% Penicillin-Streptomycin (P/S) (Gibco). BT474 cell lines were obtained from ATCC and maintained in RPMI 1640 medium (Gibco) supplemented with 10% FBS and 1% P/S. MCF7-tet-on-parental cells and MCF7-tet-on-shCTR9 cells were generated previously (9) and maintained in DMEM supplemented with 10% FBS and 1% P/S. All cells were cultured at 37°C and 5% CO_2_ in a humidified incubator.

### Generation of the MCF7-3xFLAG-KI-CTR9 cell line

Guide RNAs (gRNAs) targeting Exon1 of CTR9 were prepared by mixing CRISPR RNA (crRNA) and trans-activating CRISPR RNA (tracrRNA) at equimolar concentrations in a sterile microcentrifuge tube for a final duplex concentration of 100 μM. RNAs were heated at 95ºC for 5 min and allowed to cool to room temperature (15–25ºC) on the bench top. Ribonucleoprotein particles (RNP) were prepared by mixing Cas9 enzyme and gRNAs targeting Exon1 of CTR9 (Supplementary Table S1) and incubating at room temperature for 10 min. MCF7 cells were transfected with RNPs and 3xFLAG ssDNA (Supplementary Table S1) using the Lonza 4D-Nucleofector system. Single cells were plated in 96-well plates. Following single colony formation, genomic DNA from each clone was extracted, and PCR was performed to detect whether a 3xFLAG tag was knocked-in at the N-terminus of *CTR9*. The PCR products were also sequenced using Sanger sequencing. To further confirm that a 3xFLAG tag was successfully knocked-in, protein was extracted from MCF7-FLAG-KI-CTR9 cells and MCF7 parental cells, and IP was performed using a FLAG antibody. The results showed that CTR9, as well as other PAF complex components, could be pulled down in MCF7-3xFLAG-KI-CTR9 cells, but not in the parental cells.

### 3D spheroid formation

A total of 100 000–300 000 cells of MCF7-shControl/shCTR9#3/shCTR9#5 were fully resuspended in 200–250 μl of DMEM media and seeded in a 96-well round bottom ultralow attachment plate (Corning, Product# 4515), followed by centrifugation at 500 × *g* for 10 min. 3D spheroid cultures were grown at 37°C and 5% CO_2_ for up to 4 days in a humidified incubator.

### Virus packaging, infection and preparation of stable knockdown cell line

Stable knockdown cell lines were generated by lentivirus infection. The viral packaging vectors pME-VSVG and psPAX2 were purchased from Open Biosystems. For lentiviral packaging, 4 μg pME-VSVG, 4 μg psPAX2 and 8 μg of lentiviral shRNA expression vectors were co-transfected into HEK293T cells cultured in one 10 cm dish using TransIT-LT1 reagent (Mirus Bio) according to the manufacturer’s protocol. Medium was replaced with fresh DMEM supplemented with 10% FBS and 1%P/S 8–10 h post transfection. Forty-eight hours after transfection, the supernatant containing viral particles were collected by centrifugation (1500 rpm, 5 min) and subsequently filtered through a 0.45 μm syringe filter (Thermo Scientific). Approximately 1/5 volume of Lenti-X concentrator (Clonetech) was added to concentrate the virus titer overnight at 4°C. For infection, 1 ml of virus was mixed with 1 ml of fresh cell culture medium, and polybrene was added at a final concentration of 8 μg/ml to increase the infection efficiency. After overnight infection, the culture medium was changed. Cells were infected overnight, followed by changing of culture medium. Cells were selected with 2 μg/ml puromycin for at least one week to generate stable cell lines.

### Preparation of chromatin fractions

Cells were harvested after trypsinization. After washing with 1× PBS, two to five volumes of lysis buffer were added to the cell pellet [10 mM HEPES, pH 7.4, 10 mM KCl and 0.05% NP-40, 1× protease inhibitor cocktail (Sigma-Aldrich), phosphatase inhibitor (1 mM NaVO_4_) and deacetylase (5 mM TSA, Sigma-Aldrich)], and then incubated on ice for 20 min. Nuclei pellets were separated by centrifugation at 14 000 rpm at 4°C for 10 min. Subsequently, nuclei pellets were washed once with lysis buffer, resuspended in two to five volumes of low salt buffer [10 mM Tris-HCl, pH 7.4, 0.2 mM MgCl_2_, 1× protease inhibitor cocktail, phosphatase inhibitor (1 mM NaVO_4_), deacetylase (5 mM TSA) and 1% Triton X-100], and incubated on ice for 15 min. Chromatin fractions were separated by centrifugation at 14 000 rpm at 4°C for 10 min, resuspended with two to five volumes of 0.2 N HCl and incubated on ice for 20 min. After centrifugation, the supernatant containing chromatin-associated proteins was neutralized with an equal volume of 1 M Tris-HCl pH 8.0.

### Histone extraction and purification

MCF7/BT474/T47D cells were harvested after trypsinization. After washing with 1× PBS, cell pellets were resuspended in two volumes of lysis buffer [50 mM Tris-HCl, pH 7.4, 150 mM NaCl,10% glycerol, and 0.05% NP-40]. Protease Inhibitors (1× protease inhibitor cocktail) and an HDAC inhibitor (10 mM sodium butyrate) were added before use. The cell pellets were incubated on ice for 30 min, followed by a brief sonication. After 15 min of centrifugation at 13 000 rpm at 4°C, the supernatant was saved as whole cell lysate and the pellet was used for histone extraction. Pellets were washed twice using NIB buffer [10 mM Tris-HCl, pH 7.5, 2 mM MgCl_2_, 3 mM CaCl_2_ and 1% NP-40] containing 100 mM NaCl, and once with NIB buffer containing 400 mM NaCl. The pellet was then resuspended in NIB buffer (400 mM NaCl) without NP-40. For acid extraction of histones, two volumes of 0.2 N HCl were added and incubated overnight at 4°C. After centrifugation at 13 000 rpm for 15 min at 4°C, solubilized histones in the supernatant were dialyzed in ddH_2_O overnight at 4°C using 10K MWCO Dialysis Tubing (Thermo). Histones were lyophilized and dissolved in ddH_2_O.

### Liquid chromatography and quantitative histone mass spectrometry (LC-MS/MS)

#### Chemical derivatization of histones and tryptic digestion

About 25 μg of purified histone was dissolved in 100 μl 100 mM TEAB buffer (pH 8.0). About 4 μl 4% ^13^CD_2_O (w/v) and 4 μl 600 mM NaBD_3_CN were added to the samples, and vortexed at room temperature for 1 h to label the free and mono-methylated lysine with heavy isotopic methyl. The reaction was terminated by acidifying the sample with TFA. The samples were then transferred to a 10K MWCO ultracentrifuge tube (Millipore) and centrifuged for 15 min at 14 000 *g* at 4°C to remove the reaction reagents. The samples were washed twice by adding 200 μl 100 mM TEAB buffer and centrifuging the samples for 15 min at 14 000 *g* at 4°C. About 100 μl 100 mM TEAB buffer and 1 μg trypsin were added to the ultracentrifuge tube, and the samples were incubated at 37°C for 16 h. The digested samples were collected by centrifugation for 15 min at 14 000 *g* at 4°C. The samples were washed twice with 100 μl 100 mM TEAB buffer, and the flow-through and digested samples were combined and dried down. The dried down samples were resolved with 100 μl 50 mM TEAB buffer, and 15 μl 25% propionic anhydride buffer (v/v in ACN), as well as 10–15 μl 28% NH_4_OH, were added to maintain a pH of ∼8.0. The samples were vortexed for 20 min to label the N-termini of the digested histone peptides with propionyl. After propionylation, the samples were desalted with Sep-Pak cartridges (Waters) and lyophilized.

#### LC-MS/MS for histone modification

Lyophilized histone peptides were resuspended in 0.1% formic acid (FA) and analyzed on a Dionex U3000 ultra performance liquid chromatography system coupled to a Q-Exactive HF quadrupole orbitrap mass spectrometer (Thermo Fisher Scientific). A Waters BEH 300Å C18 reversed phase capillary column (150 mm × 75 μm, 1.7 μm) was used for separation. Water with 0.1% FA and acetonitrile with 0.1% FA were used as mobile phases A and B, respectively. The flow rate was set to 0.300 μl/min. About 2 μl of peptide sample was injected onto the column and separated over a 120-min gradient as follows: 0–1 min 3–10% B; 1–90 min 10–35% B; 90–92 min 35–95% B; 92–102 min 95% B; 102–105 min 95–3% B; 105–120 min 3% B. The data were acquired under data dependent acquisition mode (DDA, top 20). Mass spectrometric conditions were as follows: spray voltage of 2.8 kV, no sheath and auxiliary gas flow; heated capillary temperature of 275°C, normalized high-energy collision dissociation (HCD) collision energy of 33%, resolution of 120 000 for full scan, resolution of 60 000 for MS/MS scan, automatic gain control of 2e5, maximum ion injection time of 100 ms, isolation window of 1.6 and fixed first mass of 110 *m*/*z*.

#### Data analysis and relative quantification of histone PTMs

Because histones possess multiple post-translational modifications, and heavy isotopic di-methylation and propionylation further modify these histones, several isoforms for the same peptide sequence with different modifications exist, making it challenging to identify all the different histone modification peptides. Here in this work, the most frequently observed modifications, including mono-, di- and tri-methylation, as well as acetylation on lysine residues of histone H3, were analyzed. The same histone peptides possessing different modifications can be distinguished according to their difference in mass over charge (*m*/*z*). Then the peptides can be additionally distinguished according to their retention time difference on the RP-HPLC column (trimethylated peptide ≈ unmodified, possesses two heavy isotopic methyl. Mono possesses one heavy isotopic methyl, and di-methylated peptide < acetylated peptides). To relatively quantify the abundance of histone PTMs, we used the area of each identified peptide peak in the MS chromatogram for comparison. Normally same peptide may have different charge state ions in LC-MS analysis. We only choose the highest intensity ions to measure their peak area. The total peak area of a histone peptide with all different PTM forms is regarded as 100%, and the percentage of each PTM on the peptide is calculated by dividing the area of the PTM peak area by the total peak area. To further distinguish histone peptide isoforms, we also investigated the MS/MS spectrum to calculate the ratios of *b* and/or *y* ions that were different between two or more peptide isoforms, and the ratio is used to continually calculate the relative quantity of the peptide isoforms.

### Preparation of nuclear lysates

Nuclear lysates were prepared as described (28) and used for co-immunoprecipitation and peptide pulldown experiments. Briefly, cells were harvested in ice-cold PBS and extracted at 4°C in buffer containing 50 mM Tris-HCl, pH 7.5, 5 mM EDTA, 250 mM NaCl and 0.1% NP-40 supplemented with protease and phosphatase inhibitors, for 30 min. After centrifugation at 13 000 rpm at 4°C for 1 h, the supernatant was collected, and mixed with two volumes of 50 mM Tris-HCl, pH 7.5, 5 mM EDTA, 100 mM NaCl, 0.1% NP-40 and 10% glycerol.

### Co-immunoprecipitation using nuclear extracts

Co-IP was performed as previously described (29). Briefly, immunoprecipitation was performed in IP buffer [50 mM Tris-HCl, pH 7.5, 150 mM NaCl, 2 mM MgCl_2_, 0.5% NP-40 and 10% glycerol] supplemented with protease and phosphatase inhibitors before use. Approximately 1.5–2 mg nuclear protein extract, as quantified by a Bradford assay, was mixed with 5 μg of antibody and 50 μl of protein A magnetic Dynabeads (Invitrogen, washed 3 times in IP buffer before incubation) per IP reaction for a total volume of 750 μl. Beads were washed three times with IP buffer, and once with PBST [PBS + 0.5% Tween-20] the following day. Proteins were eluted in 75 μl 2× SDS loading buffer with 50 nM DTT and incubated at 95°C for 15 min before loading onto an SDS-PAGE gel.

### Peptide pulldown using nuclear extract

Peptide pulldown was adapted from published protocol (29). Lysine methylated peptide with a C-terminal biotin tag ATKAAR-Kme3-SAPSTGGVKKPHRYRPG-GGK(Biotin)-NH_2_ was synthesized by Active Motif^®^. For each peptide pulldown, 50 μg of magnetic streptavidin beads (Medchem Express) were incubated with 5 μg of peptide in 500 μl of binding buffer [50 mM Tris-HCl pH7.5, 150 mM NaCl, 1 mM EDTA and 1% NP-40] for 3 h, rotating at room temperature. Peptide bound beads were washed three times in binding buffer followed by incubation with ∼ 500 μg nuclear extracts (pre-cleared with blank streptavidin beads) at 4°C overnight. Discard the supernatant the following day and the streptavidin beads was sequentialy washed three times in wash buffer I [50 mM Tris-HCl, pH 7.5, 150 mM NaCl, 1 mM EDTA and 0.05% Triton X-100], and twice in wash buffer II [50 mM Tris-HCl, pH 7.5, 150 mM NaCl and 1 mM EDTA]. Pulled-down proteins were eluted by vibrating the beads on a vortexer twice in 50 μl U/T buffer [6 M urea, 2 M thiourea, 150 mM NaCl, 30 mM biotin in 10 mM HEPES, pH 8.0] at room temperature for 10 min and at 95°C for 15 min. The two eluates were combined for western blot analysis.

### *In vitro* histone methyltransferase assay (HMT) using nuclear extract as the enzyme source

About 10 μg of biotinylated histones or poly-nucleosomes were bound to pre-equilibrated streptavidin beads for 1 h at room temperature. The beads were washed with reaction buffer [50 mM Tris-HCl, pH 8.6, 0.02% Triton X-100, 2 mM MgCl_2_, 1 mM TCEP] twice after incubation. Pre-bound biotinylated histones or poly-nucleosomes were equally aliquoted to each HMT reaction. The nuclear extract was pre-cleared with 1/500 volume of blank streptavidin beads, followed by buffer exchange to reaction buffer using 3 KDa Ultra centrifuge filter. The concentration of the pre-cleared nuclear extract was then measured by Bradford assay. Ascending amounts of pre-cleared nuclear extracts were incorporated in the HMT reactions, supplemented with 100 nM ATP and 100 μM S-adenosyl methionine. The reaction mixture was incubated at 37°C for 1 h. The supernatant was discarded, and the streptavidin beads were sequentially washed with 2 times of reaction buffer and 3 times of strong wash buffer [2 M Urea in 10 mM Tris‐HCl pH 8.0]. Biotinylated histones or poly-nucleosome were eluted with an equal volume of 2× SDS loading buffer with 50 nM DTT and heated at 95°C for 15 min before loading on an 12% SDS-PAGE gel.

### Glycerol gradient sedimentation

To prepare a glycerol gradient in a 5 ml polyallomer tubes (Beckman), pre-filtered K150 buffer [50 mM HEPES pH7.9, 1 mM EGTA, pH 8.0, 2 mM MgCl_2_, 600 mM KCl, and freshly added protease inhibitors] containing 30–50% glycerol (v/v) was layered from bottom to top. Freshly prepared nuclear extracts were concentrated and resuspended in K150 buffer without glycerol. After Bradford quantification, about 150–200 μg (<200 μl) of nuclear extract was gently layered on the top of the glycerol gradient. The 5 ml polyallomer tubes were inserted in a pre-chilled SW28 rotor and ultracentrifuged at 25 000 × *g* for 14.5 h at 4°C (Beckman Optima XPN). The glycerol gradient was fractionized by repeatedly removing 200 μl from the top to the bottom without disturbing the tube. One volume of 2× SDS loading dye was added to each fraction before SDS-PAGE analysis.

### Western blotting and Ponceau S staining

Cells were harvested after trypsinization, washed with 1× Dulbecco’s phosphate buffer saline (DPBS) (Life Technologies) and lysed in lysis buffer [50 mM Tris-HCl pH 8.0, 400 mM NaCl, 10% glycerol, 0.5% Triton X-100 and 1× protease inhibitor cocktail (Sigma-Aldrich)]. After a brief sonication, total lysate was centrifuged, and the supernatant was quantified using the BioRad Protein Assay (BioRad). Approximately 30 μg protein was resolved by SDS-PAGE. Proteins were transferred to a nitrocellulose membrane for 1.5 h at 350 mA. Membranes were blocked with 5% nonfat milk or 5% BSA at room temperature for 1 h and incubated overnight with diluted primary antibody at 4°C. Membranes were then washed and incubated with HRP-conjugated goat-anti-rabbit or mouse IgG secondary antibody for 1 h at room temperature. Membranes were incubated with enhanced chemiluminescence reagents (Thermo Scientific) followed by exposure to X-ray films.

For Ponceau S staining of histone extractions, the transferred membranes were first briefly washed with ddH_2_O. The membranes were then stained with Ponceau S and put on a shaker for 20 min at room temperature. Finally, the membranes were washed with de-staining buffer [2% acetic acid].

### ELISA assays for quantification of histone modifications

ELISA measurement of specific Histone H3 modifications was performed using the Histone H3 Modification Multiplex Assay Kit (abcam) according to manufacturer’s protocol. In brief, extracted histone mixture in Antibody Buffer (provided in the kit) was aliquoted to wells coated with specific Histone H3 modification antibodies. After incubation at 37°C for 2 h, the solution in the wells was removed, and wells were washed 3× in wash buffer (provided in kit). Diluted detection antibody solution (provided in the kit) was added and incubated at room temperature for 1 h. After two brief washes in wash buffer, developer solution was added followed by incubation at room temperature for 2–10 min, until wells sufficiently turned blue, while blank wells with no histone extract added remained transparent. The reaction was stopped using stop solution (provided in the kit). The absorbance of each well was read on a microplate reader at 450 nm with an optional reference wavelength of 655 nm. Background absorbance was subtracted, as measured by the blank wells, and the readings were subsequently normalized by the corresponding absorbance in the control wells coated with H3-total antibody.

### Cell proliferation and cell cycle analyses

For cell counting-based proliferation assays, 1 × 10^5^ cells were seeded into six 3.5 cm dishes for compound treatment. MCF7-tet-on–shCtr9 cells were pretreated with vehicle or 500 ng/ml Dox for 5 days before seeding to 3.5 cm Petri dishes. Media were changed every 48 h. Cells were trypsinized and counted after trypan blue exclusion using an automated cell counter (Bio-Rad).

For 3-(4,5-dimethylthiazol-2-yl)-2,5-diphenyltetrazolium (MTT)-based (Sigma-Aldrich) proliferation assays, 2 × 10^3^ cells were seeded into 96-well plates for compound treatment. About 15 μl MTT (5 mg/ml in DPBS) was added to the cells followed by incubation at 37°C for 1 h. After removing cell culture medium, 50 μl of DMSO was added. The absorbance was measured with a 540-nm filter on a VictorX5 microplate reader (Perkin Elmer), and data were plotted and analyzed using GraphPad Prism 8 software (GraphPad Software, Inc.).

Edu staining-based cell proliferation analysis was performed by using the Click-iT™ Plus Edu Flow Cytometry Assay Kit (Invitrogen) according to manufacturer’s protocol. In brief, cells were labeled with 10 μM Edu staining buffer in culture medium for 2 h. After a brief wash with 1% BSA in DPBS, cells were harvested by centrifugation. Cells were then fixed in fixative buffer (provided in the kit) at room temperature for 15 min. After washing with 1% BSA in DPBS twice, cells were resuspended in saponin-based permeabilization buffer (provided in the kit) at room temperature for 15 min. About 500 μl Click-iT™ Plus reaction cocktail was added to each sample, followed by incubation of the reaction mixture in the dark at room temperature for 30 min. Cells were washed with permeabilization buffer, wash buffer (provided in the kit), and then subjected to flow cytometry analysis.

### Annexin V and PI staining

A total of 1–5 × 10^5^ trypsinizied cells were collected by centrifugation. Cells were washed with 1× cold PBS, and the supernatant was carefully removed. The cells were resuspended in binding buffer [10 mM HEPES, pH 7.4, 140 mM NaCl and 2.5 mM CaCl_2_] at a concentration of ∼1 × 10^6^ cells/ml. After brief centrifugation, cells were resuspended and incubated for 10 min with 0.5 μg/ml Annexin V-FITC and 2 μg/ml PI in 400 μl binding buffer. The cells were immediately placed on ice and analyzed by flow cytometry. Cell fragments were removed by morphological gating. Cells negative for annexin V-FITC and PI were considered viable, annexin V-FITC positive and PI negative cells were considered apoptotic, and annexin V-FITC positive and PI positive cells were considered necrotic.

### Cytotoxicity assay of 3D spheroids

The cytotoxicity assay was performed according to manufacturer’s instructions (Invitrogen, Cat# L3224). In brief, calcein AM, ethidium homodimer-1, as well as 1 μM Hoechest 33342 were added to DMEM and incubated with spheroids at 37°C for 30 min. After incubation, the spheroids were immediately subjected to confocal imaging (Nikon W1 confocal) in a live cell incubation chamber. The area of the spheroids was measured using NIS-A1R Advanced Research Imaging Software (Nikon - Mager Science) with nuclei annotation.

### Immunofluorescence staining of H3K27me3

Cells were seeded on 3.5 cm dishes with glass bottom before fixation in 4% formaldehyde for 15 min and then washed with DPBS three times. Subsequently, cells were permeabilized in 0.3% Triton X-100 in PBS for 10 min, blocked with 3% BSA in PBST [PBS + 0.1% Triton X-100] for 1 h and incubated with H3K27me3 primary antibody (Cell Signaling Technology) at room temperature for 2 h. Cells were then washed with PBST for 3 times, followed by incubation with secondary antibody (Cy5-goat anti-rabbit IgG (H + L), 1:250; Bethyl) for 30 min at room temperature. After being washed 3 times in PBST, cells were incubated with 50 nM Alexa Fluor 555 Phalloidin (Cell Signaling Technology) and 1 μg/ml of Hoechest 33342 (Cell Signaling Technology) at 37°C for 15 min, then washed twice in DPBS. Fluorescence was detected using a Nikon A1R confocal microscope at appropriate wavelengths at the UW imaging core. Signal intensity was analyzed in NIS-A1R Advanced Research Imaging Software (Nikon - Mager Science)

### Immunofluorescence staining of PRC2 subtype proteins

Cells were seeded on 3.5 cm glass bottom dishes, fixed in 4% formaldehyde for 15 min and then washed with DPBS three times. Subsequently, cells were permeabilized in 0.3% Triton X-100 in PBS for 10 min, blocked with 3% BSA in PBST [PBS + 0.1% Triton X-100] for 1 h and incubated with JARID2 primary antibody (Cell Signaling Technology) at room temperature for 3 h. Cells were then washed with PBST for three times, followed by incubation with secondary antibody (Cy3 AffiniPure Fab Fragment Goat Anti-Rabbit IgG (H + L), 1:250; Jackson ImmunoResearch) for 1 h at room temperature. After being washed for three times in PBST, cells were briefly rinsed with blocking buffer. Cells were incubated with MTF2 primary antibody (ProteinTech) diluted in blocking buffer overnight at 4°C. Cells were then washed with PBST, followed by incubation with secondary antibody (Cy5-goat anti-rabbit IgG (H + L), 1:250; Bethyl) for 1 h at room temperature. After being washed three times in PBST, cells were incubated with 1 μg/ml of Hoechest 33342 (Cell Signaling Technology) at 37°C for 15 min, then washed twice in DPBS.

### Quantification of H3K27me3 intensity

The Dox treatment of MCF7-tet-on-shCTR9 cells was performed as previously described above. Cells were harvested after trypsinization, washed in PBS and fixed in 4% paraformaldehyde for 15 min. Subsequently, cells were permeabilized with 0.3% Triton X-100 in PBS for 10 min blocked with 3% BSA in PBST for 1 h and incubated with Cy5 conjugated H3K27me3 antibody (Abcam) in 1% BSA in PBST for 1 h. After washing with PBST, cells were subjected to flow cytometry analysis.

### Quantitative real-time PCR

Total RNA was extracted using E.Z.N.A Total RNA kit (Omega Bio-tek). About 2 μg of RNA was reverse transcribed using Superscript II RT according to the manufacturer’s instructions (ThermoFisher), and quantitative PCR was performed using SYBR Green dye (Roche) and the CFX96 Touch Real-Time PCR Detection System (Bio-Rad). Primer sequences used in this study are listed in DYRAD.

### Chromatin immunoprecipitation with exogenous reference genome (ChIP-Rx)

Cells in 15 cm dishes were washed once with PBS before cross-linking with PBS containing 1% formaldehyde for 15 min at room temperature. Cross-linking was quenched with 0.125 M glycine for 5 min at room temperature before two washes with ice-cold PBS. Cells were scraped, harvested by centrifugation and subjected to ChIP assays. Cross-linked cells were lysed with lysis buffer 1 [10 mM HEPES pH 7.0, 10 mM EDTA, 0.5 mM EGTA, 0.25% Triton X-100, supplemented with 0.5 mM PMSF before use] with rotation at 4°C for 10 min. The crude nuclear pellets were collected by centrifugation at 1500 rpm for 4 min at 4°C. The supernatant was discarded, and the chromatin was washed with lysis buffer 2 [10 mM HEPES, pH 7.0, 200 mM NaCl, 1 mM EDTA, 0.5 mM EGTA, supplemented with 0.5 mM PMSF before use] for 10 min at 4°C with rotation. Nuclear pellets were collected by centrifugation (1500 rpm, 4°C, 4 min), resuspended in nuclear lysis buffer [50 mM Tris-HCl pH 8.1, 10 mM EDTA, 1% SDS, supplemented with 1 mM PMSF and 1× protease inhibitor cocktail (Sigma-Aldrich) before use] and incubated on ice for 10 min. Chromatin was sheared to approximately 100–1000 bp fragments by sonication in an ice-water bath at 4°C using a Branson Sonifier 450 with a microtip (40% amplitude, 3 s on, 10 s off, 5 min total pulse time). Sonicated chromatin was centrifuged at 15 000 rpm for 15 min at 10°C, and the concentration of nuclear proteins was determined using the BioRad Protein Assay (BioRad). Equal amounts of total nuclear proteins were used for ChIP. Nuclear proteins were supplemented with nuclear lysis buffer to achieve equal volumes for different samples and then diluted 1:10 with dilution buffer [20 mM Tris-HCl, pH 8.1, 150 mM NaCl, 2 mM EDTA, 1% Triton X-100, supplemented with 1× protease inhibitor cocktail before use]. About 20 μg of sheared *Drosophila* chromatin derived from S2 cells plus 2 μg H2A.V antibody were supplied. Five percent of the chromatin fraction was removed and saved as input, and the rest was pre-cleared with a normal IgG control before incubating with the antibody of interest overnight at 4°C.

The following day, immune complexes were incubated with Dynabeads™ Protein A/G or Dynabeads™ M-280 Sheep anti-Mouse IgG (Life Technologies) (beads were pre-washed with ChIP dilution buffer three times before use) while rotating at 4°C for 2 h. The immunoprecipitated materials were subsequently washed once with low salt wash buffer [20 mM Tris-HCl, pH 8.1, 150 mM NaCl, 2 mM EDTA, 0.1% SDS, 1% Triton X-100], once with high salt wash buffer [20 mM Tris-HCl, pH 8.1, 500 mM NaCl, 2 mM EDTA, 0.1% SDS, 1% Triton X-100], once with LiCl wash buffer [10 mM Tris-HCl, pH 8.1, 0.25 M LiCl, 1 mM EDTA, 1% NP-40, 1% deoxycholate] and twice with TE buffer [10 mM Tris-HCl, pH 8.0, 1 mM EDTA pH 8.0]. Each wash was done with rotation at 4°C for 5 min followed by separation on a magnetic stand. The immunoprecipitated material was eluted twice in freshly prepared elution buffer [1% SDS, 0.1 M NaHCO_3_] with shaking on a vortexer for 20 min at room temperature. The eluted and input materials were then digested with proteinase K (200 μg/ml final concentration) at 55°C for 2 h. The cross-linking materials were reversed by incubating at 65°C in a hybridization oven overnight. DNA was purified using a Qiagen PCR Purification Kit per manufacturer’s protocol.

### ChIP-seq library preparation

Prior to ChIP-seq library preparation, the concentration and size distribution of the ChIPed DNA samples was determined using a Qubit Fluorometer (Thermo Fisher Scientific) and an Agilent High Sensitivity DNA Kit (Agilent Technologies), respectively at the Sequencing Facility at Northwestern University (NUseq core). Approximately 10–100 ng of ChIPed DNA from each condition was used to generate the ChIP-seq library using the Ovation Ultralow System V2 1–16 Kit (NuGEN Technologies), according to the manufacturer’s protocol. Briefly, the DNA was end-repaired and ligated to Illumina sequencing adaptors. The ligated DNA was purified using Agencourt RNAClean XP beads (Beckman Coulter). A subsequent PCR amplification step (8–15 cycles) was performed to add a linker sequence to the purified fragments for annealing to the Genome Analyzer flow-cell. Following PCR amplification, the library was separated on a 2% agarose gel (120 V, 1.5 h) to select a narrow range of fragment sizes, and bands between 200 and 500 bp were excised. The library was purified from the excised agarose gel using the Qiagen MiniElute PCR Purification Kit following the manufacturer’s protocol. Quality control for the size, purity, and concentration of the final ChIP-seq libraries was performed at the Sequencing Facility at University of Wisconsin-Madison Biotechnology Center. Qualified libraries were deep sequenced using an Illumina HiSeq 4000 or NextSeq 500 per the manufacturer’s instructions at Northwestern University (NUseq core).

### Bioinformatic analyses of ChIP-Rx datasets

Trimmed ChIP sequencing reads that passed quality score were aligned to the human reference genome (hg38) using Bowtie2 (v2.4.1) (30). Default output of SAM file were then transformed into BAM format by using samtools (v1.1.3). Only unique alignments that passed high mapping quality (MAPG > 30) were retained for downstream analysis. Duplicated reads and reads residing in hg38 blacklist regions (https://github.com/Boyle-Lab/Blacklist/tree/master/lists) were eliminated by sambamba tools (v0.7.1). Sequencing reads were also aligned to the *Drosophila* genome (dm6) and normalization factors (Rx factor) were calculated after removing multi-mapping and duplicated reads. Bigwig files were generated at a resolution of 20 bp using the bamCoverage utility from the deepTools (v3.4.3) suits (31), and data were subsequently visualized as ChIP-Rx normalized tracks using IGV genome browser (v2.8.9). Wiggletools (v1.2.3) and wigtoBigWig (v377) was used to generate the mean of bigwig files from two biological replicates. Peaks were called using MACS2 (v2.1.4) (32) with FDR < 0.01 for narrow peaks and FDR < 0.05 for broad peaks. Peak intersection and sorting were performed by bedtools (v2.29.2) and bedops (v2.4.40). Overlapping peaks shorter than the sequencing tag were discarded. Average ChIP-Rx signal on particular regions or genes were calculated by multiBamSummary/ multiBigwigSummary utility from the deepTools (v3.4.3) suits. All heatmap and average line-profile plots were made using the plotHeatmap/ plotProfile utility also in deepTools (v3.4.3) suits.

Peaks annotation was performed by the ChIPseeker (v1.26.0) package in R. Peak associated genes were assigned to a nearest peak if it was within the range of a TSS-TES ± 2.5 kb. Pathway enrichment (GO) and Motif analysis were performed using the functional annotation tool in DAVID Bioinformatics Resources (v6.8) and Homer Motif Analysis in Homer software (v4.11). *Cis*-regulatory factors prediction was performed by importing peak associated genes into BART software (v2.0).

### mRNA expression correlation analysis using published breast tumor RNA-seq data from TCGA

For Figure 5C, TCGA Breast Cancer clinical records and RNA-seq datasets were downloaded from cBioPortal (https://www.cbioportal.org) (33). Estrogen receptor (ER) status was determined by the entry ‘breast_carcinoma_estrogen_receptor_status’ in the patient’s clinical record. There are 817 ER positive patients with primary tumor RNA-seq data. RNA-Seq by Expectation Maximization (RSEM) to all samples was used to study gene expression levels. Pearson correlation was calculated between two genes’ log_10_(RSEM). *P*-values were adjusted using the Benjamini and Hochberg procedure to account for multiple hypothesis testing.

### Statistical analysis

Statistical comparisons between two groups for ChIP-Rx data were performed with Graphpad Prism software 8.0 using an unpaired two-tailed *t*-test with a Welch correction or paired two-tailed Student’s *t*-test. The sample size (*n*) is indicated in the figure legends and represents peak or gene numbers. Details for sequence data analyses and statistical significance are described in the specific Materials and Methods section.

## RESULTS

### CTR9 is a determinant of cellular H3K27me3 levels

**Figure 1.**
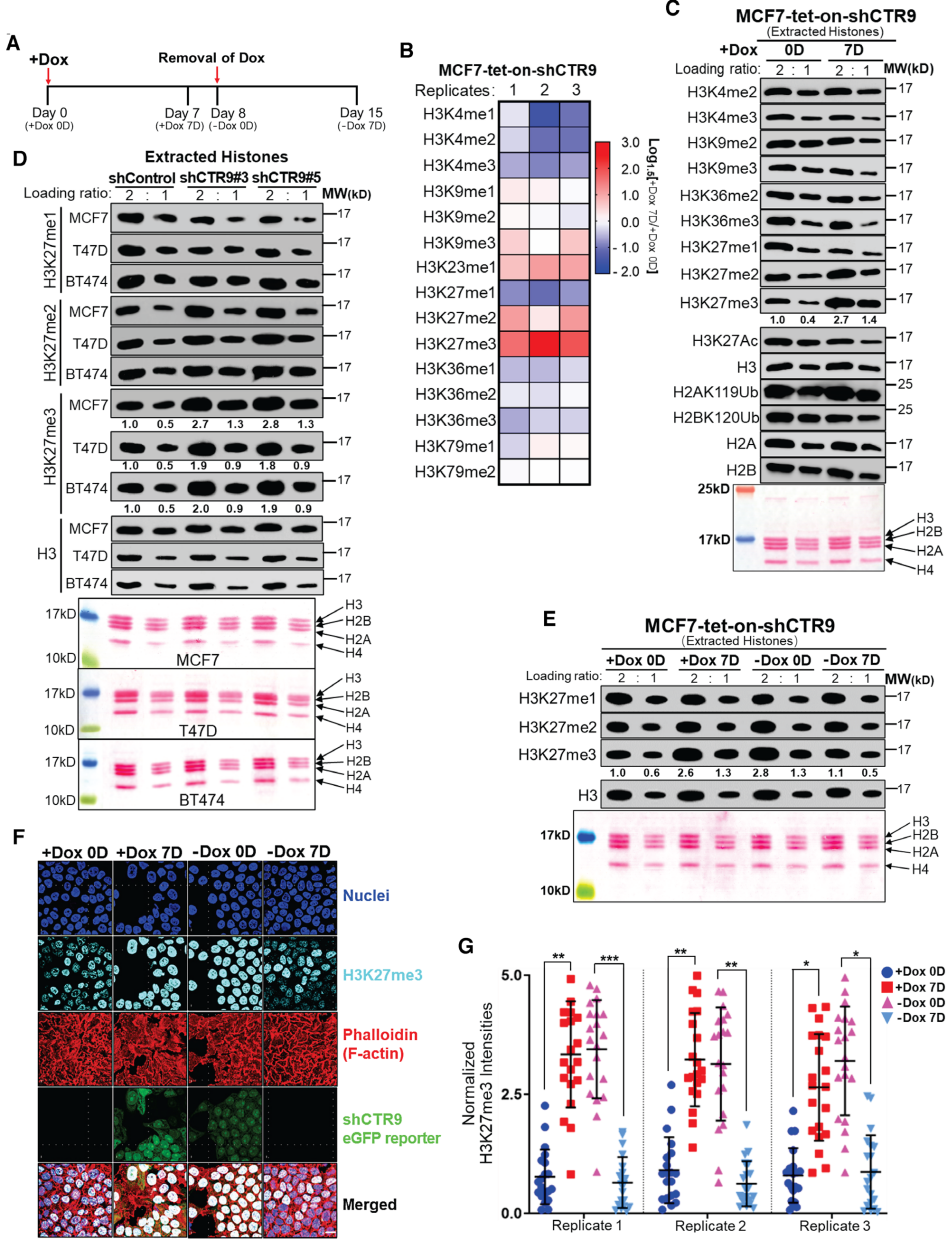
Loss of CTR9 leads to increased H3K27me3 levels in breast cancer cells. (**A**) Schematic workflow of inducible CTR9 knockdown in MCF7 cells by treatment with doxycycline (Dox) for 0 to 7 days and Dox removal for 0 to 7 days. (**B**) Heatmap showing the Log1.5 relative fold-change of histone methylation levels on ‘+Dox 7D (7 days)’ over ‘+Dox 0D (0 day)’ measured by liquid chromatography tandem mass spectrometry (LC-MS/MS) in MCF7-tet-on-shCTR9 cells. (**C**) Western blot analyses of extracted histones after treatment with Dox for 0 day or 7 days in MCF7-tet-on-shCTR9 cells (top). Ponceau S staining of histones (bottom). Each sample is loaded in two-fold dilution. The bands intensity of H3K27me3 were quantified using ImagePro after normalizing with H3 loading controls. (**D**) Western blot analyses of H3K27me1, 2, 3 levels in MCF7 (top), T47D (middle) and BT474 (bottom) cells stably expressing shControl, shCTR9#3, or shCTR9#5. The bands intensities of H3K27me3 were quantified using ImagePro after normalizing with H3 loading controls and shown in two-fold dilution. (**E**) Western Blot analyses of H3K27me1, 2, 3 in MCF7-tet-on–shCTR9 cells under treatment scheme in Figure 1A (top). Ponceau S staining of histones are shown in two-fold loading ratio (bottom). The bands intensity of H3K27me3 were quantified using ImagePro after normalizing with H3 loading controls. (**F**) Representative images of immuno-fluorescence staining of H3K27me3 (cyan), nuclei (blue) and F-actin in MCF7-tet-on–shCTR9 cells under treatment scheme in Figure 1A. 100× scale bar shown at the bottom right applies to all images. (**G**) Ratios of H3K27me3 to nuclei staining intensity in 20 selected cells with complete nuclei. Difference in ratios were significant (*P< 0.05; **P< 0.01; ***P< 0.001) by a two-tailed *t*-test with Welch’s correction; error bars show the standard deviation across triplicate.

CTR9/PAFc has been shown to regulate ERα mediated transcription via coupling multiple histone modifications in breast cancer cells. We sought to achieve a comprehensive understanding on how the global histone modification landscape is altered, as well as whether specific histone modifications are more sensitive to CTR9 depletion than others. To address these questions, we employed a Dox-inducible CTR9 knockdown cell line (i.e., MCF7-tet-on-shCTR9) where shRNA expression can be monitored by expression of eGFP (Figure 1A and Supplementary Figure S1A). Western blotting results showed that treatment with Dox for 7 days (+Dox 7D) was sufficient to deplete CTR9. Beginning on day 8, Dox was removed for 0 day (-Dox 0D) and up to 7 days (-Dox 7D) to allow CTR9 to recover to basal levels (Figure 1A and Supplementary Figure S1A). To measure the levels of histone modifications, total histones were extracted from the nuclear pellets of MCF7-tet-on-shCTR9 cells after treatment with Dox on Day 0 and Day 7, followed by liquid chromatography and tandem mass spectrometry (LC-MS/MS). The transcription-coupled histone modifications such as H3K4me3 and H3K36me3 were decreased by CTR9 depletion, as we reported previously (9). Surprisingly, H3K27me2 and H3K27me3, two transcriptional repressive histone markers, were robustly increased (Figure 1B and Supplementary Table S2). The histone modification changes in response to CTR9 KD were validated by western blotting (Figure 1C) where a significant increase of H3K27me3 was observed after a 7-day Dox treatment. This result suggests that loss of CTR9 results in a global increase of H3K27me3 levels in MCF7 cells. Furthermore, a similar observation was made in MCF7, T47D and BT474 cells stably expressing two distinct CTR9-targeting shRNAs (shCTR9#3 or #5) (Supplementary Figure S1B), as measured by western blotting of purified histones and ELISA assays (Figure 1D and Supplementary Figure S1C). To interrogate whether elevation of H3K27me3 levels in CTR9 KD cells can be reversed by re-expressing CTR9 when Dox was removed, we performed a time-course Dox addition and removal experiment as described above (Figure 1A). Figure 1E shows that H3K27me3 levels increased when CTR9 was depleted, and that H3K27me3 returned to its original levels when CTR9 levels were restored. The dynamic changes of H3K27me3 in response to CTR9 levels were further confirmed by flow cytometry using an Alexa Fluor^®^647-labeled H3K27me3 antibody (Supplementary Figure S1D) as well as an H3K27me3 ELISA assay (Supplementary Figure S1E). To visualize the H3K27me3 changes in individual cells, we performed immuno-fluorescence staining of H3K27me3 using confocal microscopy (Figure 1F). The results showed that, although the response of individual cells varies, possibly due to the heterogeneous expression of shCTR9 (expression indicated by eGFP), the overall intensity of H3K27me3 staining was inversely correlated with CTR9 levels in a dynamic manner (Figure 1G). Together, these data strongly suggest that CTR9 is a *bona fide* regulator of cellular H3K27me3 levels.

### CTR9 confines the intensities and genome-wide distribution of H3K27me3 peaks

PRC2 deposits H3K27me3 marks in spatially defined chromatin domains to repress gene expression. Since CTR9 KD leads to a global increase of H3K27me3 levels, we examined whether CTR9 demarcates the enrichment and distribution of H3K27me3 on chromatin. To quantify the histone modification changes precisely in MCF7-tet-on-shCTR9 cells under the treatment conditions depicted in Figure 1A, we performed chromatin immunoprecipitation followed by high-throughput sequencing with incorporation of a reference exogenous genome (ChIP-Rx) using an antibody against H3K27me3.

**Figure 2.**
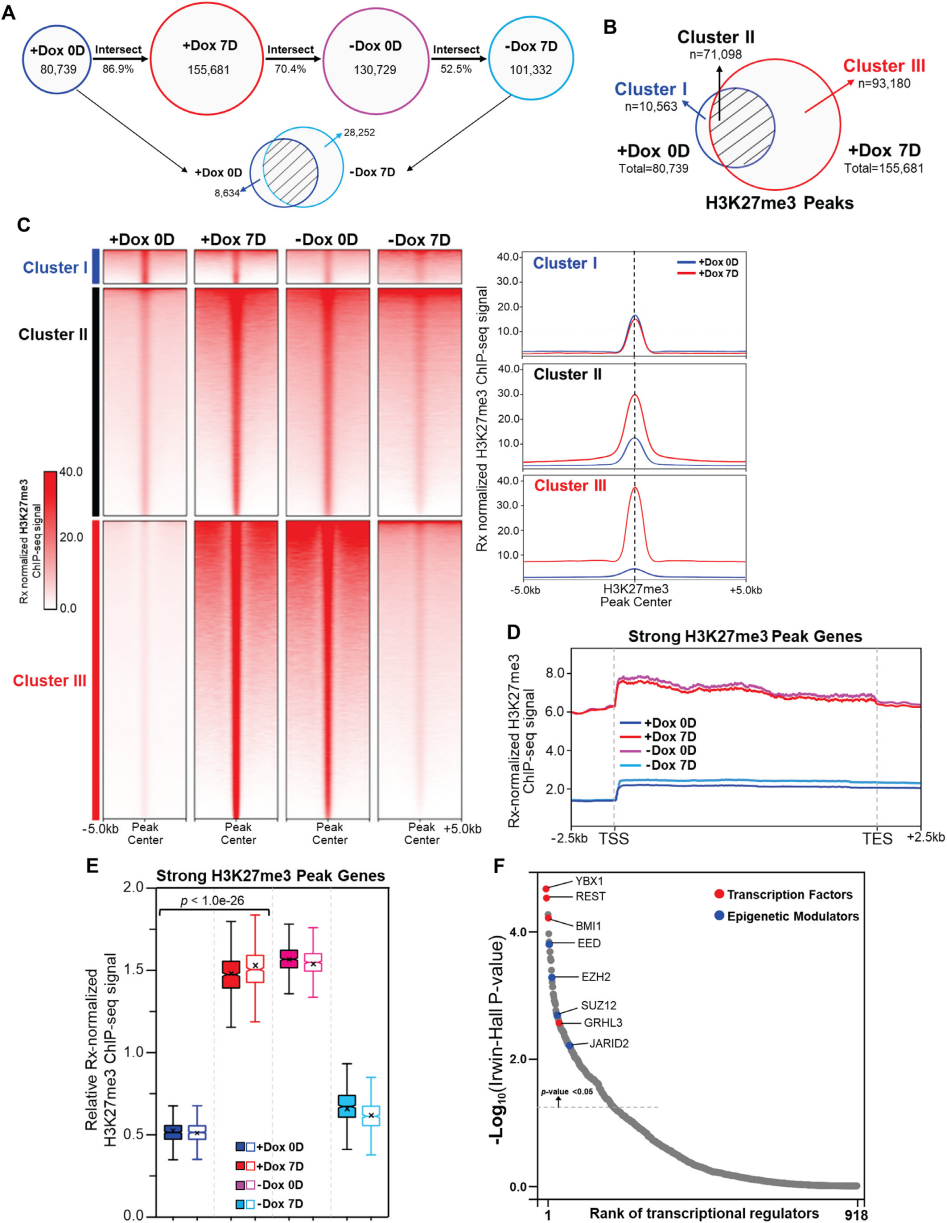
Inducible CTR9 depletion induces a genome-wide gain of H3K27me3 peaks and peak-associated genes, which are reversed by CTR9 re-expression. (**A**) H3K27me3 peak numbers in MCF7-tet-on-shCTR9 cells after addition and removal of Dox for 0 day and 7 days. Peak numbers and percent of intersection between groups are shown. (**B**) Venn Diagram of H3K27me3 peaks between ‘+Dox 0D’ and ‘+Dox 7D’ groups and the designated Clusters. (**C**) Heatmap of Rx-normalized H3K27me3 ChIP-seq profiles at peak center ±5.0 kb regions after addition and removal of Dox for 0 day and 7 days in the designated clusters (left). Composite profile of Rx-normalized H3K27me3 ChIP-seq signals in ±5.0 kb range of peak centers for different clusters (right). (**D**) Average profiles of Rx-normalized H3K27me3 ChIP-seq signals at ±2.5 kb of TSS-TES regions of the strong H3K27me3 peak associated genes. (**E**) Notched boxplot of relative Rx-normalized H3K27me3 ChIP-seq signals of strong H3K27me3 peak genes (*n* = 2). Paired Student’s *t* test was used to calculate statistical significance among groups. (**F**) Ranked dot plot of *cis*-regulatory factor prediction of 11483 strong H3K27me3 peak associated genes by *BART* (Binding Analysis for Regulation of Transcription). The top ranked transcription factors and epigenetic modulators were highlighted in red and blue.

In response to the decrease of CTR9 to a non-detectable level after a 7-day Dox treatment (Supplementary Figure S1A), total H3K27me3 peak numbers increased nearly two-fold (Figure 2A). The H3K27me3 peaks under the ‘-Dox 0D’ condition significantly overlapped with those of the ‘+Dox 7D’ condition (Figure 2A), explained by their similar CTR9 KD status. Notably, the H3K27me3 peak numbers decreased to nearly basal levels in response to CTR9 restoration by removing Dox for another 7 days (-Dox 7D) (Figure 2A). The increased H3K27me3 peaks in response to CTR9 depletion did not display a genome-specific enhancement, but rather, the peaks distributed across broad chromatin regions (i.e., promoter, exon, intron and intergenic regions) (Supplementary Figure S2A). The H3K27me3 ChIP-Rx results demonstrate that the peak numbers of H3K27me3 are inversely and dynamically regulated by CTR9 levels. Next, we interrogated the peak distribution around transcription start sites (TSS) and found that H3K27me3 peaks in ‘+Dox 7D’ condition have a higher frequency to cluster near the TSS or TSS proximal regions as compared to the ‘+Dox 0D’ condition, implying that CTR9 depletion might trigger H3K27me3 nucleation near the TSS (Supplementary Figure S2B). By overlapping H3K27me3 peaks under the basal and CTR9 KD conditions (+Dox 0D versus + Dox 7D), we categorized H3K27me3 peaks into three clusters based on the peak intersection (Figure 2B). Cluster I contains 10 563 H3K27me3 peaks that only exist in the ‘+Dox 0D’ group. Cluster II encompasses overlapping H3K27me3 peaks (71 098) of both groups. Cluster III contains H3K27me3 peaks (93 180) that are unique to the ‘+Dox 7D’ group. It is noteworthy that the number of peaks in Cluster III is significantly higher than in Cluster I. Figure 2C depicts clustered heatmaps (left) and line plots (right) of the average ChIP-Rx signals around the peak center with ± 5.0 kb expansion. Representative snapshots of the genome browser for each cluster are shown in Supplementary Figure S2C. The H3K27me3 peak intensities in cluster I (Supplementary Figure S2C, depicted in blue triangles) are similar between the ‘+Dox 0D’ and ‘+Dox 7D’ groups, even though those peaks are only detected in the ‘+Dox 0D’ group by the peak calling algorithm. In contrast, the intensities of H3K27me3 peaks were significantly increased in cluster II (Supplementary Figure S2C, black triangles) upon CTR9 knockdown (+Dox 7D). In addition, new peaks with robust H3K27me3 ChIP-Rx signal emerged and were classified as cluster III (red triangles). To determine if H3K27me3 peak intensity and peak width are responsive to the dynamic changes in CTR9 levels, we normalized read counts across all three clusters of H3K27me3 peaks in MCF7 CTR9 inducible KD cells. A scatter plot (Supplementary Figure S2D) showed that CTR9 loss had negligible effect on cluster I peaks when comparing the basal (+Dox 0D) and CTR9 KD state (+Dox 7D), indicating that these H3K27me3 peaks represent basal H3K27me3 levels in both conditions. In contrast, cluster II/III peaks were skewed towards the CTR9 KD state (+Dox 7D), revealing a significant increase of H3K27me3 levels in the CTR9 KD state (+Dox 7D) as compared to the basal level (+Dox 0D) (Supplementary Figure S2D). In addition to the two-fold increase of H3K27me3 peak numbers and the increased peak intensities upon CTR9 depletion, the peak widths were broadened (Supplementary Figure S2E). Importantly, these changes were all reversible upon CTR9 recovery. These results demonstrate that loss of CTR9 results in significant expansion of steady state H3K27me3 domains, as well as increased H3K27me3 peak intensities on chromatin, and these changes can be reversed by CTR9 re-expression.

### CTR9-responsive H3K27me3 peak genes are likely regulated by PRC2

Despite the genome-wide enhancement of H3K27me3 upon CTR9 depletion, the tendency of H3K27me3 peaks to cluster in the TSS proximal regions (Supplementary Figure S2B) prompted us to analyze genes putatively regulated by H3K27me3. Because H3K27me3 peaks are in general broad, we assigned each individual H3K27me3 peak merged from ‘+Dox 0D’ and ‘+Dox 7D’ groups to the nearest genes within the range of TSS-TES ± 2.5 kb (34). Among ∼14 000 H3K27me3 peak genes that meet these criteria, 11483 genes displayed at least a two-fold increase in normalized H3K27me3 ChIP-Rx signals after CTR9 knockdown (+Dox 7D/+Dox 0D, Supplementary Figure S2F, Supplementary Table S3). Further examination revealed a significant increase of H3K27me3 ChIP-Rx signals from TSS-2.5 kb to TES+2.5 kb on these genes upon CTR9 loss, as well as a decrease in H3K27me3 ChIP-Rx signals upon CTR9 restoration. We thus classified them as strong H3K27me3 peak associated genes (Figure 2D,E). Notably, over 75% of these genes code for proteins (Supplementary Figure S2G) whose functions are related to general transcriptional regulation, such as transcriptional activator/repressor activity and RNA Polymerase II mediated DNA binding (Supplementary Figure S2H). To deduce their regulation by potential transcription factors (TFs) as well as epigenetic modulators, we performed a *cis*-regulatory factor prediction for the strong H3K27me3 peak associated genes using *BART* tool (Binding Analysis for Regulation of Transcription) (35) (Figure 2F and Supplementary Table S4). Among over 900 TFs and epigenetic modulators, YBX1 (Y-box binding protein 1) and REST (RE1 silencing transcription factor) were the top two predicted TFs, and both have been implicated in breast cancer progression (36,37) Interestingly, several PRC2 subunits including EED, EZH2, SUZ12 and JARID2 were identified among the top hits. These results indicate that CTR9 manifests a strong effect on genes that are regulated by PRC2 complex and H3K27me3.

### Loss of CTR9 results in enhanced PRC2 chromatin association

**Figure 3.**
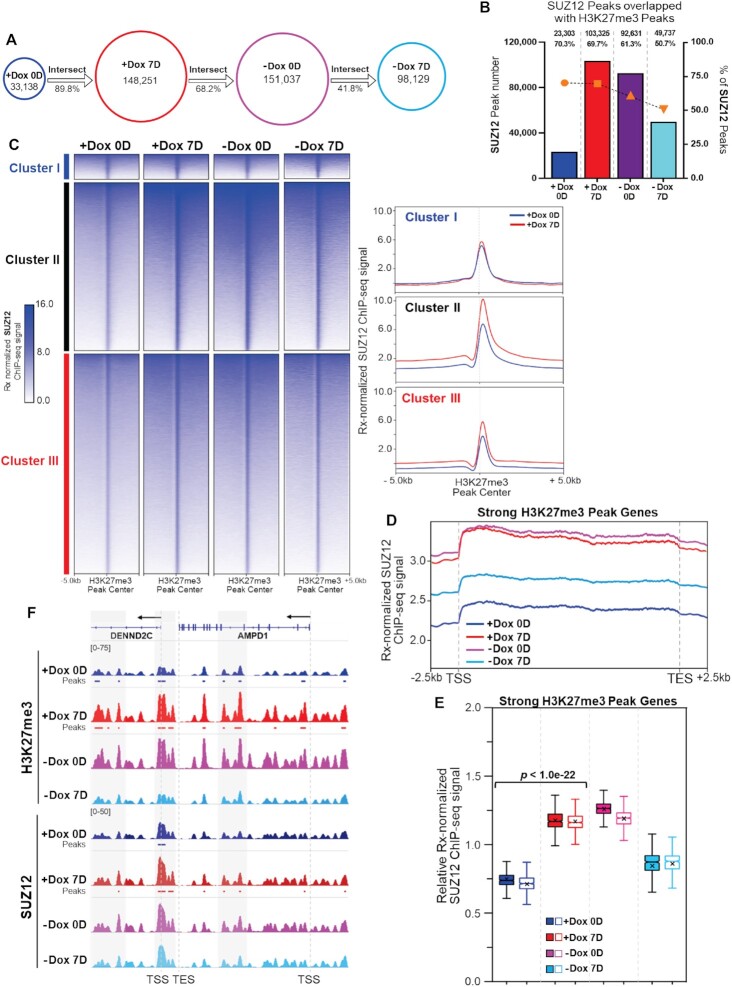
Concomitant genome-wide increase of H3K27me3 and SUZ12 peaks in response to CTR9 depletion. (**A**) SUZ12 peak numbers in MCF7-tet-on-shCTR9 cells under the indicated treatment conditions. (**B**) SUZ12 peak numbers (bar graph, left *Y*-axis) and percentage% (dot-plot, right *Y*-axis) of total SUZ12 peaks that overlapped with H3K27me3 peaks under each treatment condition. Peak numbers were indicated on the top of each bar. (**C**) Heatmap of Rx-normalized SUZ12 ChIP-seq profiles at ±5.0 kb regions after addition and removal of Dox for 0 day and 7 days in the designated clusters (left). Composite profile of Rx-normalized SUZ12 ChIP-seq signals in ± 5.0 kb range of H3K27me3 peak centers for different clusters (right). (**D**) Average profiles of Rx-normalized SUZ12 ChIP-seq signals at ±2.5 kb of TSS-TES regions of 11483 strong H3K27me3 peak genes. (**E**) Notched boxplot of relative Rx-normalized SUZ12 ChIP-seq signals of strong H3K27me3 peak genes (*n* = 2). Paired Student’s *t*-test was used to calculate statistical significance among groups. (**F**) Representative genome-browser snapshot of Rx-normalized H3K27me3 (top) and SUZ12 (bottom) ChIP-seq signals in MCF7-tet-on-shCTR9 cells under treatment conditions indicated. Each signal-track represents the mean of two biological replicates.

H3K27me3 is mainly established by the PRC2-EZH2 complex in human. Given that *cis*-regulatory factor prediction identified several PRC2 subunits at H3K27me3 regulated genes, we examined if CTR9 loss affects PRC2 chromatin recruitment. We performed ChIP-Rx using an antibody against SUZ12, the core subunit of PRC2, in MCF7-tet-on-shCTR9 cells, under the aforementioned treatment conditions (Figure 1A). We found that the peak numbers of SUZ12 increased by 4-fold from ‘+Dox 0D’ to ‘+Dox 7D’ in response to CTR9 depletion (Figure 3A), which resembles the changes of H3K27me3 (Figure 2A). Notably, ∼90% of SUZ12 peaks found in ‘+Dox 0D’ condition were also detected in the ‘+Dox 7D’ group (Supplementary Figure S3A), suggesting that the new PRC2 binding sites may expand from the pre-existing binding sites. As observed with the change of H3K27me3, CTR9 re-expression (-Dox 7D) led to decreased SUZ12 peak numbers (Figure 3A). Importantly, around 50–70% of SUZ12 peaks overlapped with H3K27me3 peaks under each condition (Figure 3B), demonstrating a strong genome-wide association between PRC2 (enzyme) and H3K27me3 (product). To determine how CTR9 refines global H3K27me3 distribution on chromatin, we allocated the SUZ12 ChIP-Rx signal on H3K27me3 peaks as well as the putative H3K27me3 regulated genes annotated in Supplementary Figure S2F. H3K27me3 peak regions (Figure 3C) and over 11000 strong H3K27me3 peak genes concurrently displayed dynamic SUZ12 changes in response to CTR9 levels by Dox treatment (Figure 3D and E), as depicted by an example captured from genome-browser (Figure 3F). We noted, however, that the magnitude of changes of SUZ12/PRC2 binding intensities were less pronounced as compared to those of H3K27me3 (compare Figure 3E with Figure 2E), especially around the newly gained H3K27me3 peak regions (Figure 3F), when CTR9 was knocked down or re-expressed. Similar to our analyses of H3K27me3 peak associated genes, we found that CTR9 loss led to increased SUZ12 peak width (Supplementary Figure S3B), which further consolidates the idea that increased PRC2 binding, at least in part, accounts for increased H3K27me3 deposition. Using the peak-annotation method (Supplementary Figure S2F) to assign SUZ12 peaks to the putative regulated genes, we identified >17 000 putative SUZ12 peak genes (Supplementary Table S5), among which 85% are protein-coding genes (Supplementary Figure S3C). Moreover, 9368 SUZ12 peak genes overlapped with H3K27me3 peak genes (Supplementary Figure S3D). Notably, a significant number of genes (2115) were identified as H3K27me3 peak genes but lacked SUZ12 binding peaks (Supplementary Figure S3D). The reason for 2155 genes harboring strong H3K27me3 signal but weak SUZ12 signals is unclear. Nevertheless, the significant overlap between SUZ12 and H3K27me3 peak associated genes underscores the concordant PRC2 complex binding and histone H3K27me3 deposition that occurs in a CTR9-dependent manner.

### CTR9 regulated genes are subjected to epigenetic regulation by PRC2, and CTR9 counteracts the establishment of H3K27me3 repressive domains

**Figure 4.**
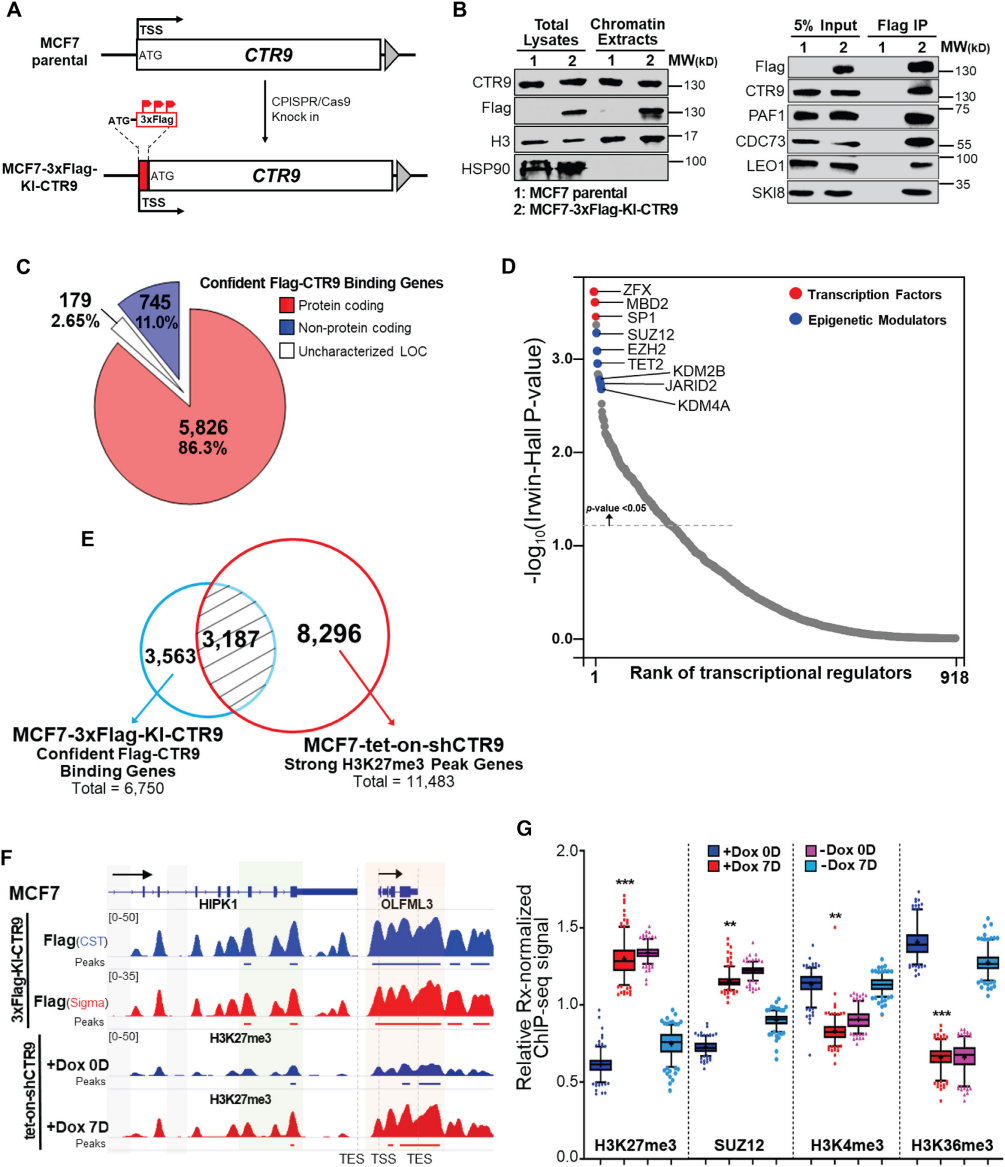
CTR9 regulated genes are subjected to epigenetic regulation by PRC2. (**A**) Scheme of 3xFlag knock-in CTR9 in MCF7 cells. (TSS: translation start site). (**B**) Western blotting demonstrates the expression of Flag-tagged CTR9 protein. HSP90 and histone H3 were used as loading controls for total lysates and chromatin extracts, respectively (left). CTR9 was pulled down using Flag antibody in MCF7-3xFlag-KI-CTR9 cells. Western blotting results showed co-immunoprecipitation of other subunits of PAFc with CTR9. MCF7 parental cells served as negative control (right). (**C**) Genomic distribution of high confident genes (*n* = 6750) regulated by CTR9 based on Flag ChIP-Rx signals in MCF7-3xFlag-KI-CTR9 cells. (**D**) Ranked dot plot of *cis*-regulatory factors prediction for Flag-CTR9 binding genes by *BART*. Top transcription factors and epigenetic modulators were highlighted in red and blue. (**E**) Venn Diagram showing the overlap between the 6750 genes harboring Flag-CTR9 binding sites and 11483 strong H3K27me3 peak associated genes identified in Supplementary Figure S2F. (**F**) Representative genome-browser snapshot of the averaged Flag (CST/Sigma) ChIP-Rx signals (*n* = 2) in MCF7-3xFlag-KI-CTR9 cells (top) and H3K27me3 ChIP-Rx signals (*n* = 2) in MCF7-tet-on-shCTR9 cells (bottom). (**G**) Box plot of relative Rx-normalized H3K27me3, SUZ12, H3K4me3 and H3K36me3 ChIP-Rx signals of 240 CTR9 regulated genes. Difference in ratios were significant (*P< 0.05; **P< 0.01; ***P< 0.001) by two-tailed *t*-test with Welch’s correction.

To investigate if genes harboring CTR9 binding sites are more likely to be subjected to H3K27me3 regulation than genes without CTR9 binding, we sought to map CTR9 genomic binding sites and derive CTR9 regulated genes from ChIP-Rx binding peaks. Because ChIP-grade CTR9 antibody is not available, we first generated a 3xFlag knock-in MCF7 clonal cell line using CRISPR/Cas9 (Figure 4A). PCR and sequencing results confirmed the correct insertion of 3xFlag epitope tag before the translation start codon of CTR9 loci (MCF7-3xFlag-KI-CTR9; Clone#5) (Supplementary Figure S4A). To verify that the Flag-tagged CTR9 protein was successfully incorporated into PAFc, we performed pulldown using an anti-Flag antibody. The results showed that Flag-CTR9 was enriched in chromatin extracts and co-immunoprecipitated with other PAFc subunits (Figure 4B). The Flag-tag knock-in does not appear to affect cell growth, since the growth rate of the MCF7-3xFlag-KI-CTR9 clone was similar to that of the parental MCF7 cells (Supplementary Figure S4B). To identify highly confident genes regulated by CTR9, we performed ChIP-Rx using two different, commercial Flag antibodies (Cell Signaling Technology and Sigma Aldrich) and annotated peaks using identical parameter settings. A total of 6750 genes were identified by overlaying Flag-CTR9 binding peaks associated genes from ChIP-Rx datasets using two Flag antibodies (Supplementary Figure S4C and Supplementary Table S6), over 85% of which are protein-coding genes (Figure 4C). Interestingly, PRC2 proteins (i.e., SUZ12 and EZH2) were predicted as *cis*-regulatory factors enriched on the putative CTR9 regulated genes (Figure 4D and Supplementary Table S7), resembling the regulatory pattern of strong H3K27me3 peaks associated genes (Figure 2F). Around 50% of genes with Flag-CTR9 binding sites overlap with those harboring strong H3K27me3 peaks (Figure 4E). Moreover, the level of H3K27me3 was responsive to CTR9 level changes on the genes containing Flag-CTR9 binding peaks, as shown in an example (Figure 4F). In addition, pathway enrichment analysis revealed that genes harboring high confidence Flag-CTR9 binding sites are often linked to pathways of transcriptional regulation (Supplementary Figure S4D), in keeping with the known functions of CTR9. TF motif analyses further identified COUP-TFII and FOXO3, both of which are known TFs that regulate gene expression of multiple pathways in breast cancer (Supplementary Figure S4E) (38,39). Together, our results reveal a potential link between CTR9 binding and PRC2 regulation. To directly examine if CTR9 regulated genes are subjected to PRC2 and H3K27me3 regulation, we analyzed the ChIP-Rx signals of H3K27me3 and SUZ12, as well as transcriptional active histone marks (H3K4me3 and H3K36me3) on previously identified 240 CTR9 regulated genes (Supplementary Table S8) (10). These genes were derived from our integrated analysis of microarray gene expression (over 1.5-fold decrease of expression upon Dox treatment) and RNA Polymerase II (RNAPII) ChIP-seq signal changes (significant interrupted RNAPII binding upon Dox treatment) in MCF7-tet-on-shCTR9 cell line, and thus are classified as high-confident CTR9 target genes. The results showed that CTR9 target genes are highly responsive to H3K27me3 and PRC2 regulation (Figure 4G). In accordance with reduced RNAPII binding and decreased gene expression, these 240 putative CTR9 target genes exhibited increased H3K27me3/SUZ12 ChIP-Rx signals which were accompanied by decreased H3K4me3/H3K36me3 signals upon CTR9 depletion (Figure 4G and Supplementary Figure S4F), and conversely, H3K27me3/SUZ12 binding on these genes were diminished by re-expression of CTR9, along with the concomitant increase of H3K4me3 and H3K36me3 signals. The H3K27me3, H3K4me3 and H3K36me3 ChIP-Rx signal intensities as well as RNAPII ChIP-seq profiles on an example gene in response to CTR9 loss was illustrated in Supplementary Figure S4G. Together, our results strongly support the notion that the CTR9 and PRC2 intricately counteract each other to establish transcriptionally active or repressive domains. This epigenetic regulation engages multiple histone modifications (e.g., H3K27me3, H3K4me3 and H3K36me3), and is reversible and highly dependent on CTR9 levels.

### CTR9 level is a determinant of PRC2 complex subtype

**Figure 5.**
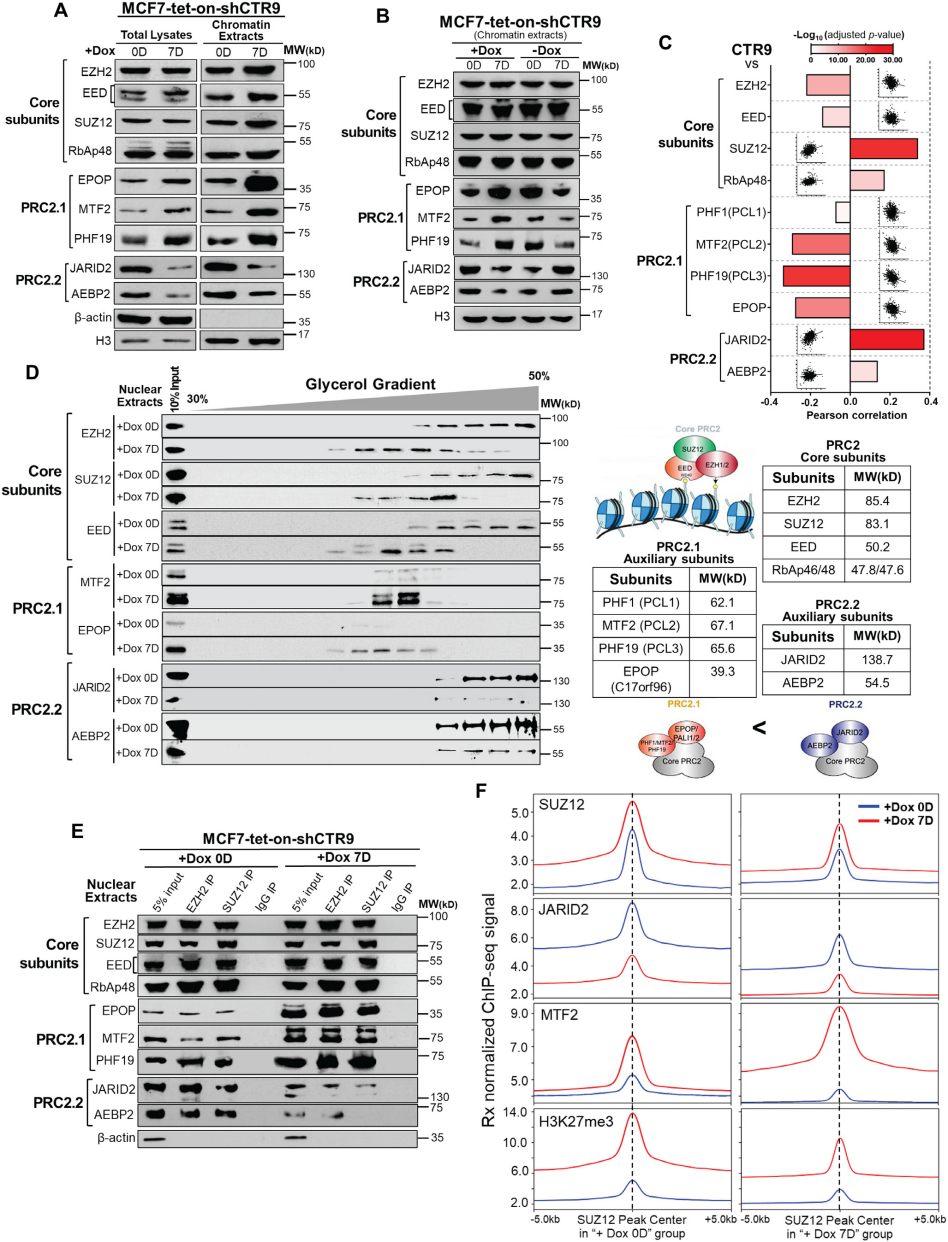
Loss of CTR9 results in a switch from PRC2.2 to PRC2.1. (**A**) Western blot analyses of PRC2 core subunits (i.e., EZH2, SUZ12, EED and RbAp48) and facultative subunits EPOP/MTF2/PHF19 for PRC2.1 and JARID2/AEBP2 for PRC2.2 in total lysates and chromatin fractions in MCF7-tet-on-shCTR9 cells treated with Dox for 0 day or 7 days. β-Actin and histone H3 were used as loading controls for total lysates and chromatin extracts, respectively. (**B**) Western blot analyses of PRC2 core subunits and facultative components in chromatin extracts from MCF7-tet-on-shCTR9 cells under indicated treatment conditions. Histone H3 served as a loading control. (**C**) Bar chart showing the Pearson correlation of CTR9 and PRC2 core and facultative subunits expression in 817 primary ER-positive breast tumor samples from TCGA. Adjusted *P*-values were transformed in -log10 manner and shown as red scale bars. (**D**) Glycerol gradient sedimentation to separate PRC2.1 and PRC2.2 subtypes in nuclear extracts of MCF7-tet-on-shCTR9 cells after 0 day or 7 days of Dox treatment (left). Molecular weight of individual PRC2 subunits were shown (right). (**E**) Co-immunoprecipitation of PRC2 auxiliary components with the core subunits (EZH2 or SUZ12) using nuclear extracts from MCF7-tet-on-shCTR9 treated with Dox for 0 day or 7 days. β-Actin was used as a loading control for input. (**F**) Composite profile of SUZ12, JARID2, MTF2 and H3K27me3 ChIP-Rx signals in ±5.0 kb range of SUZ12 peak centers.

The increased total H3K27me3 levels and broadened H3K27me3 peak-width (Figure 2C and Supplementary Figure S2E) in CTR9 KD cells are in conformity with increased PRC2 recruitment and enhanced PRC2 activity. Although SUZ12 changes coincide with H3K27me3 in response to CTR9 levels, the extent of increase of SUZ12/PRC2 peak intensity was modest as compared to the increased H3K27me3 intensities (Figure 2D versus 3D). Hence, we postulated that CTR9 knockdown might also lead to enhanced PRC2 activity. There are two mutually exclusive subtypes of PRC2: PRC2.1 and PRC2.2, which have been shown to antagonize each other and elicit different capacity for H3K27me3 deposition and propagation. For instance, loss of PRC2.2 specific subunit AEBP2 led to an increase in the amount of PALI1-containing PRC2.1 (23). Moreover, H3K27me3 levels are greatly decreased in either MTF2 single KO or PCL proteins triple KO ESCs (40). Because the levels of JARID2, a PRC2.2-specific subunit, are positively correlated with CTR9 expression in our transcriptome studies (10), we surmise that CTR9 KD may cause a PRC2 subtype switch through affecting the expression of PRC2 facultative components. Indeed, depleting CTR9 in MCF7 cells resulted in a dramatic increase in PRC2.1 facultative subunits MTF2, PHF19 and EPOP, and a decrease in JARID2 and AEBP2, the PRC2.2 facultative subunits (Figure 5A) in total cell lysates and chromatin fractions, whereas the levels of four core subunits remained unchanged. Similar observations were made in two stable MCF7 CTR9 KD cell lines (Supplementary Figure S5A). To investigate if the regulation of PRC2 facultative subunits by CTR9 is reversible, we measured the expression of five facultative subunits of respective PRC2 subtypes in MCF7-tet-on-shCTR9 cells. The expression of PRC2 facultative subunits, and CTR9 protein levels was strongly correlated (Figure 5B). The decrease of PRC2.2-specific subunits JARID2 and AEBP2 could be partially restored by CTR9 re-expression and was accompanied by the opposite changes in PRC2.1-specific MTF2, PHF19 and EPOP proteins (Figure 5B). The negative correlation between CTR9 and PRC2.1 facultative subunits (PHF1/MTF2/PHF19 and EPOP) at the mRNA level was also observed in 817 ER-positive primary breast tumor samples in TCGA (Figure 5C). To test if loss of CTR9 is sufficient to alter PRC2 subtype equilibrium, we performed glycerol gradient sedimentation to separate two PRC2 subcomplexes from nuclear fractions, based on their differences in molecular weight (i.e., calculated PRC2.2 molecular weight is nearly 100 kDa larger than PRC2.1). When CTR9 was knocked down (+Dox 7D), the PRC2 complex changed from PRC2.2 to PRC2.1, as detected by subtype-specific subunit levels by western blotting (Figure 5D). Co-immunoprecipitation using antibodies against EZH2 and SUZ12, the core subunits of the PRC2, was able to pulldown other core and auxiliary proteins from nuclear extract, and the results agree with PRC2.2 to PRC2.1 subtype switching in response to depletion of CTR9. Specifically, PRC2 core subunits tended to co-precipitate PRC2.2-specific JARID2 and AEBP2 proteins when CTR9 was expressed. Knocking down CTR9 resulted in enrichment of PRC2.1-specific subunits MTF2/PHF19 and EPOP (Figure 5E). Biotinylated H3K27me3 peptide pulldown (Supplementary Figure S5B) further confirmed the PRC2.2 to PRC2.1 switch. Thus, we conclude that this PRC2 subtype switch is likely due to the relative abundance of facultative subunits, which are highly responsive to CTR9 levels. Because PRC2.1 was proposed to elicit higher histone methylation activity than PRC.2.2, we prepared nuclear extract from CTR9 inducible KD MCF7 cells with and without Dox treatment and used them as enzyme sources for the *in vitro* methylation assay. Biotinylated recombinant human histone H3.1 or recombinant human poly-nucleosome were used as substrates (Supplementary Figure S5C). The results showed that nuclear extract from ‘+Dox 7D’ cells elicited higher histone methyltransferase (HMT) activity than that from ‘+Dox 0D’ cells on H3K27, regardless of the substrates used (Supplementary Figure S5C). In addition to HMT activity changes *in vitro*, we performed ChIP-Rx for JARID2, a PRC2.2 specific subunit, and MTF2, a PRC2.1 specific subunit. Upon CTR9 depletion (+Dox 7D), decreased JARID2 and increased MTF2 genomic association were observed across the SUZ12 peaks (Figure 5F). Despite the inverse intensities of JARID2 and MTF2 ChIP-Rx, their binding peaks overlap with those of SUZ12 and H3K27me3, as shown by an example gene (Supplementary Figure S5D). Further analyses of the global SUZ12, JARID2, MTF2 and H3K27me3 normalized read counts across all SUZ12 peaks confirmed a substantial shift from JARID2 containing PRC2.2 to MTF2 containing PRC2.1 in response to CTR9 depletion, as illustrated in the scatter plot (Supplementary Figure S5E).

We further investigated whether CTR9 regulates the expression of PRC2 auxiliary proteins in a direct or indirect manner by employing two different approaches. First, we performed RT-qPCR to examine the mRNA levels of PRC2 core and subtype accessory genes in CTR9 inducible knockdown MCF7 cells with or without Dox treatment. The mRNA levels of PRC2.2 subunits (JARID2/AEBP2) were significantly decreased, whereas the mRNA levels of two PRC2.1 genes (MTF2/EPOP) increased when CTR9 was knocked down (Supplementary Figure S6A). Second, we examined whether these PRC2 accessory subunits contain Flag-CTR9 binding peaks in our ChIP-Rx analysis. We used two different commercial Flag antibodies for ChIP-Rx. The results showed that Flag-CTR9 binding signals were undetectable in PRC2.1 genes such as PHF1 and EPOP, but high in PRC2.2 accessory genes (JARID2 + AEBP2) (Supplementary Figure S6B and S6C). JARID2 is among the 240 putative CTR9 regulated genes we had previously identified since JARID2 gene expression and RNAPII binding on *JARID2* are positively correlated with CTR9 levels (9,10). One exception of PRC2.1 accessory genes is PHF19 (PCL3), as we observed Flag-CTR9 binding near PHF19 (Supplementary Figure S6B and S6C), yet the PHF19 mRNA is not regulated by CTR9 levels (Supplementary Figure S6A). Previous studies showed that PHF19-associated PRC2.1 is at least 10-fold less abundant as compared with MTF2-containing PRC2.1 in most cell types (41,42). The reason for having Flag-CTR9 ChIP-Rx signal near PHF19, whereas mRNA levels are not regulated by CTR9, is unclear. Nonetheless, based on the combined data from Flag-CTR9 ChIP-Rx and RT-qPCR, we concluded that the PRC2.2 subunits are direct targets of CTR9, while genes encoding PRC2.1 accessory subunits are unlikely to be direct targets of CTR9. Interestingly, several studies have also reported the upregulation of PRC2.1 subunits in response to the loss of PRC2.2 subunits (23,40,43–45). However, the molecular mechanism underlying compensation of PRC2.1 in response to loss of PRC2.2 remains unknown.

These data collectively support the model that CTR9 loss triggers the abundance changes of PRC2 facultative subunits by directly decreasing the mRNA levels of PRC2.2 subunits (JARID2/AEBP2), leading to a switch from the less active PRC2.2 to more active PRC2.1, which facilitates the propagation of H3K27me3 repressive domains.

### PRC2 subtype switching partially phenocopies the genome-wide increase of H3K27me3 caused by CTR9 knockdown but does not affect cell growth or transcription

**Figure 6.**
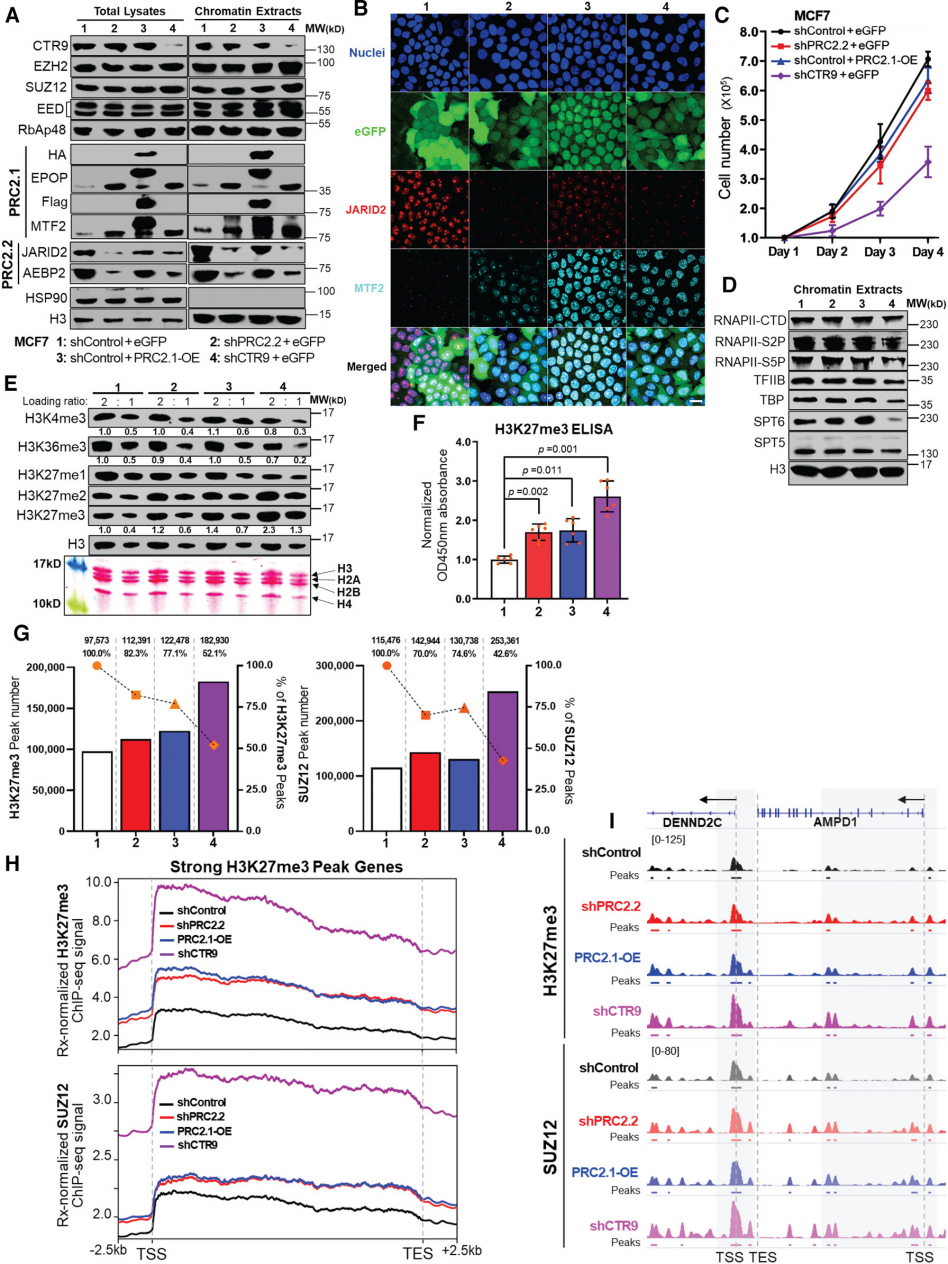
PRC2 subtype switching results in a less robust increase in H3K27me3 compared to CTR9 depletion. (**A**) Western blot analyses of PRC2 core subunits (i.e., EZH2, SUZ12, EED and RbAp48) and facultative subunits (HA)-EPOP/ (3xFlag)-MTF2 for PRC2.1 and JARID2/AEBP2 for PRC2.2 in total lysates and chromatin fractions of MCF7 cells. The four engineered cell lines are Control KD (1: shControl + eGFP), PRC2.2 KD (2: shPRC2.2 + eGFP), PRC2.1 overexpression (3: shControl + PRC2.1-OE) and CTR9 KD (4: shCTR9 + eGFP). HSP90 and histone H3 were used as loading controls for total lysates and chromatin extracts, respectively. (**B**) Representative images of immuno-fluorescence staining of PRC2.2-JARID2 (37), PRC2.1-MTF2 (cyan) and nuclei (blue) in cell lines in Figure 6A. eGFP (green) was served as a reporter control. 100× scale bar shown at the bottom right applies to all images. (**C**) Proliferation measured by four-day cell counting of cell lines in Supplementary Figure S7A. Data are represented as mean ± SD (*n* = 6). (**D**) Western blot analyses of indicated proteins in chromatin fractions of cell lines in Supplementary Figure S7A. H3 were used as loading controls. (**E**) Western blot analyses of H3K4me3, H3K36me3 and H3K27me1, 2, 3 levels in cell lines in Supplementary Figure S7A (top). Ponceau S staining of histones are shown in two-fold loading ratio (bottom). The band intensities of H3K4me3/H3K36me3/H3K27me3 were quantified using ImagePro after normalizing with H3 loading controls. (**F**) H3K27me3 levels measured by ELISA assays. Data were normalized to the respective total histone H3 levels and are represented as mean ± SD (*n* = 6). *P*-values were calculated using a two tailed *t*-test with Welch’s correction. (**G**) H3K27me3 (left) and SUZ12 (right) peak numbers (bar graph, left *Y*-axis) and percentage% (dot-plot, right *Y*-axis) of total H3K27me3 (left) or SUZ12 (right) peaks from each group that overlapped with H3K27me3 or SUZ12 peaks in shControl group. Peak numbers and percentage% of overlapping peaks are shown on the top of each bar. (**H**) Average profiles of Rx-normalized H3K27me3 (top) and SUZ12 (bottom) ChIP-seq signals at ±2.5 kb of TSS-TES regions of 11483 strong H3K27me3 peak genes identified in Supplementary Figure S2F in cell lines in Supplementary Figure S7A. (**I**) Representative genome-browser snapshot of the averaged H3K27me3 (top) and SUZ12 (bottom) ChIP-Rx signal (*n* = 2) in Control KD, PRC2.2 KD, PRC2.1 OE and CTR9 KD MCF7 cells. Each signal-track represents the mean of two biological replicates.

To examine if switching of PRC2 subtypes phenocopies the genome-wide increase of H3K27me3 caused by knocking down CTR9, we knocked down PRC2.2 or overexpressed PRC2.1 using a lentiviral based duo-target shRNAs delivery system (46,47) and a polycistronic overexpression (OE) system (48) in MCF7 cells (Supplementary Figure S7A). After two rounds of lentiviral infection, two PRC2.2 subunits— JARID2 and AEBP2 could be simultaneously knocked down in shPRC2.2 cells (group 2). Group 3 represents PRC2.1-OE cells simultaneously overexpressing two PRC2.1 subunits—Flag-tagged MTF2 and HA-tagged EPOP. As a control, we used the same strategy to knockdown CTR9 by expressing two validated shRNAs cistronically (group 4). All groups express eGFP to normalize for infection efficacy. Western blot results confirmed the successful knockdown of JARID2 and AEBP2 in shPRC2.2 (group 2 in Figure 6A) as well as overexpression of Flag-MTF2 and HA-EPOP in PRC2.1-OE (group 3 in Figure 6A) in total lysates and chromatin extracts. Notably, like shCTR9 (group 4 in Figure 6A), knocking down PRC2.2 subunits led to increased expression of MTF2 and EPOP, two PRC2.1 specific subunits. On the contrary, PRC2.1 subunit overexpression resulted in a modest decrease of JARID2 and AEBP2, two PRC2.2-specific subunits. The expression of PRC2 subtype-specific subunits was visualized by immunofluorescence staining of JARID2 and MTF2 (Figure 6B). Neither PRC2.1 KD nor PRC2.1-OE appeared to affect MCF7 growth rate in culture (Figure 6C) or colony formation (Supplementary Figure S7B) as compared to the shControl group. This contrasts with the growth inhibitory effects induced by knocking down CTR9 (Figure 6C and Supplementary Figure S7B). We posit that the impaired cell growth caused by knocking down CTR9 is likely attributed to ablation of transcription, causing growth defects (9,10). Transcription factor II B (TFIIB), TATA-binding protein (TBP) and serine 5 phosphorylated C-terminal domain (CTD) of RNAPII (S5P) are all involved in the formation of the RNA polymerase II preinitiation complex (PIC), which is a pre-requisite for transcription initiation (49–51). The serine 2 phosphorylated CTD of RNAPII (S2P), in combination with SPT5/6 elongation factors, are indispensable for the release of paused RNAPII elongation complex during transcription elongation (4,52,53). Indeed, western blot results show that proteins required for productive transcription initiation and elongation were decreased by loss of CTR9 but not affected by PRC2 subtype switching (Figure 6D). This result suggests that manipulating the PRC2 subtype-balance partially phenocopies CTR9 loss because it does not interfere with general transcriptional machinery. We went on to examine if manipulating PRC2 subtypes caused changes in total H3K27me3 levels. As expected, a modest increase on H3K27me3 levels was observed in PRC2.2 KD or PRC2.1-OE cells, whereas levels of H3K4me3 and H3K36me3, two transcriptionally active histone marks, remain stable (Figure 6E). Stable CTR9 KD led to a dramatic increase of H3K27me3, as well as a decrease of H3K4me3 and H3K36me3 (Figure 6E), as observed in the inducible CTR9 KD system (Figure 2). The WB results were confirmed by an H3K27me3 ELISA assay (Figure 6F) and immunofluorescence staining (Supplementary Figure S7C). To investigate if PRC2 subtype switching alone can affect PRC2/H3K27me3 chromatin binding, as observed in CTR9 inducible KD cells, we performed ChIP-Rx using antibodies against H3K27me3 and SUZ12 (PRC2 core subunit) using the aforementioned cell lines. Quantification of peak numbers showed that H3K27me3 and SUZ12 peak numbers were higher in PRC2.2 KD and PRC2.1-OE cells as compared to the shControl group (1.2- to 1.3-fold), and the majority of peaks overlapped among these groups (>70%) (Figure 6F). A greater increase in H327me3 (∼2-fold) and SUZ12 (>2-fold) peaks was detected in CTR9 stable KD cells, and around 50% of those H3K27me3 or SUZ12 peaks identified in CTR9 KD cells were not found in shControl cells (Figure 6G). To interrogate how PRC2 subtype switching affects the distribution of H3K27me3 and PRC2 on chromatin, we allocated the H3K27me3 and SUZ12 ChIP-Rx signals to 11483 putative H3K27me3 regulated genes identified in Supplementary Figure S2F. The results show that either PRC2.2 KD or PRC2.1-OE partially rescued H3K27me3 or SUZ12 ChIP-Rx signals on H3K27me3 peak genes as compared to CTR9 KD (Figure 6H and Supplementary Figure S7D). H3K27me3 and SUZ12 profiles of an example gene in shControl, shPRC2.2, PRC2.1-OE and shCTR9 cells are shown in Figure 6I. In summary, depleting CTR9 stalled transcription, affected cell growth and caused PRC2 subtype switching from PRC2.2 to PRC2.1. Manipulating the subtype of PRC2 (i.e, PRC2.2 KD nor PRC2.1-OE) alone can partially phenocopy CTR9 KD, as seen through PRC2 and H3K27me3 chromatin profiling, but does not affect general transcription and cell growth.

### CTR9 depletion sensitizes breast cancer cells to PRC2 complex inhibitors

**Figure 7.**
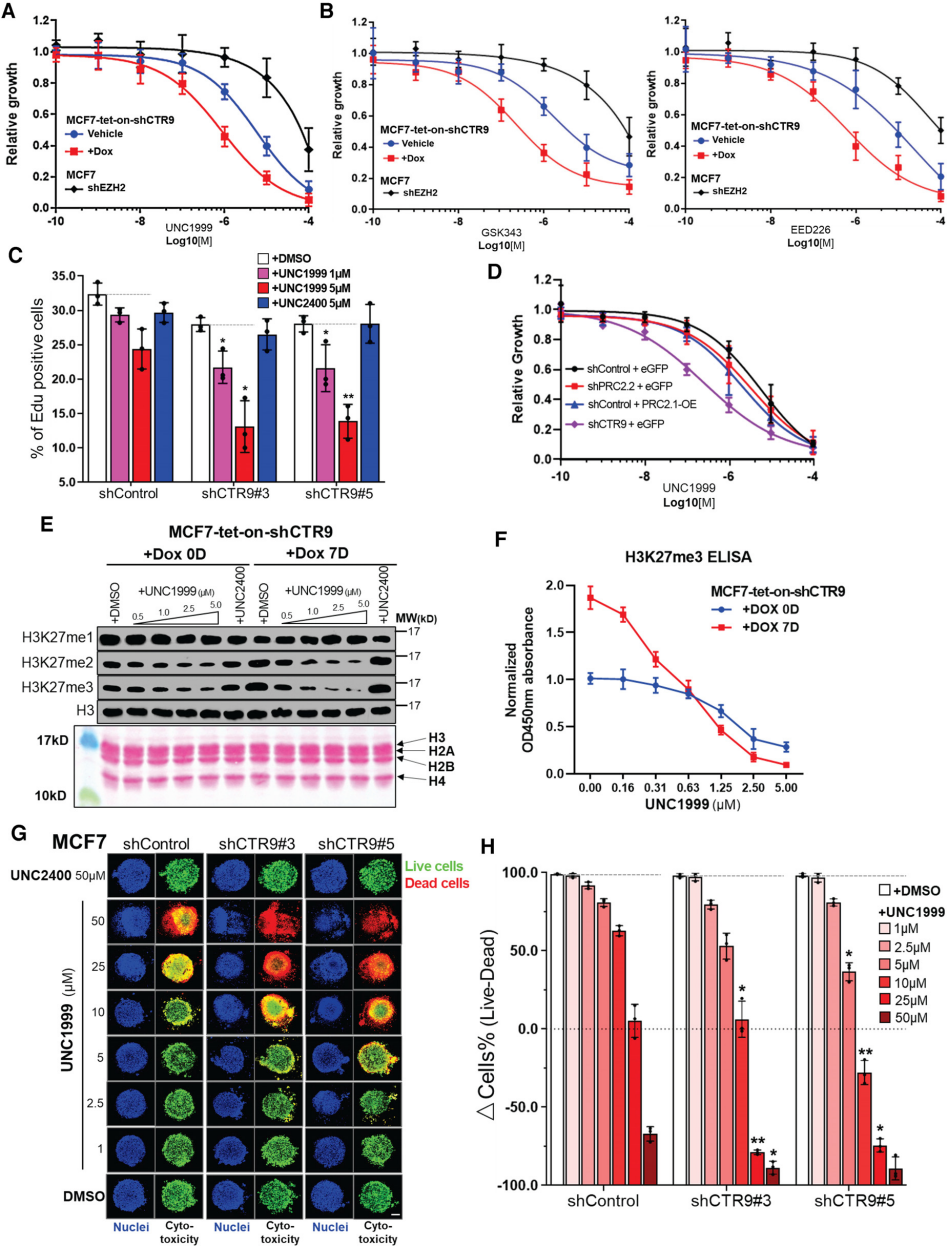
Depletion of CTR9 sensitizes MCF7 cells to PRC2 inhibitors. (**A**) Cell viability measured by MTT assays after treating MCF7-shEZH2 or MCF7-tet-on-shCTR9 cells (Vehicle or Dox) with increasing doses of UNC1999. Data are represented as mean ± SD (*n*= 6). IC_50_ were calculated through non-linear regression against log(inhibitor) response model. (IC50: Vehicle = 4.6 ± 0.3 μM; +Dox = 1.0 ± 0.1 μM). (**B**) Cell viability measured by MTT assays after treating MCF7-shEZH2 or MCF7-tet-on-shCTR9 cells (Vehicle or Dox) with increasing doses of GSK343 (left) or EED226 (right). Data are represented as mean ± SD (*n*= 6). (**C**) Quantification of % EdU positive cells from control shRNA or shCTR9 (#3 and #5) MCF7 cells treated with DMSO, UNC1999 or UNC2400. Data are represented as mean ± SD (*n*= 3). The difference of % EdU positive cells between DMSO and each group of UNC1999 treatment were calculated and corresponding *P*-value of Welch’s *t*-test were shown (*: *P*< 0.05; **: *P*< 0.01). (**D**) Cell viability measured by MTT assays after treating Control KD, PRC2.2 KD, PRC2.1 OE and CTR9 KD MCF7 cells with increasing doses of UNC1999. Data are represented as mean ± SD (*n*= 6). (**E**) Western blot analyses of H3K27me1,2,3 levels in MCF7-tet-on-shCTR9 cells (+Dox 0D or + Dox 7D) treated with increasing doses of UNC1999 or 5.0 μM UNC2400, a negative paralog. Histone H3 was used as a loading control (top). Ponceau S staining of histones were shown in two-fold loading ratio (bottom). (**F**) H3K27me3 levels measured by ELISA in MCF7-tet-on-shCTR9 cells (+Dox 0D or + Dox 7D) treated with increasing doses of UNC1999. Data were normalized to the respective total histone H3 levels and are represented as mean ± SD (*n*= 6). (**G**) Representative confocal images of 3D spheroids of shControl or shCTR9 expressing MCF7 cells after treatment with DMSO or increasing doses of UNC1999 for 24 h. UNC2400 (negative paralog) serves as a negative control. Nuclei (blue), live cells with ubiquitous esterase activity (green) and dead cells with impaired cell membrane were shown by immunofluorescence staining. 10× scale bar shown at the bottom right applies to all images. (**H**) Quantification of the cytotoxic response of shRNA or shCTR9 expressing MCF7 cells grew in 3D spheroids against UNC1999. The difference of live cells percentage and dead cells percentage were measured by fluorescence absorbance of calcein AM and EthD-1, respectively. Data are shown in mean ± SD; (*n*= 3) *P< 0.05; ** *P*< 0.01.

High levels of EZH2 and H3K27me3 in ER-negative breast cancer patients predict poor overall survival, and EZH2 inhibitor GSK343 has been shown to elicit robust inhibition of ER-negative breast tumor growth in a preclinical model (54). The remarkable increase of total H3K27me3 levels upon depletion of CTR9 implies an addiction of cells to H3K27me3 for survival. If this were true, we expect that CTR9 knockdown cells would elicit higher sensitivity to EZH2 inhibitors than the parental cells. Cell viability was measured after treatment with UNC1999, a chemical inhibitor targeting both EZH2 and EZH1, and UNC2400, a structurally similar but inactive analog compound, to exclude the possibility of off-target effects (55). As expected, CTR9 KD MCF7 cells were more sensitive to UNC1999 than parental MCF7 cells, as shown by MTT assays. EZH2 KD MCF7 cells (shEZH2) were included as a negative control (Figure 7A), and, indeed, were insensitive to UNC1999. To exclude a drug-specific effect, we examined two additional mechanistically distinct PRC2 inhibitors, GSK343 and EED 226 (56,57). The results were similar to those of UNC1999 (Figure 7B). In addition to MTT assays, we counted EdU positive proliferating cells in the presence of DMSO, UNC1999 and UNC2400. The results confirmed that CTR9 KD MCF7 cells were more sensitive to EZH2 inhibitors than parental cells (Figure 7C). Collectively, our results demonstrate that depletion of CTR9 leads to increased sensitivity toward PRC2 inhibitors. Consistent with the partial rescue of CTR9 KD phenotypes (Figure 6) by PRC2 subtype switching, MCF7 cells with PRC2.2 KD or PRC2.1 overexpression showed a modest increase in sensitivity to UNC1999 as compared to CTR9 KD (Figure 7D). To investigate whether loss of CTR9 renders cells more sensitive to EZH2 inhibitors due to H3K27me3 addiction, we examined changes in H3K27me3 levels in response to increasing concentrations of UNC1999 in the presence or absence of CTR9 in Dox-inducible CTR9 KD cells (Figure 7E). Indeed, a more rapid loss of H3K27me3 was observed in CTR9 KD cells (+Dox) as compared to CTR9 expressing cells (-Dox) when treated with UNC1999, indicating that CTR9 KD cells become addicted to H3K27me3. The dose-dependent decrease of H3K27me3 levels in response to increasing concentrations of UNC1999 was validated by H3K27me3 ELISA (Figure 7F). Treatment with DMSO and UNC1999 negative paralog UNC2400 served as background and negative controls. The increased H3K27me3 levels and sensitivity to EZH2 inhibitors prompted us to investigate whether UNC1999 causes elevated apoptosis or necrosis upon CTR9 depletion. We performed flow cytometry analyses after labeling cells with propidium iodide (PI) and annexin V-FITC and the results showed that both apoptotic and necrotic cells were detected in CTR9 KD cells, but not in the shControl cells after 1–5 μM UNC1999 treatment for 3 days (Supplementary Figure S8A). When the concentration of UNC1999 increased to 12.5 μM, nearly all the CTR9 KD MCF7 cells became apoptotic or necrotic; however, ∼30% of control KD cells remained viable (Supplementary Figure S8A). Next, we employed a duo-fluorescence based cell cytotoxicity kit to quantify the differential cytotoxic response between Control KD and CTR9 KD cells towards UNC1999, both in 2D monolayer (Supplementary Figure S8B) and in 3D tumor spheroids (Figure 7G). When treated with ascending concentrations of UNC1999 from 1 to 50 μM for 24 h, CTR9 KD MCF7 3D spheroids (shCTR9#3/shCTR9#5) elicited stronger cytotoxic response (more red cells and less green cells) than the control group (shControl). UNC2400 (50 μM) serves as a negative control, and no cytotoxic effect was detected (Figure 7G). Similar results were observed in 2D cultured cells (Supplementary Figure S8B). Differences in the percentage of live and dead cells in both 2D monolayer and 3D spheroids were quantified, respectively (Supplementary Figure S8C and Figure 7H). These results reinforced that CTR9 KD cells are more sensitive to EZH2 inhibitors. Collectively, our results suggest that CTR9 depletion causes cells to become addicted to H3K27me3, rendering them more sensitive to EZH2 inhibitors, as measured by inhibition of growth/proliferation, as well as induction of apoptosis and necrosis in MCF7 cells.

## DISCUSSION

In this study, we reported that CTR9 governs the establishment of H3K27me3 repressive domains, beyond its well-characterized functions in transcriptional regulation and modulation of transcription-coupled histone modifications (i.e., H2Bub, H3K4me3, H3K36me3). This discovery provides an explanation for the discrepancy between the phenotypes observed in lower eukaryotes (i.e., yeast) and multicellular organisms when CTR9 is depleted. While CTR9 is not an essential gene in *S. cerevisiae* (58,59), CTR9 knockout causes early embryonic lethality in higher eukaryotic organisms such as *Drosophila* (60), zebrafish (61) and mouse (62). The requirement of CTR9 in preimplantation development of mice resembles the phenotypes that result from lacking core PRC2 subunits SUZ12, EZH2 and EED (63). While PRC2 and H3K27me3 play crucial roles in establishing repressive chromatin regions, and safeguarding cell identity in multicellular organisms (12), neither PRC2 nor histone H3K27me3 exists in yeast. Our results indicate that while CTR9 maintains transcriptional control across species, controlling PRC2-repressive domains is a new function acquired over the course of evolution. We surmise that the embryonic lethality of CTR9-null metazoans is more likely attributed to deregulated H3K27me3 than the transcription inhibition *per se*.

CTR9 is not among the 148 PRC2 interaction partners that are enriched in EZH2 and SUZ12 immunoprecipitants in ESCs (64). Therefore, the significantly elevated levels and genome-wide distribution of H3K27me3 upon loss of CTR9 is unexpected, which implies that, despite no physical interaction, CTR9 is functionally related to PRC2. Given that CTR9 is the scaffold protein of PAFc which regulates multiple steps of transcription, and that the switch of PRC2 subtypes is accompanied by loss of CTR9, we envision that at least three mechanisms may collectively contribute to the establishment of H3K27me3 domains: inhibition of transcription, enhanced PRC2 recruitment and alteration of PRC2 activity.

First, inhibition of transcription alone has been shown to be sufficient for gain of H3K27me3 in the gene bodies (65,66). In mouse ESCs, transcriptional inhibition by RNAPII inhibitors 5,6-dichloro-1-beta-D-ribofurano-sylbenzimidazole riboside (DRB), and triptolide, induces genome-wide ectopic PRC2 recruitment (65). Hosogane *et al.* also reported that abrogation of transcription start sites by CRISPR/Cas9 induces accumulation of H3K27me3 in the gene bodies (66). Emerging evidence has shown that the active histone marks inhibit PRC2 activity in transcribed regions (67) and that both H3K4me3 and H3K36me2/3 are inhibitory to PRC2 activity (68,69). Because H3K4me3 and H3K36me3 are associated with actively transcribed genes, transcription inhibition by loss of CTR9 could result in H3K27me3 accumulation in gene bodies. Indeed, we have previously shown that RNAPII binding, H3K4me3 and H3K36me3 are significantly decreased by knocking down CTR9 in MCF7 cells (9,10). In this study, we further confirmed a dramatic decrease in proteins involved in the preinitiation complex (RNAPII-S5P, TFIIB and TBP), as well as in the active elongation complex (RNAPII-S2P and SPT5/6) upon CTR9 KD (Supplementary Figure S9A and S9C). Concordantly, H3K4me3 and H3K36me3, two transcriptional active histone marks, were also decreased when CTR9 was depleted (Supplementary Figure S9B and S9C). On H3K27me3 responsive genes, reduction of H3K4me3 and H3K36me3 ChIP-Rx signals by Dox treatment in inducible CTR9 KD cells inversely correlated with gains of H3K27me3 (Supplementary Figure S10), supporting that ablation of transcription and eviction of active histone marks are prerequisites for H3K27me3 deposition in the gene proximal regions. Stable levels of H3K4me3 and H3K36me3, but increased levels of H3K27me3 in PRC2.2 KD and PRC2.1-OE cells indicate that inhibition of transcription by CTR9 depletion precedes PRC2 subtype switching. Our data support that CTR9 depletion blocked transcription, which is proceeded by PRC2 subtype switching from PRC2.2 to PRC2.1 to increase genome wide H3K27me3 levels and distribution.

Second, H3K27me3 levels are correlated with PRC2 duration on chromatin. PRC2 alone exhibits the lowest residency time on chromatin through a hit and run mechanism, but PRC2-chromatin association could be stabilized when complexed with JARID2 when forming PRC2.2 or stabilized by MTF2 in the PRC2.1 at an even greater level (22). As expected, our ChIP-Rx results showed that although >50% of genes harboring SUZ12/PRC2 binding sites overlap with those containing H3K27me3, some genes contain only H3K27me3 or SUZ12 (Supplementary Figure S3D). Those potent H3K27me3 responsive regions without an obvious increase of SUZ12/PRC2 binding could be due to the dissociation of SUZ12 after marking H3K27me3, the efficacy of ChIP-Rx using SUZ12, or corresponding to the distal spreading sites with attenuated PRC2 binding. Additional H3K27me3 and SUZ12 ChIP-Rx experiments with the gradual decline of CTR9 expression will help distinguish the initiation or propagation of PRC2 association events.

Third, we envision that the PRC2 subtype switching is a major mechanism accounting for the global H3K27me3 increase. JARID2, a PRC2.2-specific subunit, contains a Jumonji-C domain, but it has no enzymatic activity of its own (70). A previous study showed that depletion of AEBP2 results in up-regulation of PALI1/2 (23), and conversion from JARID2-containing PRC2.2, to the MTF2-containing PRC2.1 complex (44). Interestingly, a recent study reported that chromatin occupancy of PRC2 and H3K27me3 increased dramatically when PRC2.1 was forced to form in human induced pluripotent stem cells (71). Moreover, JARID2 knockout in differentiated ESCs causes aberrant deposition of H3K27me3 in intergenic regions of the genome (72). This is analogous to the effects of loss of CTR9, where a decrease in JARID2 and AEBP2 levels is accompanied by an increase of H3K27me3 domains in the intergenic regions (Supplementary Figure S11), and concomitantly, PRC2 subtype switching from PRC2.2 to PRC2.1 (Figure 5). Knocking down PRC2.2 or overexpressing PRC2.1 can promote PRC2 binding and H3K27me3 expansion, although this effect is not as robust as knocking down CTR9 (Figure 6). Therefore, we reason that the loss of CTR9 resulted in a decrease of JARID2 and AEBP2 expression, leading to the formation of the more active PRC2.1 complex, and thus the elevated H3K27me3 levels. In support of this model, MTF2 and other PCL proteins have been shown to bind CpG-rich DNA, and knockout of MTF2 in mESCs leads to reduced SUZ12 binding at CpG islands and concomitant depletion of H3K27me3 (73). Our data infer a chromatin surveillance mechanism: In the presence of CTR9, the predominant form of PRC2 is PRC2.2 to maintain the basal levels of H3K27me3. When CTR9 is removed, PRC2.1 replaces PRC2.2 to effectively establish and expand the H3K27me3 repressive domains, including regions with stalled transcriptional machinery due to loss of CTR9. Collectively, our studies reveal the complex mechanisms by which CTR9 demarcates PRC2-mediated H3K27me3 domains, and the previously unidentified interplay between transcriptional activation machinery and transcriptional repressive complexes. In our scenario, transcription blockade was introduced by depletion of CTR9, followed by increased H3K27me3 levels and expansion of H3K27me3 domains, which are likely results of enhanced recruitment of PRC2 to chromatin, as well as PRC2.2 to PRC2.1 switching. Although these findings were made in breast cancer cell lines, the general principal for the establishment of H3K27me3 domains governed by CTR9 is likely conserved in other biological systems (e.g., ESCs).

Mutations in subunits of PRC2 have been increasingly identified in multiple cancers, resulting in changes in the global levels, as well as the genome-wide distribution of H3K27me3 (24). These changes in cancer cells often confer context-dependent blocks to cellular differentiation and promote oncogenic signaling pathways. Either overexpression of EZH2 or inactivation of negative regulators of PRC2 augments the dependency of cancer cells on H3K27me3 and PRC2, indicating that PRC2 inhibition can augment the therapeutic vulnerability of cancer cells (74). For example, acute myeloid leukemia and multiple myeloma cell lines with KDM6A mutations are more sensitive to EZH2 inhibitors than KDM6A wild-type expressing lines (75,76). We found that either inducible or permanent CTR9 KD MCF7 cells elicit increased sensitivity to PRC2 inhibition, indicating that loss of CTR9 renders cells more addicted to H3K27me3 and PRC2. GSK126 and EPZ-6438, two EZH2 specific inhibitors, are under clinical investigation for treating lymphomas (25). In 2020, a first-in-class, orally bioavailable EZH2 inhibitor, tazemetostat, received accelerated approval by FDA, for treatment of epithelioid sarcoma. Furthermore, tazemetostat appears to be promising to treat patients with B-cell NHL and other solid tumors (77). Because depletion of EZH2 suppressed ER-negative tumor growth and metastasis in preclinical models (78), EZH2 has emerged as a potential therapeutic target for triple-negative breast cancer (TNBC). Our data that TNBC cell lines display higher levels of EZH2, H3K27me3 as well as PRC2.1-specific subunits (Supplementary Figure S12) support the application of PRC2 inhibitors in treating TNBC. In addition, depletion of CTR9 in ER-positive cells increase sensitivity of cells to PRC2 inhibition by nearly 10-fold, which suggests that EZH2 inhibitors may also be applicable to CTR9 low expressing, ER-positive breast cancer. We speculate that CTR9 levels, rather than ER status, is a predictive biomarker for PRC2 dependency in breast cancer cells. Since CTR9 depletion generates therapeutic vulnerability to pharmacological inhibition of PRC2, the CTR9 expression levels may be used as a biomarker for predicting PRC2 dependency and EZH2 inhibitor sensitivity in broad cancer types.

Our findings that CTR9 demarcates PRC2-mediated H3K27me3 levels and genomic distribution provide unique insights as to how transcriptionally active states are converted to repressive chromatin regions. CTR9 silencing results in the loss of imprinted genes during preimplantation development in mice (62), which may cause genome instability. Exon 9 deletion of *CTR9* were recently discovered in Wilms tumors (8). Whether H3K27me3 is elevated in CTR9-mutated Wilms tumors, and whether CTR9-mutant expressing tumors are sensitive to EZH2 inhibitors, awaits investigation. The new function of CTR9 in regulating PRC2-repressive H3K27me3 domains opens new avenues for understanding the biological functions of CTR9 in development and broad cancer types, and for exploring the possibility of using CTR9 as a biomarker to select cancer patients who are responsive to epigenetic therapies targeting PRC2 complex.

## DATA AVAILABILITY

Published sequencing datasets (Microarray/RNAPII ChIP-seq) analyzed in this paper could be downloaded from Gene Expression Omnibus (GEO) through accession number GSE73388 and GSE80728. All high-throughput next-generation sequencing ChIP-Rx datasets generated for this paper have also been submitted to GEO database through accession number GSE173176 and GSE189796. Nucleotide sequence of 3xFlag knock in at the start codon of *CTR9* loci was uploaded to GenBank with accession number BankIt2453082 Seq1, MZ018224. Mass spectrometry data for quantification of histone modifications were submitted to ProteomeXchange under dataset identifier PXD028269. Flow cytometry and RT-qPCR experiments are uploaded to DRYAD with identifier doi: 10.5061/dryad.r7sqv9sd7.

## Supplementary Material

gkac047_Supplemental_Files

## Notes

Present address: Junfeng Huang, CAS Key Laboratory of Regenerative Biology, Guangdong Provincial Key Laboratory of Stem Cell and Regenerative Medicine, GIBH-HKU Guangdong-Hong Kong Stem Cell and Regenerative Medicine Research Centre, Guangzhou Institutes of Biomedicine and Health, Chinese Academy of Sciences, 510530, Guangzhou, China.

Present address: Hao Zeng, H3 Biomedicine, Cambridge, MA 02139, USA.

## References

[1] Van Oss S.B. , Cucinotta C.E., Arndt K.M. Emerging insights into the roles of the paf1 complex in gene regulation. Trends Biochem. Sci. 2017; 42:788–798. 28870425 10.1016/j.tibs.2017.08.003 PMC5658044

[2] Zhu B. , Mandal S.S., Pham A.D., Zheng Y., Erdjument-Bromage H., Batra S.K., Tempst P., Reinberg D The human PAF complex coordinates transcription with events downstream of RNA synthesis. Genes Dev. 2005; 19:1668–1673. 16024656 10.1101/gad.1292105 PMC1176003

[3] Chu X. , Qin X., Xu H., Li L., Wang Z., Li F., Xie X., Zhou H., Shen Y., Long J. Structural insights into paf1 complex assembly and histone binding. Nucleic Acids Res. 2013; 41:10619–10629. 24038468 10.1093/nar/gkt819 PMC3905892

[4] Vos S.M. , Farnung L., Boehning M., Wigge C., Linden A., Urlaub H., Cramer P. Structure of activated transcription complex pol II-DSIF-PAF-SPT6. Nature. 2018; 560:607–612. 30135578 10.1038/s41586-018-0440-4

[5] Yu M. , Yang W., Ni T., Tang Z., Nakadai T., Zhu J., Roeder R.G. RNA polymerase II-associated factor 1 regulates the release and phosphorylation of paused RNA polymerase iI. Science. 2015; 350:1383–1386. 26659056 10.1126/science.aad2338 PMC8729149

[6] Tomson B.N. , Arndt K.M. The many roles of the conserved eukaryotic paf1 complex in regulating transcription, histone modifications, and disease states. Biochim. Biophys. Acta. 2013; 1829:116–126. 22982193 10.1016/j.bbagrm.2012.08.011 PMC3541448

[7] Jaehning J.A. The paf1 complex: platform or player in RNA polymerase II transcription. Biochim. Biophys. Acta. 2010; 1799:379–388. 20060942 10.1016/j.bbagrm.2010.01.001 PMC2862274

[8] Hanks S. , Perdeaux E.R., Seal S., Ruark E., Mahamdallie S.S., Murray A., Ramsay E., Del Vecchio Duarte S., Zachariou A., de Souza B. et al. Germline mutations in the PAF1 complex gene CTR9 predispose to wilms tumour. Nat. Commun. 2014; 5:4398. 25099282 10.1038/ncomms5398 PMC4143912

[9] Zeng H. , Xu W. Ctr9, a key subunit of PAFc, affects global estrogen signaling and drives ERalpha-positive breast tumorigenesis. Genes Dev. 2015; 29:2153–2167. 26494790 10.1101/gad.268722.115 PMC4617979

[10] Zeng H. , Lu L., Chan N.T., Horswill M., Ahlquist P., Zhong X., Xu W. Systematic identification of ctr9 regulome in ERalpha-positive breast cancer. BMC Genomics. 2016; 17:902. 27829357 10.1186/s12864-016-3248-3 PMC5103509

[11] Yu J.R. , Lee C.H., Oksuz O., Stafford J.M., Reinberg D PRC2 is high maintenance. Genes Dev. 2019; 33:903–935. 31123062 10.1101/gad.325050.119 PMC6672058

[12] Holoch D. , Margueron R. Mechanisms regulating PRC2 recruitment and enzymatic activity. Trends Biochem. Sci. 2017; 42:531–542. 28483375 10.1016/j.tibs.2017.04.003

[13] van Mierlo G. , Veenstra G.J.C., Vermeulen M., Marks H. The complexity of PRC2 subcomplexes. Trends Cell Biol. 2019; 29:660–671. 31178244 10.1016/j.tcb.2019.05.004

[14] Antonysamy S. , Condon B., Druzina Z., Bonanno J.B., Gheyi T., Zhang F., MacEwan I., Zhang A., Ashok S., Rodgers L. et al. Structural context of disease-associated mutations and putative mechanism of autoinhibition revealed by X-ray crystallographic analysis of the EZH2-SET domain. PLoS One. 2013; 8:e84147. 24367637 10.1371/journal.pone.0084147 PMC3868555

[15] Cao R. , Zhang Y. SUZ12 is required for both the histone methyltransferase activity and the silencing function of the EED-EZH2 complex. Mol. Cell. 2004; 15:57–67. 15225548 10.1016/j.molcel.2004.06.020

[16] Wu H. , Zeng H., Dong A., Li F., He H., Senisterra G., Seitova A., Duan S., Brown P.J., Vedadi M. et al. Structure of the catalytic domain of EZH2 reveals conformational plasticity in cofactor and substrate binding sites and explains oncogenic mutations. PLoS One. 2013; 8:e83737. 24367611 10.1371/journal.pone.0083737 PMC3868588

[17] Lee C.H. , Yu J.R., Kumar S., Jin Y., LeRoy G., Bhanu N., Kaneko S., Garcia B.A., Hamilton A.D., Reinberg D Allosteric activation dictates PRC2 activity independent of its recruitment to chromatin. Mol. Cell. 2018; 70:422–434. 29681499 10.1016/j.molcel.2018.03.020 PMC5935545

[18] Lee C.H. , Yu J.R., Granat J., Saldana-Meyer R., Andrade J., LeRoy G., Jin Y., Lund P., Stafford J.M., Garcia B.A. et al. Automethylation of PRC2 promotes H3K27 methylation and is impaired in H3K27M pediatric glioma. Genes Dev. 2019; 33:1428–1440. 31488577 10.1101/gad.328773.119 PMC6771381

[19] Smits A.H. , Jansen P.W., Poser I., Hyman A.A., Vermeulen M. Stoichiometry of chromatin-associated protein complexes revealed by label-free quantitative mass spectrometry-based proteomics. Nucleic Acids Res. 2013; 41:e28. 23066101 10.1093/nar/gks941 PMC3592467

[20] Kasinath V. , Faini M., Poepsel S., Reif D., Feng X.A., Stjepanovic G., Aebersold R., Nogales E. Structures of human PRC2 with its cofactors AEBP2 and JARID2. Science. 2018; 359:940–944. 29348366 10.1126/science.aar5700 PMC5840869

[21] Li H. , Liefke R., Jiang J., Kurland J.V., Tian W., Deng P., Zhang W., He Q., Patel D.J., Bulyk M.L. et al. Polycomb-like proteins link the PRC2 complex to CpG islands. Nature. 2017; 549:287–291. 28869966 10.1038/nature23881 PMC5937281

[22] Oksuz O. , Narendra V., Lee C.H., Descostes N., LeRoy G., Raviram R., Blumenberg L., Karch K., Rocha P.P., Garcia B.A. et al. Capturing the onset of PRC2-Mediated repressive domain formation. Mol. Cell. 2018; 70:1149–1162. 29932905 10.1016/j.molcel.2018.05.023 PMC7700016

[23] Conway E. , Jerman E., Healy E., Ito S., Holoch D., Oliviero G., Deevy O., Glancy E., Fitzpatrick D.J., Mucha M. et al. A family of vertebrate-specific polycombs encoded by the LCOR/LCORL genes balance PRC2 subtype activities. Mol. Cell. 2018; 70:408–421. 29628311 10.1016/j.molcel.2018.03.005

[24] Conway E. , Healy E., Bracken A.P. PRC2 mediated H3K27 methylations in cellular identity and cancer. Curr. Opin. Cell Biol. 2015; 37:42–48. 26497635 10.1016/j.ceb.2015.10.003

[25] Kim K.H. , Roberts C.W. Targeting EZH2 in cancer. Nat. Med. 2016; 22:128–134. 26845405 10.1038/nm.4036 PMC4918227

[26] Hoy S.M. Tazemetostat: first approval. Drugs. 2020; 80:513–521. 32166598 10.1007/s40265-020-01288-x

[27] Grosselin K. , Durand A., Marsolier J., Poitou A., Marangoni E., Nemati F., Dahmani A., Lameiras S., Reyal F., Frenoy O. et al. High-throughput single-cell chip-seq identifies heterogeneity of chromatin states in breast cancer. Nat. Genet. 2019; 51:1060–1066. 31152164 10.1038/s41588-019-0424-9

[28] Kagey M.H. , Newman J.J., Bilodeau S., Zhan Y., Orlando D.A., van Berkum N.L., Ebmeier C.C., Goossens J., Rahl P.B., Levine S.S. et al. Mediator and cohesin connect gene expression and chromatin architecture. Nature. 2010; 467:430–435. 20720539 10.1038/nature09380 PMC2953795

[29] Beringer M. , Pisano P., Di Carlo V., Blanco E., Chammas P., Vizan P., Gutierrez A., Aranda S., Payer B., Wierer M. et al. EPOP functionally links elongin and polycomb in pluripotent stem cells. Mol. Cell. 2016; 64:645–658. 27863225 10.1016/j.molcel.2016.10.018

[30] Langmead B. , Salzberg S.L. Fast gapped-read alignment with bowtie 2. Nat. Methods. 2012; 9:357–359. 22388286 10.1038/nmeth.1923 PMC3322381

[31] Ramirez F. , Ryan D.P., Gruning B., Bhardwaj V., Kilpert F., Richter A.S., Heyne S., Dundar F., Manke T. deepTools2: a next generation web server for deep-sequencing data analysis. Nucleic Acids Res. 2016; 44:W160–W165. 27079975 10.1093/nar/gkw257 PMC4987876

[32] Zhang Y. , Liu T., Meyer C.A., Eeckhoute J., Johnson D.S., Bernstein B.E., Nusbaum C., Myers R.M., Brown M., Li W. et al. Model-based analysis of chip-Seq (MACS). Genome Biol. 2008; 9:R137. 18798982 10.1186/gb-2008-9-9-r137 PMC2592715

[33] Ciriello G. , Gatza M.L., Beck A.H., Wilkerson M.D., Rhie S.K., Pastore A., Zhang H., McLellan M., Yau C., Kandoth C. et al. Comprehensive molecular portraits of invasive lobular breast cancer. Cell. 2015; 163:506–519. 26451490 10.1016/j.cell.2015.09.033 PMC4603750

[34] Yu G. , Wang L.G., He Q.Y. ChIPseeker: an R/Bioconductor package for ChIP peak annotation, comparison and visualization. Bioinformatics. 2015; 31:2382–2383. 25765347 10.1093/bioinformatics/btv145

[35] Wang Z. , Civelek M., Miller C.L., Sheffield N.C., Guertin M.J., Zang C. BART: a transcription factor prediction tool with query gene sets or epigenomic profiles. Bioinformatics. 2018; 34:2867–2869. 29608647 10.1093/bioinformatics/bty194 PMC6084568

[36] Campbell T.M. , Castro M.A.A., de Oliveira K.G., Ponder B.A.J., Meyer K.B. ERalpha binding by transcription factors NFIB and YBX1 enables FGFR2 signaling to modulate estrogen responsiveness in breast cancer. Cancer Res. 2018; 78:410–421. 29180470 10.1158/0008-5472.CAN-17-1153 PMC5774586

[37] Reddy B.Y. , Greco S.J., Patel P.S., Trzaska K.A., Rameshwar P. RE-1-silencing transcription factor shows tumor-suppressor functions and negatively regulates the oncogenic TAC1 in breast cancer cells. Proc. Natl. Acad. Sci. USA. 2009; 106:4408–4413. 19246391 10.1073/pnas.0809130106 PMC2647978

[38] Ai B. , Kong X., Wang X., Zhang K., Yang X., Zhai J., Gao R., Qi Y., Wang J., Wang Z. et al. LINC01355 suppresses breast cancer growth through FOXO3-mediated transcriptional repression of CCND1. Cell Death Dis. 2019; 10:502. 31243265 10.1038/s41419-019-1741-8 PMC6594972

[39] Litchfield L.M. , Riggs K.A., Hockenberry A.M., Oliver L.D., Barnhart K.G., Cai J., Pierce W.M. Jr, Ivanova M.M., Bates P.J., Appana S.N. et al. Identification and characterization of nucleolin as a COUP-TFII coactivator of retinoic acid receptor beta transcription in breast cancer cells. PLoS One. 2012; 7:e38278. 22693611 10.1371/journal.pone.0038278 PMC3365040

[40] Healy E. , Mucha M., Glancy E., Fitzpatrick D.J., Conway E., Neikes H.K., Monger C., Van Mierlo G., Baltissen M.P., Koseki Y. et al. PRC2.1 and PRC2.2 synergize to coordinate H3K27 trimethylation. Mol. Cell. 2019; 76:437–452. 31521505 10.1016/j.molcel.2019.08.012

[41] Hauri S. , Comoglio F., Seimiya M., Gerstung M., Glatter T., Hansen K., Aebersold R., Paro R., Gstaiger M., Beisel C. A high-density map for navigating the human polycomb complexome. Cell Rep. 2016; 17:583–595. 27705803 10.1016/j.celrep.2016.08.096

[42] Oliviero G. , Brien G.L., Waston A., Streubel G., Jerman E., Andrews D., Doyle B., Munawar N., Wynne K., Crean J. et al. Dynamic protein interactions of the polycomb repressive complex 2 during differentiation of pluripotent cells. Mol. Cell. Proteom. 2016; 15:3450–3460. 10.1074/mcp.M116.062240 PMC5098042 27634302

[43] Alekseyenko A.A. , Gorchakov A.A., Kharchenko P.V., Kuroda M.I. Reciprocal interactions of human C10orf12 and C17orf96 with PRC2 revealed by BioTAP-XL cross-linking and affinity purification. Proc. Natl. Acad. Sci. USA. 2014; 111:2488–2493. 24550272 10.1073/pnas.1400648111 PMC3932854

[44] Grijzenhout A. , Godwin J., Koseki H., Gdula M.R., Szumska D., McGouran J.F., Bhattacharya S., Kessler B.M., Brockdorff N., Cooper S. Functional analysis of AEBP2, a PRC2 polycomb protein, reveals a trithorax phenotype in embryonic development and in ESCs. Development. 2016; 143:2716–2723. 27317809 10.1242/dev.123935 PMC5004903

[45] Jain P. , Ballare C., Blanco E., Vizan P., Di Croce L. PHF19 mediated regulation of proliferation and invasiveness in prostate cancer cells. Elife. 2020; 9:e51373. 32155117 10.7554/eLife.51373 PMC7064337

[46] McIntyre G.J. , Arndt A.J., Gillespie K.M., Mak W.M., Fanning G.C. A comparison of multiple shRNA expression methods for combinatorial RNAi. Genet. Vaccines Ther. 2011; 9:9. 21496330 10.1186/1479-0556-9-9 PMC3098768

[47] Yan Q. , Xu K., Xing J., Zhang T., Wang X., Wei Z., Ren C., Liu Z., Shao S., Zhang Z. Multiplex CRISPR/Cas9-based genome engineering enhanced by Drosha-mediated sgRNA-shRNA structure. Sci. Rep. 2016; 6:38970. 27941919 10.1038/srep38970 PMC5150520

[48] Liu Z. , Chen O., Wall J.B.J., Zheng M., Zhou Y., Wang L., Vaseghi H.R., Qian L., Liu J. Systematic comparison of 2A peptides for cloning multi-genes in a polycistronic vector. Sci. Rep. 2017; 7:2193. 28526819 10.1038/s41598-017-02460-2 PMC5438344

[49] Czudnochowski N. , Bosken C.A., Geyer M. Serine-7 but not serine-5 phosphorylation primes RNA polymerase II CTD for P-TEFb recognition. Nat. Commun. 2012; 3:842. 22588304 10.1038/ncomms1846

[50] Petrenko N. , Jin Y., Dong L., Wong K.H., Struhl K. Requirements for RNA polymerase II preinitiation complex formation in vivo. Elife. 2019; 8:e43654. 30681409 10.7554/eLife.43654 PMC6366898

[51] Schilbach S. , Hantsche M., Tegunov D., Dienemann C., Wigge C., Urlaub H., Cramer P. Structures of transcription pre-initiation complex with TFIIH and mediator. Nature. 2017; 551:204–209. 29088706 10.1038/nature24282 PMC6078178

[52] Bowman E.A. , Kelly W.G. RNA polymerase II transcription elongation and pol II CTD ser2 phosphorylation: a tail of two kinases. Nucleus. 2014; 5:224–236. 24879308 10.4161/nucl.29347 PMC4133218

[53] Moorefield B. SPT5 roles in transcriptional elongation. Nat. Struct. Mol. Biol. 2021; 28:778. 34608338 10.1038/s41594-021-00673-8

[54] Yu Y. , Qi J., Xiong J., Jiang L., Cui D., He J., Chen P., Li L., Wu C., Ma T. et al. Epigenetic co-deregulation of EZH2/TET1 is a senescence-countering, actionable vulnerability in triple-negative breast cancer. Theranostics. 2019; 9:761–777. 30809307 10.7150/thno.29520 PMC6376470

[55] Konze K.D. , Ma A., Li F., Barsyte-Lovejoy D., Parton T., Macnevin C.J., Liu F., Gao C., Huang X.P., Kuznetsova E. et al. An orally bioavailable chemical probe of the lysine methyltransferases EZH2 and EZH1. ACS Chem. Biol. 2013; 8:1324–1334. 23614352 10.1021/cb400133j PMC3773059

[56] Qi W. , Zhao K., Gu J., Huang Y., Wang Y., Zhang H., Zhang M., Zhang J., Yu Z., Li L. et al. An allosteric PRC2 inhibitor targeting the H3K27me3 binding pocket of EED. Nat. Chem. Biol. 2017; 13:381–388. 28135235 10.1038/nchembio.2304

[57] Verma S.K. , Tian X., LaFrance L.V., Duquenne C., Suarez D.P., Newlander K.A., Romeril S.P., Burgess J.L., Grant S.W., Brackley J.A. et al. Identification of potent, selective, cell-active inhibitors of the histone lysine methyltransferase EZH2. ACS Med. Chem. Lett. 2012; 3:1091–1096. 24900432 10.1021/ml3003346 PMC4025676

[58] Chen S.L. , Loffler K.A., Chen D., Stallcup M.R., Muscat G.E. The coactivator-associated arginine methyltransferase is necessary for muscle differentiation: CARM1 coactivates myocyte enhancer factor-2. J. Biol. Chem. 2002; 277:4324–4333. 11713257 10.1074/jbc.M109835200

[59] Massoni-Laporte A. , Perrot M., Ponger L., Boucherie H., Guieysse-Peugeot A.L. Proteome analysis of a CTR9 deficient yeast strain suggests that ctr9 has function(s) independent of the paf1 complex. Biochim. Biophys. Acta. 2012; 1824:759–768. 22446411 10.1016/j.bbapap.2012.02.010

[60] Chaturvedi D. , Inaba M., Scoggin S., Buszczak M. Drosophila CG2469 encodes a homolog of human CTR9 and is essential for development. G3 (Bethesda). 2016; 6:3849–3857. 27678520 10.1534/g3.116.035196 PMC5144956

[61] Akanuma T. , Koshida S., Kawamura A., Kishimoto Y., Takada S. Paf1 complex homologues are required for Notch-regulated transcription during somite segmentation. EMBO Rep. 2007; 8:858–863. 17721442 10.1038/sj.embor.7401045 PMC1973952

[62] Zhang K. , Haversat J.M., Mager J. CTR9/PAF1c regulates molecular lineage identity, histone H3K36 trimethylation and genomic imprinting during preimplantation development. Dev. Biol. 2013; 383:15–27. 24036311 10.1016/j.ydbio.2013.09.005 PMC4903072

[63] Pasini D. , Bracken A.P., Jensen M.R., Lazzerini Denchi E., Helin K. Suz12 is essential for mouse development and for EZH2 histone methyltransferase activity. EMBO J. 2004; 23:4061–4071. 15385962 10.1038/sj.emboj.7600402 PMC524339

[64] Streubel G. , Watson A., Jammula S.G., Scelfo A., Fitzpatrick D.J., Oliviero G., McCole R., Conway E., Glancy E., Negri G.L. et al. The H3K36me2 methyltransferase nsd1 demarcates PRC2-Mediated H3K27me2 and H3K27me3 domains in embryonic stem cells. Mol. Cell. 2018; 70:371–379. 29606589 10.1016/j.molcel.2018.02.027

[65] Riising E.M. , Comet I., Leblanc B., Wu X., Johansen J.V., Helin K. Gene silencing triggers polycomb repressive complex 2 recruitment to CpG islands genome wide. Mol. Cell. 2014; 55:347–360. 24999238 10.1016/j.molcel.2014.06.005

[66] Hosogane M. , Funayama R., Shirota M., Nakayama K. Lack of transcription triggers H3K27me3 accumulation in the gene body. Cell Rep. 2016; 16:696–706. 27396330 10.1016/j.celrep.2016.06.034

[67] Laugesen A. , Hojfeldt J.W., Helin K. Molecular mechanisms directing PRC2 recruitment and H3K27 methylation. Mol. Cell. 2019; 74:8–18. 30951652 10.1016/j.molcel.2019.03.011 PMC6452890

[68] Ballare C. , Lange M., Lapinaite A., Martin G.M., Morey L., Pascual G., Liefke R., Simon B., Shi Y., Gozani O. et al. Phf19 links methylated lys36 of histone H3 to regulation of polycomb activity. Nat. Struct. Mol. Biol. 2012; 19:1257–1265. 23104054 10.1038/nsmb.2434 PMC3926938

[69] Schmitges F.W. , Prusty A.B., Faty M., Stutzer A., Lingaraju G.M., Aiwazian J., Sack R., Hess D., Li L., Zhou S. et al. Histone methylation by PRC2 is inhibited by active chromatin marks. Mol. Cell. 2011; 42:330–341. 21549310 10.1016/j.molcel.2011.03.025

[70] Peng J.C. , Valouev A., Swigut T., Zhang J., Zhao Y., Sidow A., Wysocka J. Jarid2/Jumonji coordinates control of PRC2 enzymatic activity and target gene occupancy in pluripotent cells. Cell. 2009; 139:1290–1302. 20064375 10.1016/j.cell.2009.12.002 PMC2911953

[71] Youmans D.T. , Gooding A.R., Dowell R.D., Cech T.R. Competition between PRC2.1 and 2.2 subcomplexes regulates PRC2 chromatin occupancy in human stem cells. Mol. Cell. 2021; 81:488–501. 33338397 10.1016/j.molcel.2020.11.044 PMC7867654

[72] Sanulli S. , Justin N., Teissandier A., Ancelin K., Portoso M., Caron M., Michaud A., Lombard B., da Rocha S.T., Offer J. et al. Jarid2 methylation via the PRC2 complex regulates H3K27me3 deposition during cell differentiation. Mol. Cell. 2015; 57:769–783. 25620564 10.1016/j.molcel.2014.12.020 PMC4352895

[73] Perino M. , van Mierlo G., Karemaker I.D., van Genesen S., Vermeulen M., Marks H., van Heeringen S.J., Veenstra G.J.C. MTF2 recruits polycomb repressive complex 2 by helical-shape-selective DNA binding. Nat. Genet. 2018; 50:1002–1010. 29808031 10.1038/s41588-018-0134-8

[74] Xu B. , Konze K.D., Jin J., Wang G.G. Targeting EZH2 and PRC2 dependence as novel anticancer therapy. Exp. Hematol. 2015; 43:698–712. 26027790 10.1016/j.exphem.2015.05.001 PMC4706459

[75] Ezponda T. , Dupere-Richer D., Will C.M., Small E.C., Varghese N., Patel T., Nabet B., Popovic R., Oyer J., Bulic M. et al. UTX/KDM6A loss enhances the malignant phenotype of multiple myeloma and sensitizes cells to EZH2 inhibition. Cell Rep. 2017; 21:628–640. 29045832 10.1016/j.celrep.2017.09.078 PMC5706555

[76] Van der Meulen J. , Sanghvi V., Mavrakis K., Durinck K., Fang F., Matthijssens F., Rondou P., Rosen M., Pieters T., Vandenberghe P. et al. The H3K27me3 demethylase UTX is a gender-specific tumor suppressor in T-cell acute lymphoblastic leukemia. Blood. 2015; 125:13–21. 25320243 10.1182/blood-2014-05-577270 PMC4347284

[77] Italiano A. , Soria J.C., Toulmonde M., Michot J.M., Lucchesi C., Varga A., Coindre J.M., Blakemore S.J., Clawson A., Suttle B. et al. Tazemetostat, an EZH2 inhibitor, in relapsed or refractory B-cell non-Hodgkin lymphoma and advanced solid tumours: a first-in-human, open-label, phase 1 study. Lancet Oncol. 2018; 19:649–659. 29650362 10.1016/S1470-2045(18)30145-1

[78] Moore H.M. , Gonzalez M.E., Toy K.A., Cimino-Mathews A., Argani P., Kleer C.G. EZH2 inhibition decreases p38 signaling and suppresses breast cancer motility and metastasis. Breast Cancer Res. Treat. 2013; 138:741–752. 23539298 10.1007/s10549-013-2498-x PMC3690767

