# Supplementary material for: The transcriptional elongation factor CTR9 demarcates PRC2-mediated H3K27me3 domains by altering PRC2 subtype equilibrium
Source: Nucleic Acids Res. 2022 Feb 7;50(4):1969–92. doi: 10.1093/nar/gkac047 (PMC8887485; doi:10.1093/nar/gkac047)
Supplement: gkac047_Supplemental_Files [file gkac047_supplemental_files.zip › Supplemental Materials - NAR 2021-2nd revision-Final.pdf]

# Supplemental Material

**The transcriptional elongation factor CTR9 demarcates PRC2-mediated H3K27me3 domains by altering PRC2 subtype equilibrium**

Chan et al., 2021

**Supplemental Figure S1**

**Supplemental Figure S2**

**Supplemental Figure S3**

**Supplemental Figure S4**

**Supplemental Figure S5**

**Supplemental Figure S6**

**Supplemental Figure S7**

**Supplemental Figure S8**

**Supplemental Figure S9**

**Supplemental Figure S10**

**Supplemental Figure S11**

**Supplemental Figure S12**

**Supplemental Table S1**

**Supplemental Table S2**

**Supplemental Table S3**

**Supplemental Table S4**

**Supplemental Table S5**

**Supplemental Table S6**

**Supplemental Table S7**

**Supplemental Table S8**

# Supplemental Figure S1

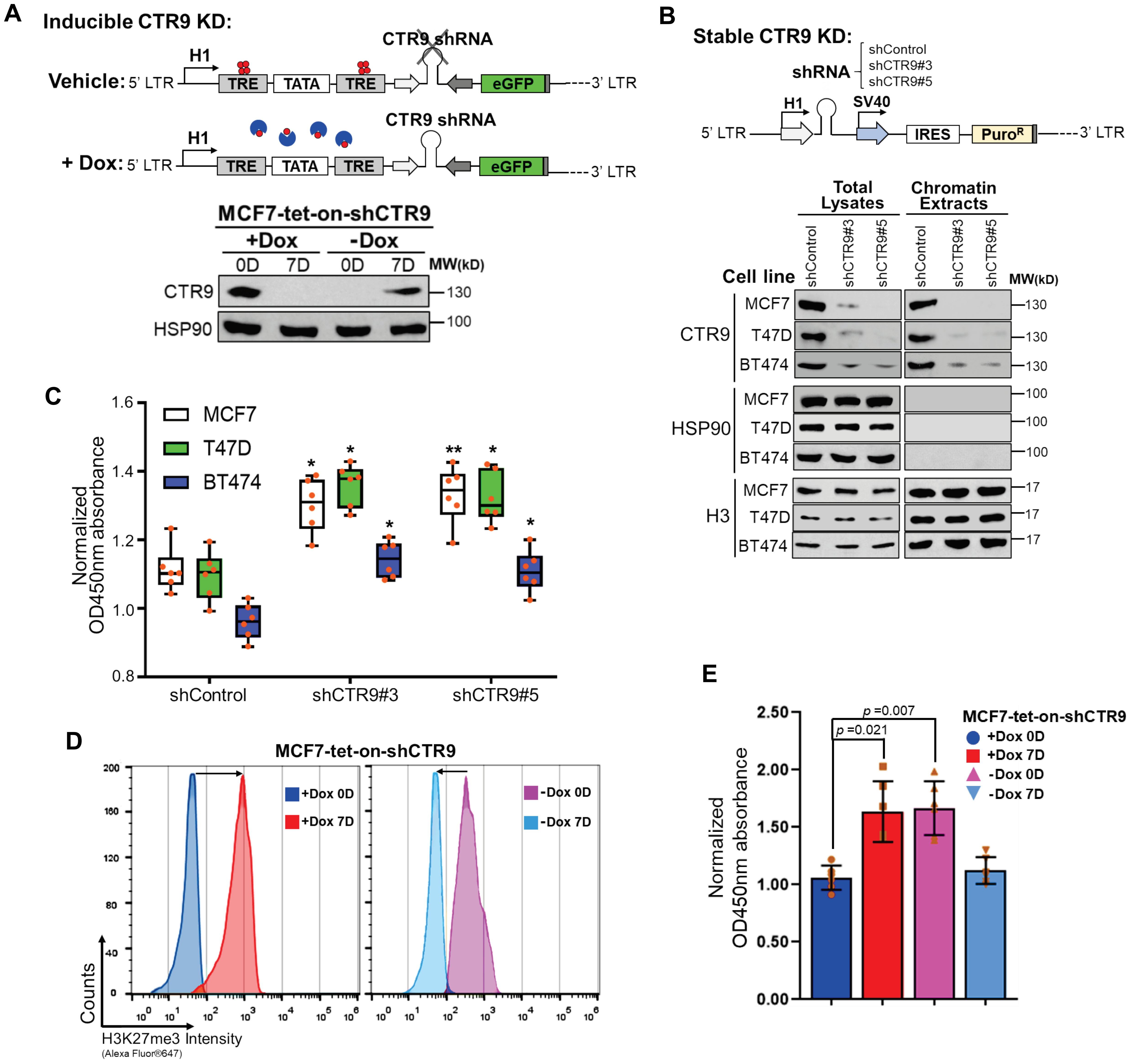

## Supplemental Figure S1. (Related to Figure 1)

**A.** Schematic design of doxycycline inducible CTR9 knockdown cell line MCF7-tet-on-shCTR9 (*Top*). Western blotting showed the depletion of CTR9 by Dox treatment for 7 days and recovery of CTR9 protein by removal of Dox after 7 days (*Bottom*).

**B.** Schematic design of the stable CTR9 knockdown using shCTR9#3 and shCTR9#5 (*Top*). Western blotting results showed the levels of CTR9 in total lysates and chromatin extracts in MCF7, T47D or BT474 cell lines stably expressing shCTR9#3 and ShCTR9#5 (*Bottom*).

**C.** H3K27me3 levels measured by ELISA assays in MCF7, T47D and BT474 cells expressing shControl, shCTR9#3 or shCTR9#5. Data were normalized to the respective total histone H3 levels and represented as mean ± SD (n = 6). P-values were calculated using two tails t-test with Welch's correction (*: $p$ <0.05; **: $p$ <0.01).

**D.** Flow cytometry quantification of H3K27me3 intensity in MCF7-tet-on–shCTR9 cells upon addition and removal of Dox for 0 and 7 days.

**E.** H3K27me3 levels measured by ELISA assays in MCF7-tet-on–shCTR9 upon addition and removal of Dox. Data were normalized to the respective total histone H3 levels and represented as mean ± SD (n = 6). P-values were calculated using two tails t-test with Welch's correction.

# Supplemental Figure S2

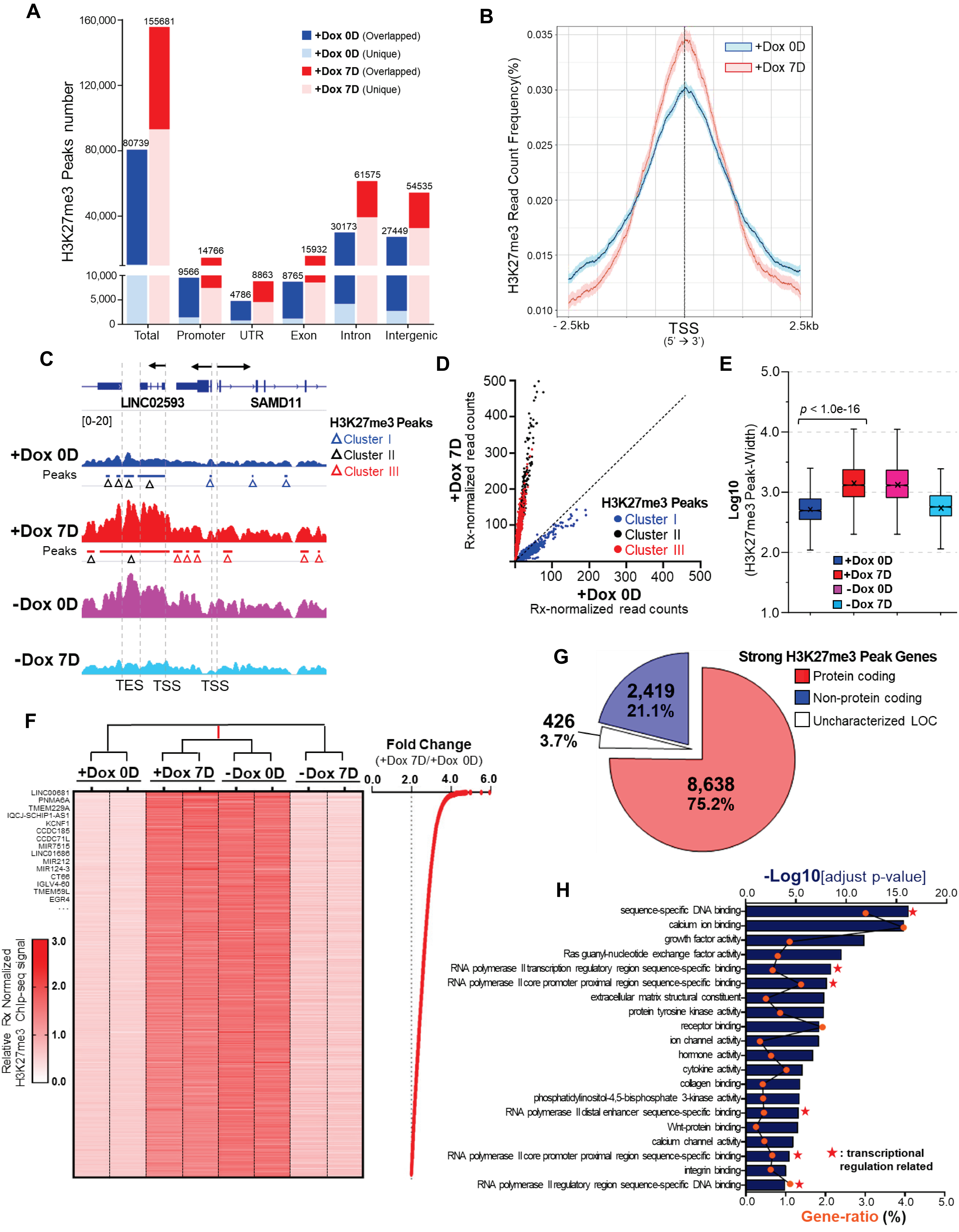

## Supplemental Figure S2. (Related to Figure 2)

**A.** Respective genome-wide distribution of the overlapped (Light Blue) and unique (Blue) H3K27me3 peak numbers in MCF7-tet-on-shCTR9 cells treated with Dox for 0 day. After 7-day treatment with Dox, the overlapped (Light Red) and unique H3K27me3 peaks (Red) were shown. Total peak numbers for each group and their distribution to promoter, UTR, exon, intron and intergenic regions were indicated.

**B.** Continuous read count frequency (%) of H3K27me3 peaks distributed around the TSS regions (TSS ± 2.5kb).

**C.** Representative genome-browser snapshot of Rx-normalized H3K27me3 ChIP-Rx signals in MCF7-tet-on-shCTR9 cells under indicated treatment conditions. Each signal-track represents the mean of two biological replicates. Peaks classified to three clusters were denoted with blue, red, or black triangles, respectively.

**D.** Dot plot of Rx-normalized read counts of H3K27me3 peaks from MCF7-tet-on-shCTR9 cells treated with Dox for 0 day and 7 days. Peaks from three clusters were represented with blue, black and red dots, respectively.

**E.** Notched box plot showing the peak width of H3K27me3 from MCF7-tet-on-shCTR9 cells upon addition or removal of Dox for 0 day or 7 days. P-value of Welch's t-test was calculated.

**F.** Heatmap of the relative Rx-normalized H3K27me3 ChIP-Rx profiles (n=2) at strong H3K27me3 peak genes (*Left*). The Dox-induced H3K27me3 peak fold-changes were plotted (*Right*).

**G.** Genomic distribution of the strong H3K27me3 peak genes (11,483).

**H.** Gene Ontology pathway analysis (Molecular Function) of 11,483 strong H3K27me3 peak genes.

# Supplemental Figure S3

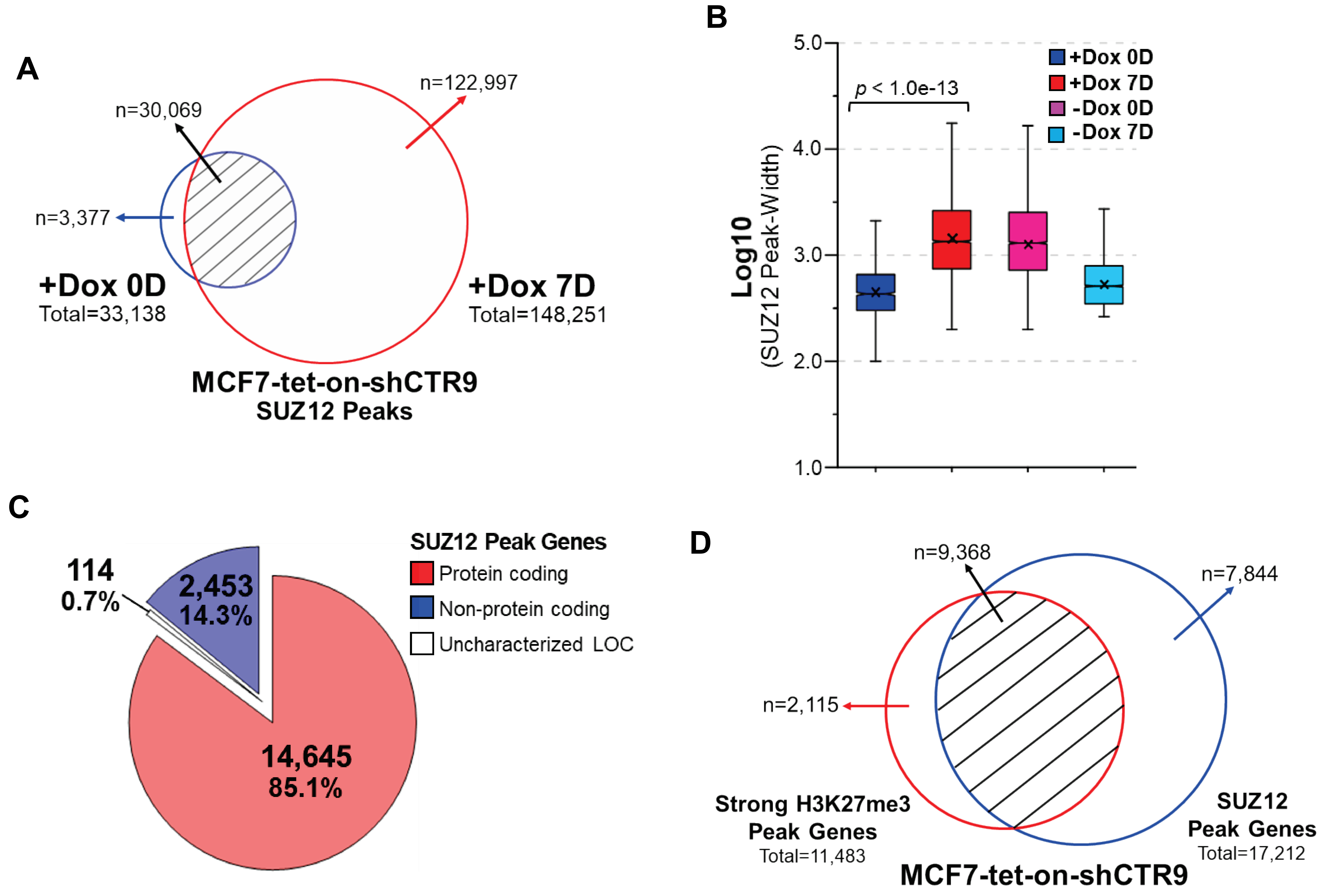


## Supplemental Figure S3. (Related to Figure 3)

**A.** Venn Diagram of the SUZ12 peaks between '+Dox 0D' and '+Dox 7D' groups in MCF7-tet-on-shCTR9 cells.

**B.** Notched box plot showing the peak width of SUZ12 from MCF7-tet-on-shCTR9 cells upon the addition or removal of Dox for 0 day or 7 days. P-value of Welch's t-test was calculated.

**C.** Genomic distribution of 17,212 SUZ12 peak genes

**D.** Venn Diagram showing a large overlap between the strong H3K27me3 peak genes and SUZ12 peak genes.

# Supplemental Figure S4

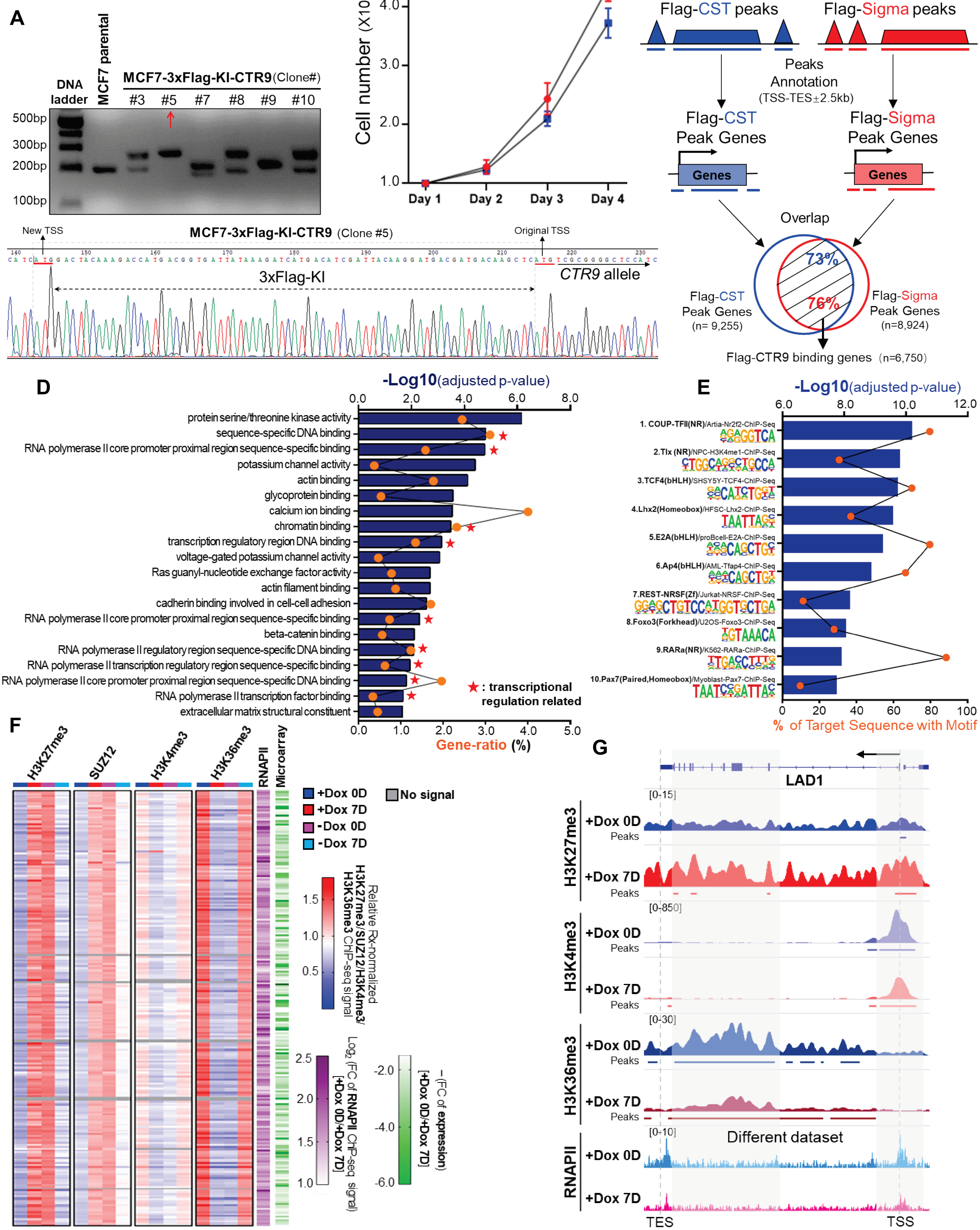

## Supplemental Figure S4. (Related to Figure 4)

**A.** Selection of homozygous Flag knock-in (KI) clones using PCR identified clone #5 as a correctly engineered MCF7-3xFlag-KI-CTR9 clone (*Top*, TSS: translation start site). This was verified by sequencing (*Bottom*).

**B.** Measuring four-day proliferation by cell counting in MCF7-3xFlag-KI-CTR9 and parental MCF7 cells. Data are represented as mean±SD (n=6).

**C.** Workflow for identifying authentic Flag-CTR9 binding genes. Flag-CTR9 peak genes annotated from ChIP-Rx peaks using two commercial Flag antibodies (CST: Cell Signaling Technology, Sigma: Sigma Aldrich) were overlapped. Flag-CTR9 peaks were annotated within the range of TSS-TES ± 2.5kb (*top*). Venn Diagram shows the Flag-CTR9 peak gene numbers by two Flag antibodies and the overlapped Flag-CTR9 peak genes (*bottom*).

**D.** Gene Ontology pathway analysis (Molecular Function) of 6,750 highly confident Flag-CTR9 binding genes. The top 20 identified pathways were sorted according to Bonferroni adjusted p-value followed by –log10 transformation.

**E.** Motif analysis of Flag-CTR9 binding genes (6,750). The top 10 discovered motifs were sorted according to Bonferroni adjusted p-value followed by –log10 transformation.

**F.** Heatmap of changes in H3K27me3, SUZ12, H3K4me3 and H3K36me3 ChIP-Rx signals on 240 CTR9 regulated genes in MCF7 cells (*left*). The corresponding log2 (fold changes) in RNAPII ChIP-seq signals as well as log2 (fold changes) in expression derived from our previously published ChIP-seq and Microarray datasets are shown (*right*).

**G.** Representative genome browser snapshot of normalized ChIP-Rx signals for H3K27me3, H3K4me3, H3K36me3 and previously analyzed RNAPII ChIP-seq signal on LAD1, a previously identified CTR9 regulated gene, in +Dox 0D and +Dox 7D conditions.

# Supplemental Figure S5

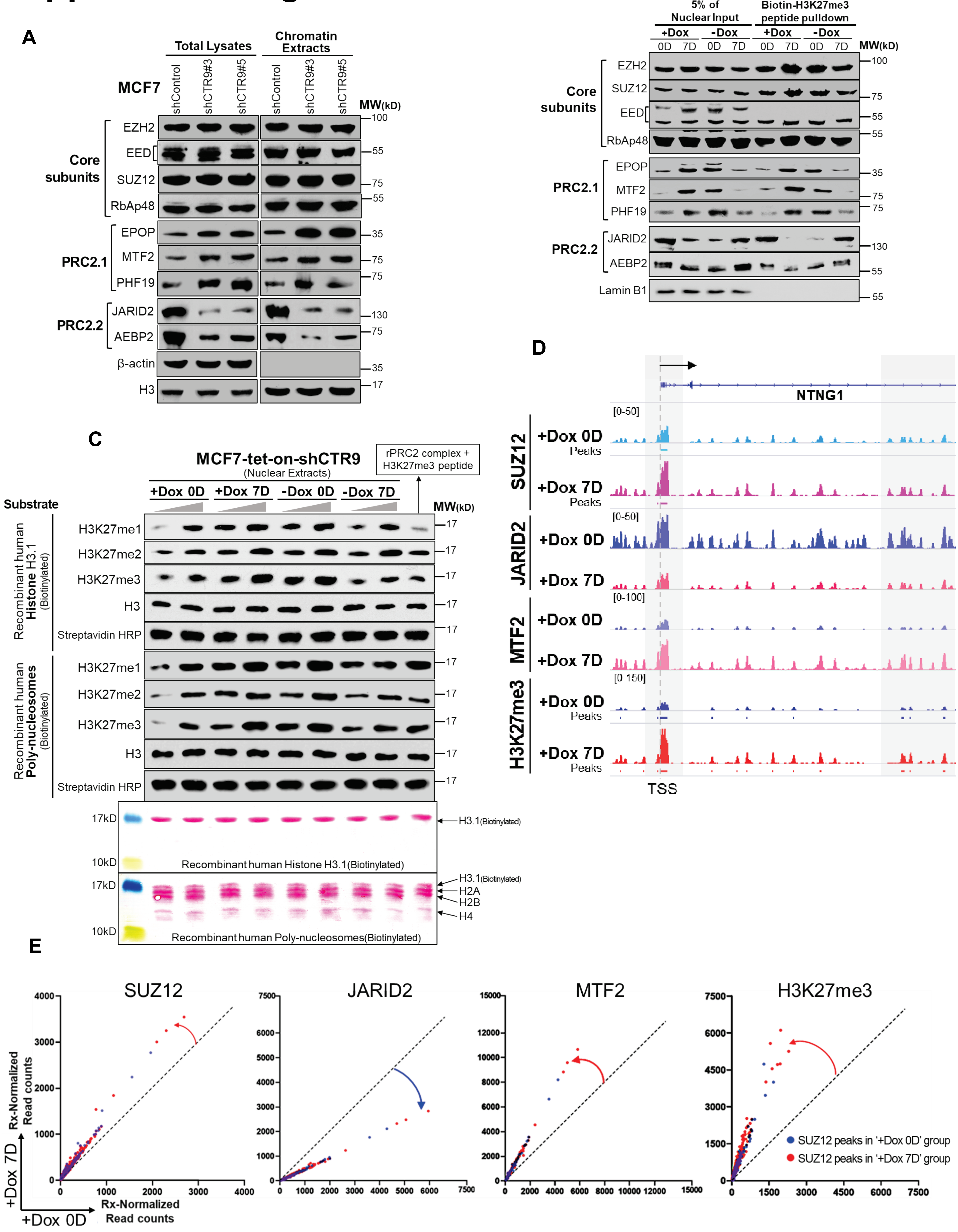

## Supplemental Figure S5. (Related to Figure 5)

**A.** Western blotting analysis of PRC2 core and facultative subunits in total lysates and chromatin fraction of MCF7-shControl/shCTR9#3/shCTR9#5 cells. β-actin and Histone H3 were used as loading controls for total lysates and chromatin extracts, respectively.

**B.** Western Blot analyses of PRC2 core and facultative subunits pulled down by biotinylated H3K27me3 peptide (aa 21-40) from nuclear extracts. Lamin B1 was used as a loading control for nuclear inputs.

**C.** *In vitro* H3K27 histone methyltransferase assay (HMT) using recombinant human biotinylated histone H3.1 or human biotinylated poly-nucleosomes as substrates. The ascending amounts of nuclear extracts from MCF7-tet-on-shCTR9 cells under indicated conditions were used as enzyme sources. Recombinant PRC2 complex and H3K27me3 peptide were used as a positive control (*Top*). Ponceau S staining of HMT substrates that pre-bound on streptavidin beads (*Bottom*).

**D.** Representative genome browser snapshot of ChIP-Rx signals for SUZ12, JARID2, MTF2, and H3K27me3 from MCF7-tet-on-shCTR9 cells treated with Dox for 0 day or 7 days. Each signal track represents the mean of two biological replicates.

**E.** Dot plots of Rx-normalized read counts on SUZ12 peaks from MCF7-tet-on-shCTR9 cells treated with Dox for 0 day or 7 days. Blue dots represent SUZ12 peaks identified in '+Dox 0D' group, and red dots represent SUZ12 peaks found in '+Dox 7D' group.

# Supplemental Figure S6

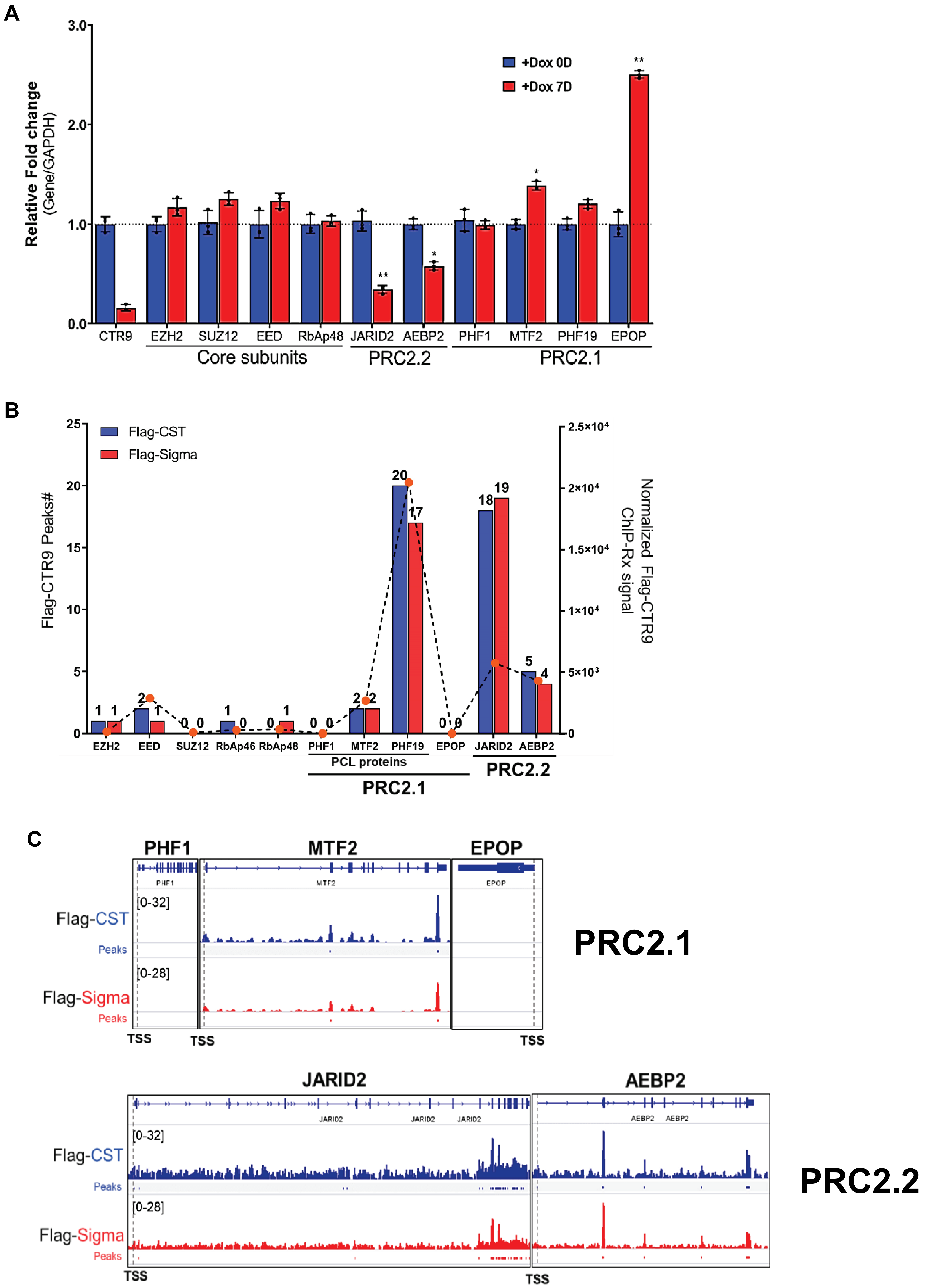

## Supplemental Figure S6. (Related to Figure 5)

**A.** RT-qPCR analysis of mRNA levels of PRC2 core and subtype accessory genes purified from total RNA in MCF7-tet-on–shCTR9 cells treated with Dox for 0 days or 7 days. Relative fold changes in mRNA levels are represented as the mean ± SD (n = 3). Total RNA was normalized to the internal control gene β-Actin. Values in the '+Dox 0D' group were set to 1. P-values were calculated using a paired two-tailed t-test *$p$<0.05, **$p$<0.01.

**B.** Bar plot summarizing the Flag-CTR9 peak numbers (left Y-axis) as well as ChIP-Rx signal (right Y-axis, normalized by gene length) on genes encoding PRC2 core subunits as well as PRC2 subtype specific proteins. (PRC2 core: EZH2, SUZ12, EED, RbAp46/48; PRC2.1: PHF1/MTF2/PHF19, EPOP; PRC2.2: JARID2, AEBP2)

**C.** Representative genome-browser snapshot of the averaged Flag (CST/Sigma) ChIP-Rx signals (n=2) in PRC2.1 (top) and PRC2.2 (bottom) subtype specific genes in MCF7-3xFlag-KI-CTR9 cells.

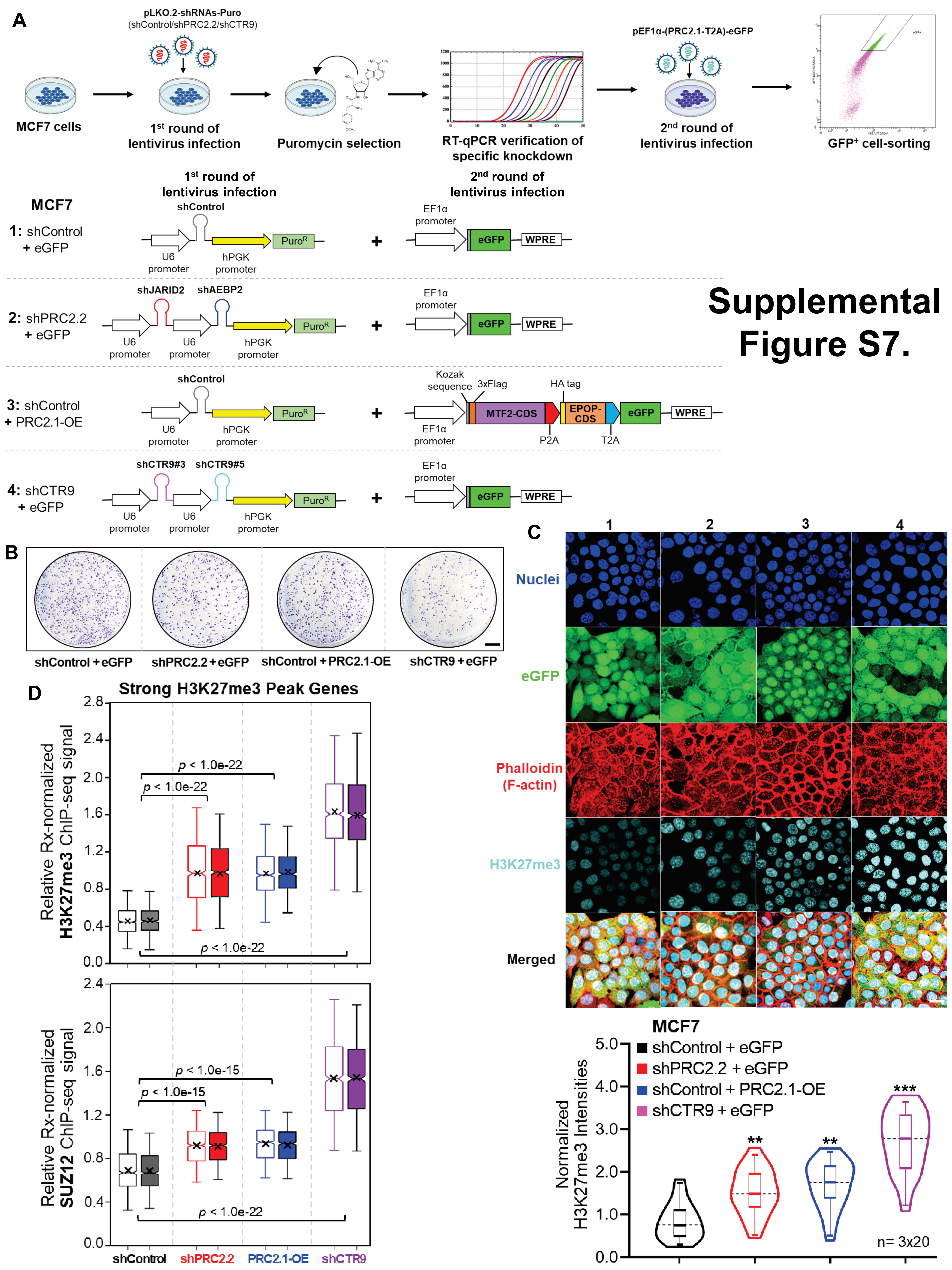

A
pLKO.2-shRNAs-Puro
(shControl/shPRC2.2/shCTR9)
MCF7 cells
1st round of lentivirus infection
Puromycin selection
RT-qPCR verification of specific knockdown
pEF1α-(PRC2.1-T2A)-eGFP
2nd round of lentivirus infection
GFP+ cell-sorting
MCF7
1st round of lentivirus infection
2nd round of lentivirus infection
1: shControl + eGFP
2: shPRC2.2 + eGFP
3: shControl + PRC2.1-OE
4: shCTR9 + eGFP
shControl
shJARID2
shAEBP2
shCTR9#3
shCTR9#5
U6 promoter
hPGK promoter
PuroR
EF1α promoter
eGFP
WPRE
Kozak sequence
3xFlag
HA tag
MTF2-CDS
EPOP-CDS
P2A
T2A
Supplemental Figure S7.
B
shControl + eGFP
shPRC2.2 + eGFP
shControl + PRC2.1-OE
shCTR9 + eGFP
C
1
2
3
4
Nuclei
eGFP
Phalloidin (F-actin)
H3K27me3
Merged
D
Strong H3K27me3 Peak Genes
Relative Rx-normalized H3K27me3 ChIP-seq signal
Relative Rx-normalized SUZ12 ChIP-seq signal
p < 1.0e-22
p < 1.0e-15
shControl
shPRC2.2
PRC2.1-OE
shCTR9
MCF7
shControl + eGFP
shPRC2.2 + eGFP
shControl + PRC2.1-OE
shCTR9 + eGFP
Normalized H3K27me3 Intensities
**
***
n= 3x20

## Supplemental Figure S7. (Related to Figure 6)

**A.** Schematic workflow for generating PRC2.2 knockdown (KD), PRC2.1 overexpression (OE) and CTR9 KD MCF7 cell lines. Two PRC2.2 subunits (JARID2/AEBP2) were knocked down or two PRC2.1 subunits (MTF2/EPOP) were overexpressed in MCF7 cells by sequentially transfecting cells with lentiviral plasmids containing duo-target shRNAs delivery system or 2A peptide mediated polycistronic overexpression system (Top), respectively. CTR9 was knocked down by infecting a lentiviral plasmid expressing two validated shRNAs. Detailed DNA elements and different combination of plasmids are shown in each group. shControl and eGFP alone served as background controls (Bottom).

**B.** Representative images of 2D colonies formation of MCF7 cells with Control KD, PRC2.2 KD, PRC2.1 OE and CTR9 KD. 4x scale bar shown at the bottom right applies to all images.

**C.** Representative images of Immuno-fluorescence staining of H3K27me3 (cyan), nuclei (blue), and F-actin (red) in Control KD (1), PRC2.2 KD (2), PRC2.1 OE (3) and CTR9 KD (4) MCF7 cells. eGFP (green) was served as a reporter control. 100x scale bar shown at the bottom right applies to all images (*Top*) .Ratios of H3K27me3 to nuclei staining intensity in 60 selected cells with complete nuclei from triplicateexperiments were plotted in violin integrated box plots. Difference in ratios were significant (*$p<0.05$; **$p<0.01$; ***$p<0.001$) by two-tailed t-test with Welch's correction (*Bottom*).

**D.** Notched box plot of relative Rx-normalized H3K27me3 (*top*) and SUZ12 (*bottom*) ChIP-seq signals at 11,483 strong H3K27me3 peak genes (identified in Fig. S2F) from Control KD, PRC2.2 KD, PRC2.1 OE and CTR9 KD MCF7 cells (n=2) . Paired Student's t test was used to calculate statistical significance among groups.

# Supplemental Figure S8

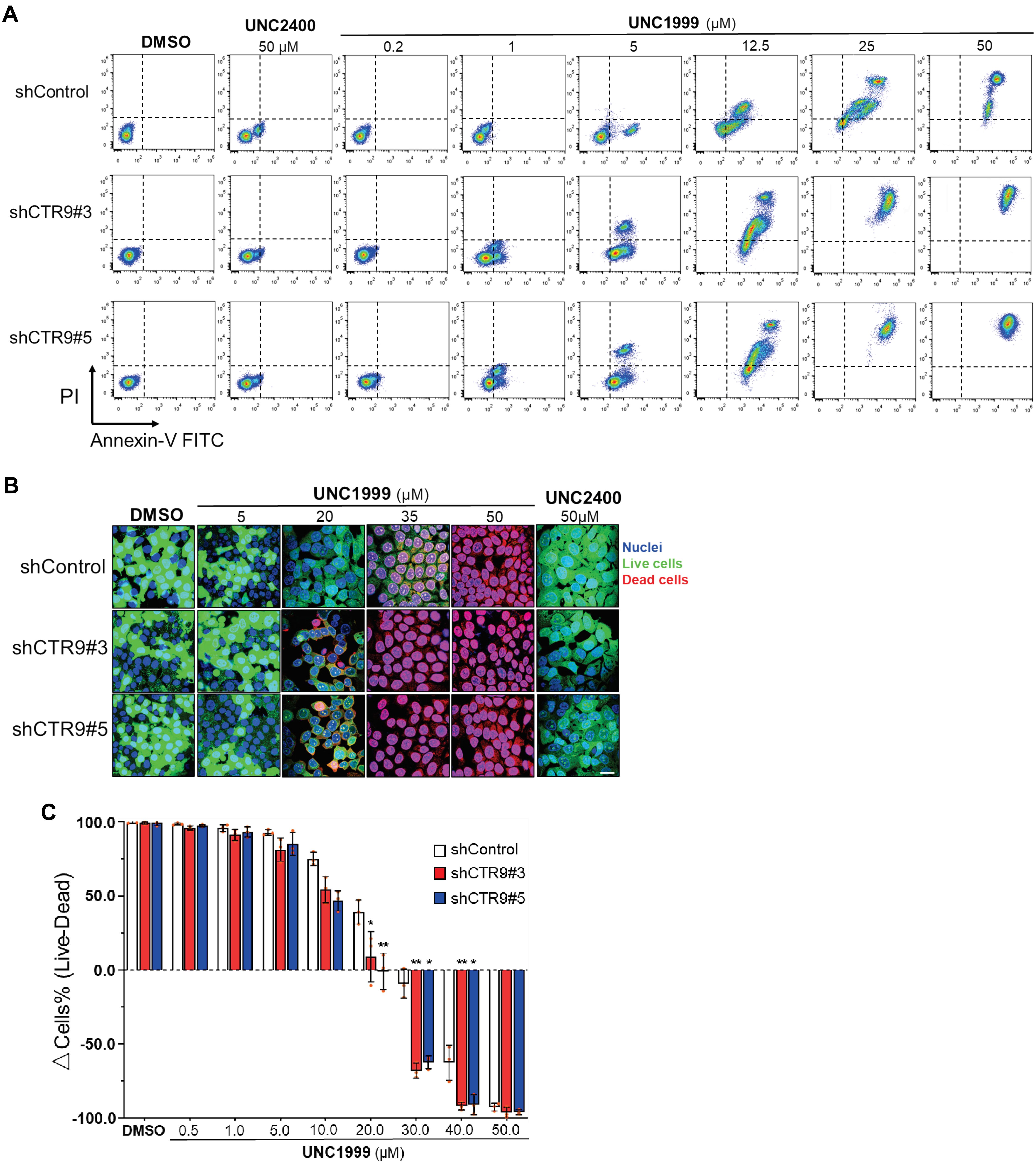

## Supplemental Figure S8. (Related to Figure 7)

**A.** Flow cytometry analyses of apoptotic and necrotic cells using PI uptake and annexin-V FITC labeling. MCF7-shControl or MCF7-shCTR9#3 or shCTR9#5 cells were treated with DMSO, 0.2 to 50 μM UNC1999, or 50 μM UNC2400 for 2 days.

**B.** Representative confocal images of MCF7-shControl, MCF7 shCTR9#3 or shCTR9#5 cells after treating with DMSO or the ascending concentrations of UNC1999 for 2 days. UNC2400 (negative paralog) serves as a negative control. Nuclei were stained in blue. Live cells with ubiquitous esterase activity were shown in green. Dead cells with impaired cell membrane were shown in red. 100x scale bar shown at the bottom right applies to all images.

**C.** Quantification of cytotoxicity in MCF7-shControl, MCF7shCTR9#3 or shCTR9#5 cells treated with ascending concentration of UNC1999. The differences between live cells and dead cells percentages were plotted. Comparative value between DMSO and each group of UNC1999 treatment were calculated and corresponding p-value of Welch's t-test were shown (*: $p$ <0.05; **: $p$ <0.01).

# Supplemental Figure S9

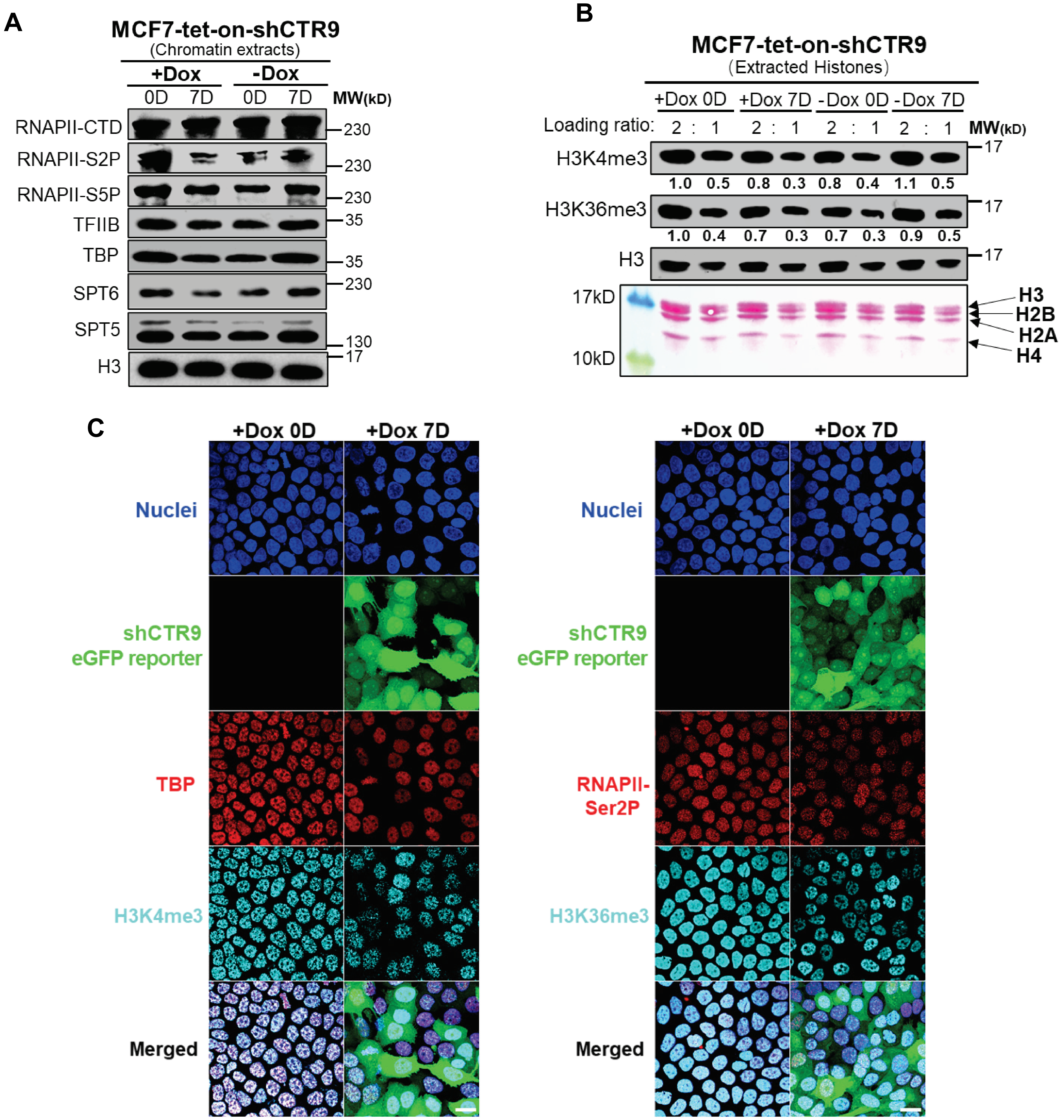


## Supplemental Figure S9. (Related to Discussion)

**A.** Western blot analyses of general transcription initiation and elongation factors in chromatin fractions of MCF7-tet-on-shCTR9 cells under indicated treatment conditions. Histone H3 was used as a loading control.

**B.** Western blot analyses of H3K4me3 and H3K36me3 on purified histones from MCF7-tet-on-shCTR9 cells under indicated treatment conditions (*Top*). Ponceau S staining of histones were shown in two-fold loading ratio (*Bottom*). The bands intensity of H3K4me3 and H3K36me3 are quantified by ImagePro after normalizing with H3 loading controls.

**C.** Representative images of Immuno-fluorescence staining of H3K4me3 (cyan)/ nuclei (blue) /TBP (red) (*left*) and H3K36me3 (cyan)/ nuclei (blue) /RNAPII-S2P (red) (*right*) in MCF7-tet-on–shCTR9 cells before and after Dox treatment. 100x scale bar shown at the bottom right applies to all images.

# Supplemental Figure S10

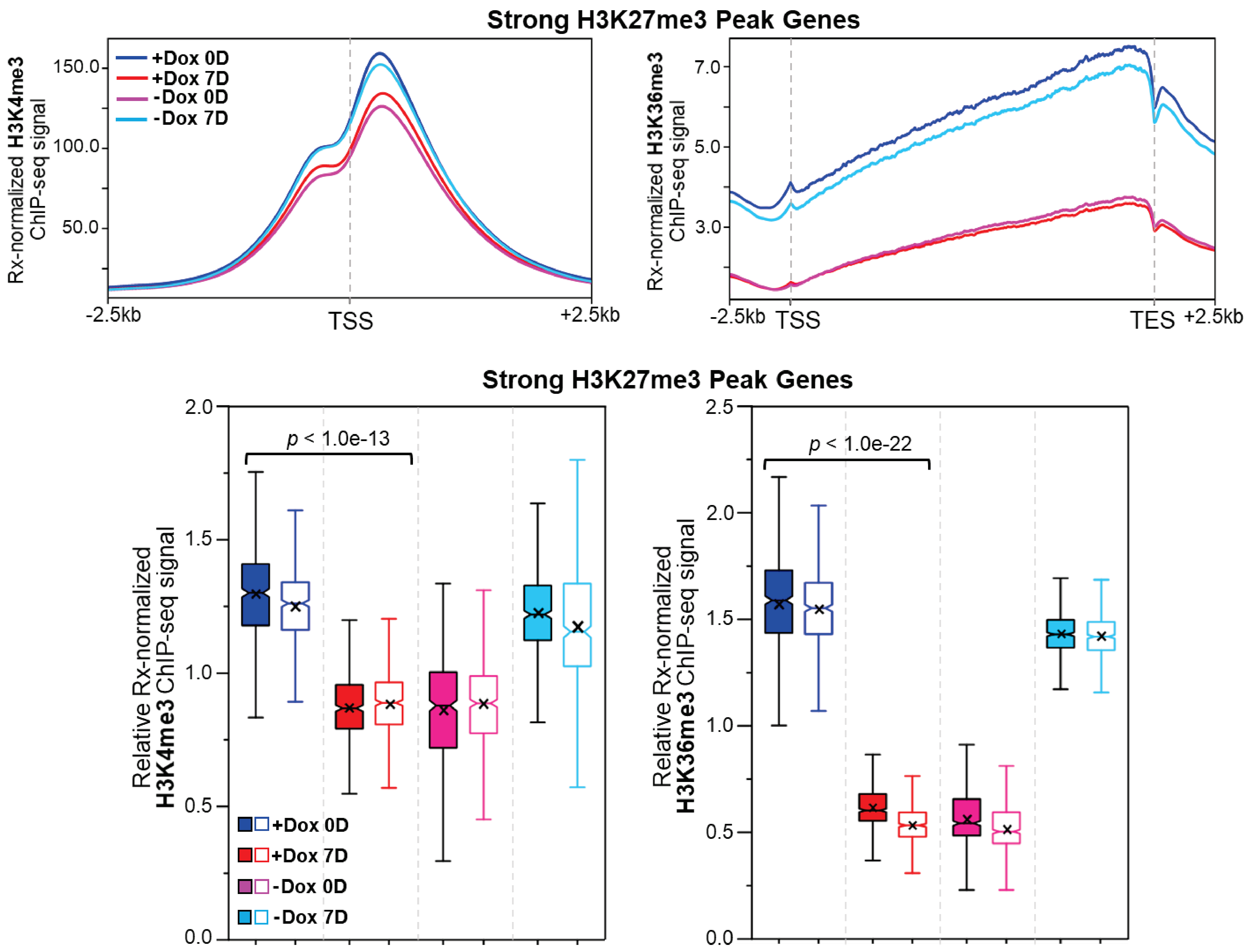


## Supplemental Figure S10. (Related to Discussion)

Average profiles of Rx-normalized H3K4me3 (*left*) and H3K36me3 (*right*) ChIP-seq signals at ±2.5kb of TSS or TSS-TES regions of the strong H3K27me3 peak associated genes (n=11,483) (*Top*). Notched boxplot of relative Rx-normalized H3K4me3 (*left*) and H3K36me3 (*right*) ChIP-seq signals on the strong H3K27me3 peak genes (n=2) (*Bottom*). Paired Student's t test was used to calculate statistical significance among groups.

# Supplemental Figure S11

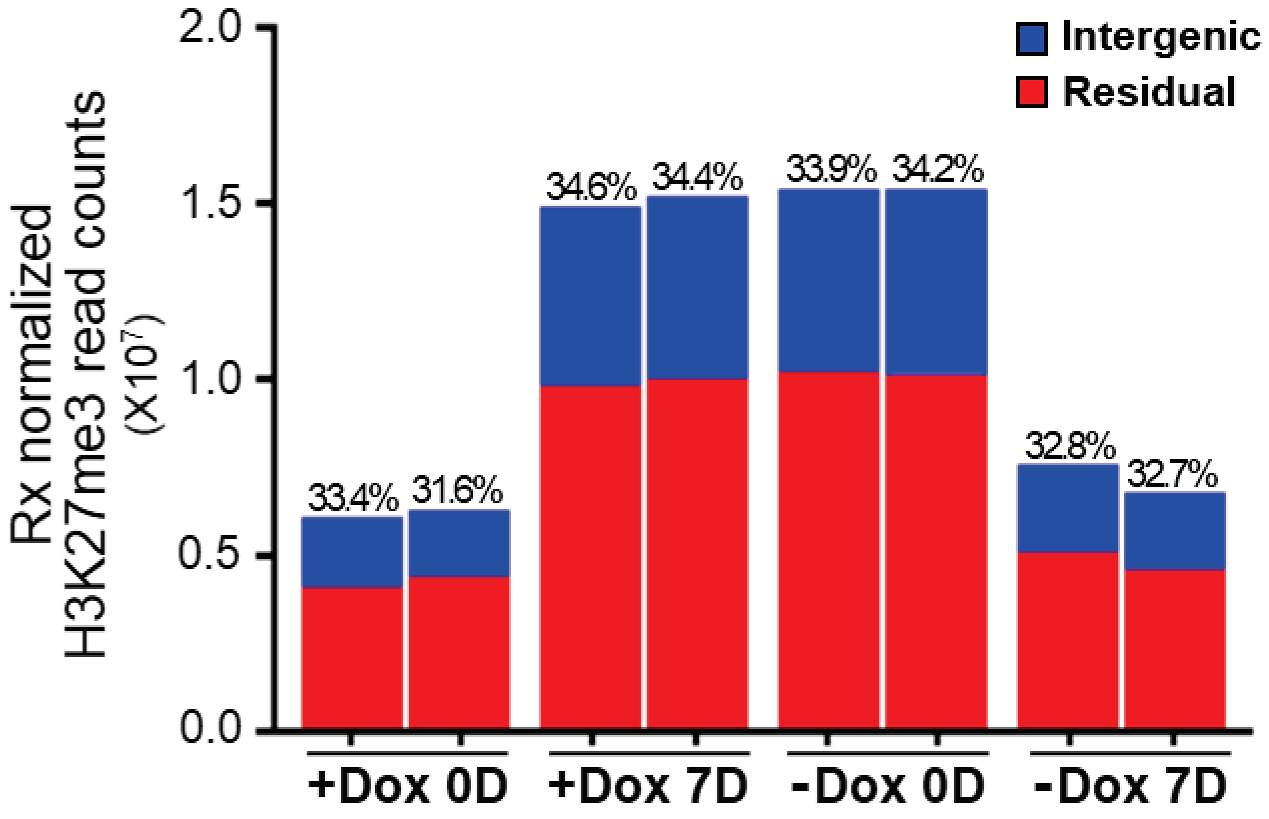


**Supplemental Figure S11. (Related to Discussion)**

Rx-normalized H3K27me3 ChIP-seq read counts in intergenic regions (blue) as well as residual genomic regions (red) upon Dox addition and removal in MCF7-tet-on-shCTR9 cells. Each bar represents individual biological replicate. Percentage (%) of intergenic read counts were shown on the top of each bar.

# Supplemental Figure S12

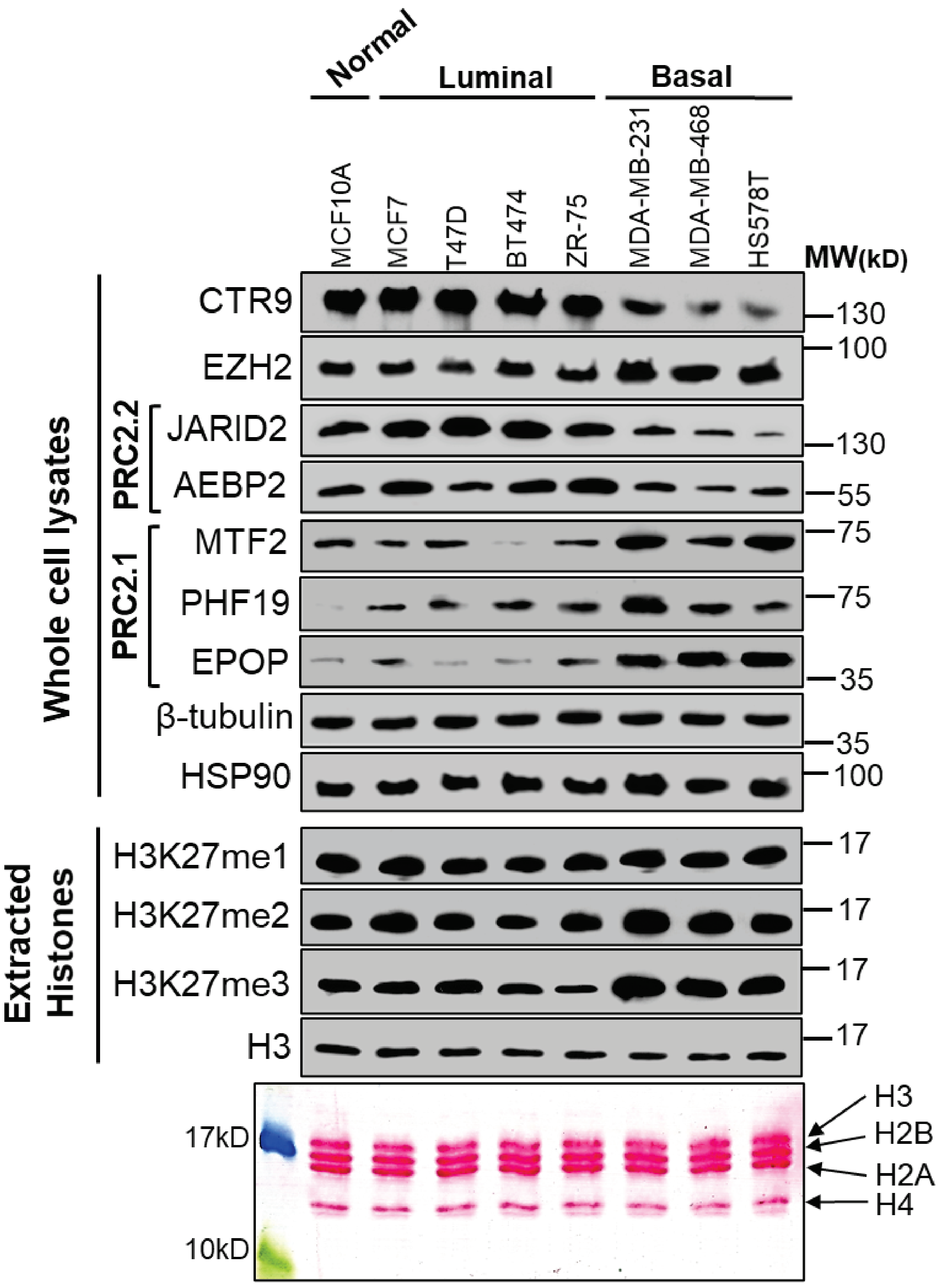


## Supplemental Figure S12. (Related to Discussion)

Western blot analyses of CTR9, EZH2, JARID2/AEBP2 (PRC2.2 subunits), MTF2/PHF19/EPOP (PRC2.1 subunits) and H3K27me1,2,3 in an immortalized non-transformed mammary epithelial cell line (MCF10A), four ER-positive human breast cancer cell lines, and three TNBC cell lines. β-tubulin/HSP90 and histone H3 served loading controls for whole cell lysates and extracted histones, respectively (*Top*). Ponceau S staining of acid extracted histone from above mentioned cell lines (*Bottom*).

# Supplemental Table S1

- **Flag ssDNA:**
  GCTGCGGAGCGGCGGGGCGAGACACTTGCTCGCCTTTTGACCCCATCATGGACTACAAAGACCATGA
  CGGTGATTATAAAGATCATGACATCGATTACAAGGATGACGATGACAAGCTCATGTCGCGGGGCTCCAT
  CGAGATTCCCCTCCGGGACACTGACGAGGTAAGTGTCGTGTATGG
- **gRNA sequence:** CTTTTGACCCCATCATGTCG
- **Flag-seq Forward:** TTGTTTAAGCGGCTGACGGG
- **Flag-seq Reverse:** CCCGGAGGGGAATCTCGATG

**Supplemental Table S1 (Related to Materials & Methods):** Primers and oligonucleotides for construction and validation of MCF7-3xFlag-KI-CTR9 cell line

# Supplemental Table S2 (refer to following pages)

# Supplemental Table S3

- Table of 11,483 strong H3K27me3 peak genes (Related to Fig. S2F)

# Supplemental Table S4 (refer to following pages)

# Supplemental Table S5

- Table of 17,212 SUZ12 peak genes (Related to Fig. S3C)

# Supplemental Table S6

- Table of 6,750 confident Flag-CTR9 binding genes (Related to Fig. 4C)

# Supplemental Table S7 (refer to following pages)

# Supplemental Table S8

- Table of 240 previously identified CTR9 regulated genes
  (Related to Fig. 4G)

# Supplemental Table S2

| Peptide Sequence | Peptide Peak Area in LC-MS/MS | | | | | |
|---|---|---|---|---|---|---|
| **H3(3-8)** | **+Dox 0D**-Rep1 | **+Dox 0D**-Rep2 | **+Dox 0D**-Rep3 | **+Dox 7D**-Rep1 | **+Dox 7D**-Rep2 | **+Dox 7D**-Rep3 |
| T$K_4$QTAR | 2.890E+10 | 2.990E+10 | 3.230E+10 | 1.800E+10 | 8.970E+09 | 1.230E+10 |
| T$K_4$(me1)QTAR | 4.180E+09 | 4.230E+09 | 4.580E+09 | 2.100E+09 | 1.050E+09 | 1.420E+09 |
| T$K_4$(me2)QTAR | 4.860E+08 | 5.230E+08 | 5.580E+08 | 2.330E+08 | 1.220E+08 | 1.730E+08 |
| T$K_4$(me3)QTAR | 1.630E+08 | 1.650E+08 | 2.290E+08 | 1.160E+08 | 6.610E+07 | 8.120E+07 |
| T$K_4$(ac)QTAR | 5.950E+07 | 6.730E+07 | 6.840E+07 | 1.980E+07 | 3.710E+06 | 2.770E+06 |
| **H3(9-17)** | **+Dox 0D**-Rep1 | **+Dox 0D**-Rep2 | **+Dox 0D**-Rep3 | **+Dox 7D**-Rep1 | **+Dox 7D**-Rep2 | **+Dox 7D**-Rep3 |
| $K_9$STGG$K_{14}$APR | 3.860E+09 | 4.160E+09 | 4.650E+09 | 3.570E+09 | 1.980E+09 | 2.150E+09 |
| $K_9$(me1)STGG$K_{14}$APR | 2.340E+09 | 2.510E+09 | 2.900E+09 | 1.730E+09 | 1.290E+09 | 1.210E+09 |
| $K_9$(me2)STGG$K_{14}$APR | 4.350E+09 | 4.810E+09 | 5.460E+09 | 3.290E+09 | 2.210E+09 | 2.370E+09 |
| $K_9$(me3)STGG$K_{14}$APR | 2.690E+09 | 2.960E+09 | 3.240E+09 | 2.120E+09 | 1.370E+09 | 2.070E+09 |
| $K_9$STGG$K_{14}$(ac)APR | 3.890E+09 | 3.970E+09 | 3.480E+09 | 2.540E+09 | 2.460E+09 | 2.210E+09 |
| $K_9$(me1)STGG$K_{14}$(ac)APR | 2.850E+09 | 2.920E+09 | 2.620E+09 | 1.420E+09 | 1.420E+09 | 1.350E+09 |
| $K_9$(me2)STGG$K_{14}$(ac)APR | 5.490E+09 | 5.570E+09 | 5.080E+09 | 2.540E+09 | 2.550E+09 | 2.230E+09 |
| $K_9$(me3)STGG$K_{14}$(ac)APR | 2.140E+09 | 2.240E+09 | 2.110E+09 | 1.180E+09 | 1.090E+09 | 1.210E+09 |
| $K_9$(ac)STGG$K_{14}$(ac)APR | 2.860E+08 | 2.030E+08 | 2.290E+08 | 9.940E+07 | 8.550E+07 | 7.320E+07 |
| **H3(18-26)** | **+Dox 0D**-Rep1 | **+Dox 0D**-Rep2 | **+Dox 0D**-Rep3 | **+Dox 7D**-Rep1 | **+Dox 7D**-Rep2 | **+Dox 7D**-Rep3 |
| $K_{18}$QLAT$K_{23}$AAR | 1.090E+10 | 1.070E+10 | 1.240E+10 | 6.540E+09 | 7.260E+09 | 4.870E+09 |
| $K_{18}$QLAT$K_{23}$(me1)AAR | 1.810E+07 | 2.320E+07 | 3.090E+07 | 1.320E+07 | 2.020E+07 | 1.550E+07 |
| $K_{18}$QLAT$K_{23}$(ac)AAR | 8.180E+09 | 6.970E+09 | 5.510E+09 | 3.400E+09 | 3.980E+09 | 3.910E+09 |
| $K_{18}$(ac)QLAT$K_{23}$(ac)AAR | 3.160E+08 | 3.190E+08 | 3.320E+08 | 1.330E+08 | 2.110E+08 | 1.750E+08 |
| **H3(27-40)** | **+Dox 0D**-Rep1 | **+Dox 0D**-Rep2 | **+Dox 0D**-Rep3 | **+Dox 7D**-Rep1 | **+Dox 7D**-Rep2 | **+Dox 7D**-Rep3 |
| $K_{27}$SAPATGGV$K_{36}$KPHR | 8.320E+08 | 9.660E+08 | 7.520E+08 | 1.450E+07 | 6.470E+07 | 5.270E+07 |
| $K_{27}$(me1)SAPATGGV$K_{36}$KPHR | 2.486E+08 | 2.715E+08 | 2.089E+08 | 1.622E+07 | 9.851E+07 | 7.908E+07 |
| $K_{27}$(me2)SAPATGGV$K_{36}$KPHR | 3.472E+08 | 3.568E+08 | 3.462E+08 | 4.823E+08 | 4.270E+08 | 3.583E+08 |
| $K_{27}$(me3)SAPATGGV$K_{36}$KPHR | 2.327E+08 | 2.446E+08 | 3.185E+08 | 2.813E+08 | 2.727E+08 | 2.146E+08 |
| $K_{27}$SAPATGGV$K_{36}$(me1)KPHR | 7.484E+08 | 8.785E+08 | 6.551E+08 | 4.048E+07 | 4.849E+07 | 4.392E+07 |
| $K_{27}$(me1)SAPATGGV$K_{36}$(me1)KPHR | 6.234E+08 | 7.498E+08 | 5.369E+08 | 2.470E+07 | 1.990E+08 | 1.659E+08 |
| $K_{27}$(me2)SAPATGGV$K_{36}$(me1)KPHR | 4.867E+08 | 4.709E+08 | 3.288E+08 | 4.963E+08 | 4.877E+08 | 4.084E+08 |
| $K_{27}$(me3)SAPATGGV$K_{36}$(me1)KPHR | 6.152E+07 | 8.251E+07 | 6.858E+07 | 7.768E+07 | 9.330E+07 | 8.818E+07 |
| $K_{27}$SAPATGGV$K_{36}$(me2)KPHR | 1.019E+09 | 1.253E+09 | 9.568E+08 | 1.299E+07 | 5.506E+07 | 5.083E+07 |
| $K_{27}$(me1)SAPATGGV$K_{36}$(me2)KPHR | 3.863E+08 | 5.891E+08 | 3.952E+08 | 2.769E+07 | 1.203E+08 | 1.016E+08 |
| $K_{27}$(me2)SAPATGGV$K_{36}$(me2)KPHR | 4.570E+08 | 5.370E+08 | 3.890E+08 | 5.380E+08 | 7.000E+08 | 5.810E+08 |
| $K_{27}$(me3)SAPATGGV$K_{36}$(me2)KPHR | 6.445E+07 | 7.057E+07 | 4.050E+07 | 1.444E+08 | 1.652E+08 | 1.349E+08 |
| $K_{27}$SAPATGGV$K_{36}$(me3)KPHR | 1.903E+08 | 2.564E+08 | 7.448E+07 | 5.358E+06 | 2.928E+07 | 1.541E+07 |
| $K_{27}$(me1)SAPATGGV$K_{36}$(me3)KPHR | 7.048E+07 | 8.149E+07 | 6.742E+07 | 2.372E+07 | 9.570E+07 | 6.882E+07 |
| $K_{27}$(me2)SAPATGGV$K_{36}$(me3)KPHR | 1.525E+07 | 2.213E+07 | 1.760E+07 | 2.543E+07 | 2.177E+07 | 1.906E+07 |
| $K_{27}$(ac)SAPATGGV$K_{36}$KPHR | 3.630E+07 | 3.300E+07 | 3.660E+07 | 1.140E+07 | 1.070E+07 | 9.190E+06 |
| $K_{27}$(ac)SAPATGGV$K_{36}$(me1)KPHR | 3.430E+07 | 2.650E+07 | 2.770E+07 | 4.160E+06 | 4.870E+06 | 3.040E+06 |
| $K_{27}$(ac)SAPATGGV$K_{36}$(me2)KPHR | 7.860E+07 | 6.380E+07 | 7.690E+07 | 1.440E+07 | 1.340E+07 | 1.200E+07 |
| $K_{27}$(ac)SAPATGGV$K_{36}$(me3)KPHR | 1.980E+07 | 1.200E+07 | 1.220E+07 | 6.250E+06 | 6.120E+06 | 7.270E+06 |
| **H3(73-83)** | **+Dox 0D**-Rep1 | **+Dox 0D**-Rep2 | **+Dox 0D**-Rep3 | **+Dox 7D**-Rep1 | **+Dox 7D**-Rep2 | **+Dox 7D**-Rep3 |
| EIAQDF$K_{79}$TDLR | 3.160E+09 | 1.910E+09 | 2.060E+09 | 1.580E+08 | 3.420E+09 | 3.380E+09 |
| EIAQDF$K_{79}$(me1)TDLR | 3.500E+08 | 3.310E+08 | 3.580E+08 | 2.280E+08 | 5.450E+08 | 5.170E+08 |
| EIAQDF$K_{79}$(me2)TDLR | 6.410E+07 | 6.410E+07 | 6.450E+07 | 5.790E+07 | 9.290E+07 | 8.870E+07 |

## Supplemental Table S2. (Related to Fig. 1B)

Summary of the peptide intensity for each detected histone peptide species for corresponding histone modifications reported in Figure 1B as measured by quantitative tandem mass spectrometry analysis.

# Supplemental Table S4

| Top 15 Epigenetic modulators | Wilcoxon Test statistic | Wilcoxon P-value | Z-score | max AUC |
|---|---|---|---|---|
| EED | 2.982 | 1.43E-03 | 3.047 | 0.813 |
| SSRP1 | 2.887 | 1.95E-03 | 3.995 | 0.683 |
| RING1B | 4.114 | 1.95E-05 | 2.642 | 0.828 |
| TET2 | 3.763 | 8.38E-05 | 2.566 | 0.773 |
| EZH2 | 10.499 | 4.37E-26 | 1.925 | 0.95 |
| CTCF | 25.995 | 2.82E-149 | 1.998 | 0.752 |
| G9a | 2.541 | 5.53E-03 | 2.928 | 0.62 |
| KDM4A | 2.81 | 2.48E-03 | 2.223 | 0.704 |
| SUZ12 | 4.933 | 4.05E-07 | 1.349 | 0.941 |
| JARID2 | 5.443 | 2.62E-08 | 1.308 | 0.955 |
| JMJD6 | 3.3 | 4.84E-04 | 1.383 | 0.75 |
| HIRA | 1.77 | 3.83E-02 | 2.037 | 0.751 |
| CBX3 | 1.665 | 4.79E-02 | 2.88 | 0.613 |
| KDM2B | 5.118 | 1.54E-07 | 0.743 | 0.752 |

| Top 15 Transcription Factors | Wilcoxon Test statistic | Wilcoxon P-value | Z-score | max AUC |
|---|---|---|---|---|
| YBX1 | 36.25 | 1.45E-04 | 3.63 | 0.769 |
| REST | 99.23 | 1.66E-23 | 3.442 | 0.709 |
| BMI1 | 45.07 | 3.29E-06 | 3.29 | 0.739 |
| ZBTB48 | 58.19 | 2.97E-09 | 2.939 | 0.722 |
| TSC22D4 | 26.71 | 3.78E-03 | 3.313 | 0.746 |
| ASCL1 | 59.73 | 1.16E-09 | 2.896 | 0.716 |
| ZTA | 23.94 | 8.34E-03 | 3.417 | 0.736 |
| ZNF202 | 24.32 | 7.51E-03 | 3.02 | 0.739 |
| ZNF207 | 20.32 | 2.11E-02 | 3.393 | 0.744 |
| GTF2I | 49.62 | 3.50E-07 | 2.51 | 0.722 |
| CRY1 | 17.08 | 4.39E-02 | 3.565 | 0.777 |
| ZNF707 | 22.64 | 1.18E-02 | 3.451 | 0.678 |
| TCF7L1 | 38.87 | 5.08E-05 | 3.063 | 0.64 |
| IKZF1 | 43.51 | 6.79E-06 | 2.34 | 0.775 |

## Supplemental Table S4. (Related to Fig. 2F)

*BART* (Binding Analysis for Regulation of Transcription) results of 11,483 strong H3K27me3 peak genes. Higher the ‘Wilcoxon Test statistic’ is, more likely this factor regulates the input genes. Higher the ‘max AUC’, greater association of the dataset with the input genes.

# Supplemental Table S7

| Top 15 Epigenetic modulators | Wilcoxon Test statistic | Wilcoxon P-value | Z-score | max AUC |
|---|---|---|---|---|
| MBD2 | 3.567 | 1.80E-04 | 2.4 | 0.899 |
| SUZ12 | 5.595 | 1.10E-08 | 1.582 | 0.908 |
| EZH2 | 6.979 | 1.49E-12 | 1.262 | 0.917 |
| TET2 | 2.959 | 1.54E-03 | 2.078 | 0.889 |
| KDM4A | 2.544 | 5.49E-03 | 1.948 | 0.895 |
| JARID2 | 5.042 | 2.31E-07 | 1.19 | 0.909 |
| KDM2B | 5.546 | 1.46E-08 | 0.955 | 0.939 |
| EZH1 | 2.81 | 2.48E-03 | 1.223 | 0.917 |
| INO80 | 2.916 | 1.78E-03 | 1.096 | 0.919 |
| TET3 | 1.772 | 3.82E-02 | 1.168 | 0.921 |
| RNF2 | 1.871 | 3.07E-02 | 1.089 | 0.921 |
| HDAC2 | 2.508 | 6.07E-03 | 1.193 | 0.89 |
| BMI1 | 1.678 | 4.67E-02 | 1.524 | 0.891 |
| BCL11B | 3.041 | 1.18E-03 | 0.733 | 0.893 |
| KDM5B | 2.115 | 1.72E-02 | 1.007 | 0.891 |
| | | | | |
| **Top 15 Transcription Factors** | Wilcoxon Test statistic | Wilcoxon P-value | Z-score | max AUC |
| ZFX | 3.896 | 4.88E-05 | 1.63 | 0.93 |
| SP1 | 4.253 | 1.06E-05 | 1.718 | 0.911 |
| ZNF202 | 1.85 | 3.22E-02 | 2.373 | 0.93 |
| ZBTB48 | 4.554 | 2.63E-06 | 2.364 | 0.87 |
| TRIM25 | 2.267 | 1.17E-02 | 1.984 | 0.896 |
| WDR5 | 3.006 | 1.32E-03 | 1.059 | 0.928 |
| ZBTB7A | 4.663 | 1.56E-06 | 1.255 | 0.89 |
| MYC | 8.573 | 5.04E-18 | 0.698 | 0.924 |
| ZNF263 | 2.495 | 6.30E-03 | 1.802 | 0.873 |
| GTF2I | 2.653 | 3.99E-03 | 1.569 | 0.876 |
| KLF9 | 3.279 | 5.21E-04 | 1.05 | 0.888 |
| ZNF687 | 1.986 | 2.35E-02 | 1.827 | 0.869 |
| ZMYND8 | 2.419 | 7.78E-03 | 1.567 | 0.869 |
| ZNF592 | 1.854 | 3.19E-02 | 2.421 | 0.857 |
| SPDEF | 1.399 | 8.10E-02 | 1.834 | 0.893 |

**Supplemental Table S7. (Related to Fig. 4D)**

*BART* (Binding Analysis for Regulation of Transcription) results of 6,750 confident Flag-CTR9 binding genes.
